# Supplementary material for: Tubular insulin-induced gene 1 deficiency promotes NAD+ consumption and exacerbates kidney fibrosis
Source: EMBO Mol Med. 2024 May 28;16(7):11. doi: 10.1038/s44321-024-00081-7 (PMC11251182; doi:10.1038/s44321-024-00081-7)
Supplement: Supplementary file 1 — Table EV1 [file 44321_2024_81_MOESM1_ESM.docx]

**Table EV1. The basic information and diagnosis of renal biopsy specimens.**

| Number | Sex | Age, yr | Pathological diagnosis | Serum creatinine, μmol/l | Estimated glomerular filtration rate, ml/min per 1.73m^2^ |
| --- | --- | --- | --- | --- | --- |
| 4198 | Girl | 13 | Focal segmental glomerulosclerosis with glomerular abandonment | 63 | 85.18813333 |
| 4621 | Girl | 16 | IgA nephropathy | 41 | 148.7082049 |
| 4195 | Boy | 8 | Mild mesangial hyperplasia with glomerular abandonment and segmental sclerosis | 36.8 | 128.9727174 |
| 4183 | Girl | 8 | Lupus nephritis IV+V | 73 | 67.5170137 |
| 4155 | Boy | 5 | Thrombotic microvascular disease with IgA deposition in the mesangial area | 54 | 74.37059259 |
| 4126 | Boy | 9 | Focal segmental glomerulosclerosis | 84 | 57.3716 |
| 3940 | Boy | 8 | Subacute tubulointerstitial nephropathy | 387 | 13.20746253 |
| 3263 | Boy | 12 | Purpura nephritis with renal tubulointerstitial injury | 44 | 132.7607273 |
| 3157 | Boy | 7 | IgA nephropathy | N/A | N/A |
| 4638 | Boy | 9 | IgA nephropathy | 53 | 96.43939623 |
| 4613 | Boy | 6 | Sclerosing glomerulonephritis | 149 | 29.40338255 |
| 4576 | Boy | 15 | Chronic renal tubulointerstitial lesions with partial glomerular ischemic changes | 55 | 114.1742255 |
| 4570 | Girl | 18 | Mesangium hyperplasia with focal glomerulosclerosis | 72 | 85.18813333 |
| 4563 | Boy | 15 | ANCA associated vasculitis renal damage | 57 | 110.1681123 |
| 4547 | Girl | 11 | Focal segmental glomerulosclerosis with tubulointerstitial damage | 114 | 44.83585965 |
