# Supplementary material for: Tubular insulin-induced gene 1 deficiency promotes NAD+ consumption and exacerbates kidney fibrosis
Source: EMBO Mol Med. 2024 May 28;16(7):11. doi: 10.1038/s44321-024-00081-7 (PMC11251182; doi:10.1038/s44321-024-00081-7)
Supplement: Supplementary file 2 — Table EV2 [file 44321_2024_81_MOESM2_ESM.docx]

**Table EV2.** **The information of plasmids or siRNA.**

| si-m-Insig1-1:  GCTTATTGTATCCCTGTAT | RIBOBIO | siG2101270150560300 |
| --- | --- | --- |
| si-m-Insig1-2:  GCCAATAATGTGCAGCTGT | RIBOBIO | siG2101270150561392 |
| si-m-Insig1-3:  CGGAAAAGCCTCACAGTGA | RIBOBIO | siB1908070124575037 |
| si-h-Insig1:  CCCAGATTTCCTCTATATT | RIBOBIO | stB0006838A |
| si-m-Aldh1a1_001:  GGCACTCAATGGTGGGAAA | RIBOBIO | siG150529065054 |
| si-m-Aldh1a1_002:  GGGTTAACTGCTATATGAT | RIBOBIO | siG150529065113 |
| si-m-Aldh1a1_003:  CAATGCTCATGTTCATTTG | RIBOBIO | siG150529065128 |
| pLenti-CMV-mInsig1*Flag-GFP-Puro | PPL | PPL50212-4a |
| pLVX-Puro-mAldh1a1 | PPL | PPL50193-4a |
| pLVX-Puro-mAldh1a1(K193Q) | PPL | PPL50193-4c |

pGL4.19-mAldh1a1 promoter(-2000~+100) PPL PPL50193-2a
