# Supplementary material for: Tubular insulin-induced gene 1 deficiency promotes NAD+ consumption and exacerbates kidney fibrosis
Source: EMBO Mol Med. 2024 May 28;16(7):11. doi: 10.1038/s44321-024-00081-7 (PMC11251182; doi:10.1038/s44321-024-00081-7)
Supplement: Supplementary file 3 — Table EV3 [file 44321_2024_81_MOESM3_ESM.docx]

**Table EV3. Primers sequences for RT-PCR.**

| Gene | Sequence |
| --- | --- |
| Mouse Insig1 (F) | CTAGTGCTCTTCTCATTTGGCG |
| Mouse Insig1 (R) | AGGGATACAGTAAACCGACAACA |
| Mouse Fn (F) | GCAAGAAGGACAACCGAGGAAA |
| Mouse Fn (R) | GGACATCAGTGAAGGAGCCAGA |
| Mouse Acta2 (F) | CTGAAGAGCATCCGACAC |
| Mouse Acta2 (R) | GCCTGAATAGCCACATACA |
| Mouse Col1a1 (F) | TGGCTCCCTTGGACATTGGTG |
| Mouse Col1a1 (R) | AATTGAGTTTGGGTTGTTCGTCTGTTT |
| Mouse Col3a1 (F) | GGACCAGGCAATGATGGAAAAC |
| Mouse Col3a1 (R) | GGACCAGGGAAACCCATGACA |
| Mouse Gapdh (F) | AAGAAGGTGGTGAAGCAGG |
| Mouse Gapdh (R) | GAAGGTGGAAGAGTGGGAGT |
| Mouse Vim (F) | GCCAAGCAGGAGTCAAAC |
| Mouse Vim (R) | CTCTTCCATCTCACGCATC |
| Mouse Postn (F) | TGGTATCAAGGTGCTATCTGCG |
| Mouse Postn (R) | AATGCCCAGCGTGCCATAA |
| Mouse Ctgf (F) | GGGCCTCTTCTGCGATTTC |
| Mouse Ctgf (R) | ATCCAGGCAAGTGCATTGGTA |
| Mouse Aldh1a1 (F) | GAGGCACTCAATGGTGGGAA |
| Mouse Aldh1a1 (R) | GGCAGGGCCTATCTTCCAAA |
| Mouse Slc7a13 (F) | CAGTGCTGACCTTGACCA |
| Mouse Slc7a13 (R) | AGGCTGAACGCCATACTC |
| Mouse Cyp4b1 (F) | CCTACTGGTTCTGGAAGGG |
| Mouse Cyp4b1 (R) | CCGTGTGGACTCAGCAA |
| Mouse Cd74 (F) | CGGATGGCTACTCCCTTG |
| Mouse Cd74 (R) | TGGTCCTGGGTCATGTTG |
| Mouse Pdgfb (F) | TTTGCGGTTCTACCCGAT |
| Mouse Pdgfb (R) | GTCAATCCGTCCTCCCAA |
| Mouse Slc6a13 (F) | CAGGGTGTGCCCATATCT |
| Mouse Slc6a13 (R) | CCCAGGAGAACGACCAT |
| Mouse Trib3 (F) | GCCGCAGCACTTTAGCA |
| Mouse Trib3 (R) | CCAGAGGTGTAGCTCGCAT |
| Mouse Perk (F) | GCACTTTAGATGGACGAATCGC |
| Mouse Perk (R) | TGCTGAGGCTAGATGAAACCA |
| Mouse Atf6 (F) | AGCGCCCAAGACTCAAACC |
| Mouse Atf6 (R) | CTGTATGCTGATAATCGACTGCT |
| Mouse Tnf-α (F) | CAGACCCTCACACTCACAAACCAC |
| Mouse Tnf-α (R) | CCTTGTCCCTTGAAGAGAACCTG |
| Mouse IL-1β (F) | TGTGTTTTCCTCCTTGCCTCTGAT |
| Mouse IL-1β (R) | TGCTGCCTAATGTCCCCTTGAAT |
| Mouse Acc1 (F) | TCACGCCACCTTGTCAG |
| Mouse Acc1 (R) | GGGGAGTCACAGAAGCAG |
| Mouse Fas (F) | CAGTTCCAGCCATGAAGAG |
| Mouse Fas (R) | GGCAAAGAGAACACACCAG |
| Human Insig1 (F) | GAGGAAACTCGGCAATGA |
| Human Insig1 (R) | CCAAACCCACAAAACTGG |
| Human Acta2 (F) | AAAAGACAGCTACGTGGGTGA |
| Human Acta2 (R) | GCCATGTTCTATCGGGTACTTC |
| Human Col1a1 (F) | GAGGGCCAAGACGAAGACATC |
| Human Col1a1 (R) | CAGATCACGTCATCGCACAAC |
| Human Ctgf (F) | GGTGTGGCTTTAGGAGCA |
| Human Ctgf (R) | CAGTCTCTTGATGGCTGGA |
| Human Fn (F) | GAGAATAAGCTGTACCATCGCAA |
| Human Fn (R) | CGACCACATAGGAAGTCCCAG |
| Chip -625/-634 (F) | GGAGTTTCAGAGATTCCCAG |
| Chip -625/-634 (R) | GACTTTTCCTTTCTCAGCCTGG |
