# Supplementary material for: Tubular insulin-induced gene 1 deficiency promotes NAD+ consumption and exacerbates kidney fibrosis
Source: EMBO Mol Med. 2024 May 28;16(7):11. doi: 10.1038/s44321-024-00081-7 (PMC11251182; doi:10.1038/s44321-024-00081-7)
Supplement: Supplementary file 4 — Appendix [file 44321_2024_81_MOESM4_ESM.pdf]

Tubular insulin-induced gene 1 deficiency promotes NAD^+ consumption and exacerbates kidney fibrosis

Appendix Figure S1 (P2)

Appendix Figure S2 (P3)

Appendix Figure S3 (P5)

Appendix Figure S1

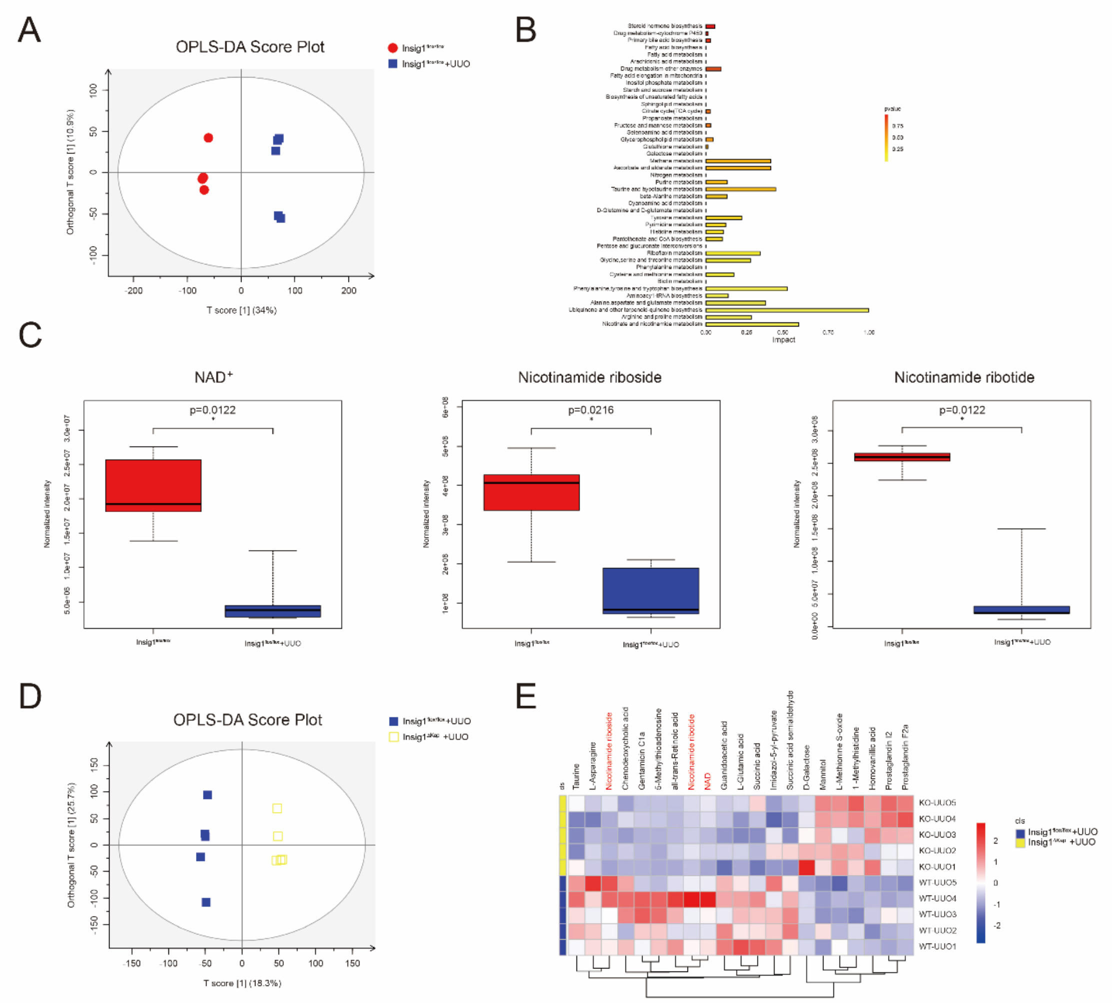

Appendix Figure S1. Insig1 deficiency in PTCs changed NAD$^+$ metabolic pathways.

A OPLS-DA analysis based on non-targeted metabolomics was conducted in the kidneys from Insig1$^{flox/flox}$ and Insig1$^{flox/flox}$ + UUO mice (n=5 in each group, biological replicates).

B The KEGG analysis among Insig1$^{flox/flox}$ and Insig1$^{flox/flox}$ + UUO group were analyzed and defined.

C The NAD$^+$ and its precursors (nicotinamide riboside and nicotinamide ribotide) concentrations in the UUO model (n=5 in each group, biological replicates).

D OPLS-DA analysis based on non-targeted metabolomics was conducted in the kidneys from Insig1$^{flox/flox}$ + UUO and Insig1$^{\Delta Kap}$ + UUO mice (n=5 in each group, biological replicates).

E Hierarchical cluster analysis was used to characterize the metabolites that differed between Insig1$^{flox/flox}$ + UUO and Insig1$^{\Delta Kap}$ + UUO mice (n=5 in each group, biological replicates).

Data information: Data are represented as mean ± SD. Student’s t-test.

Appendix Figure S2

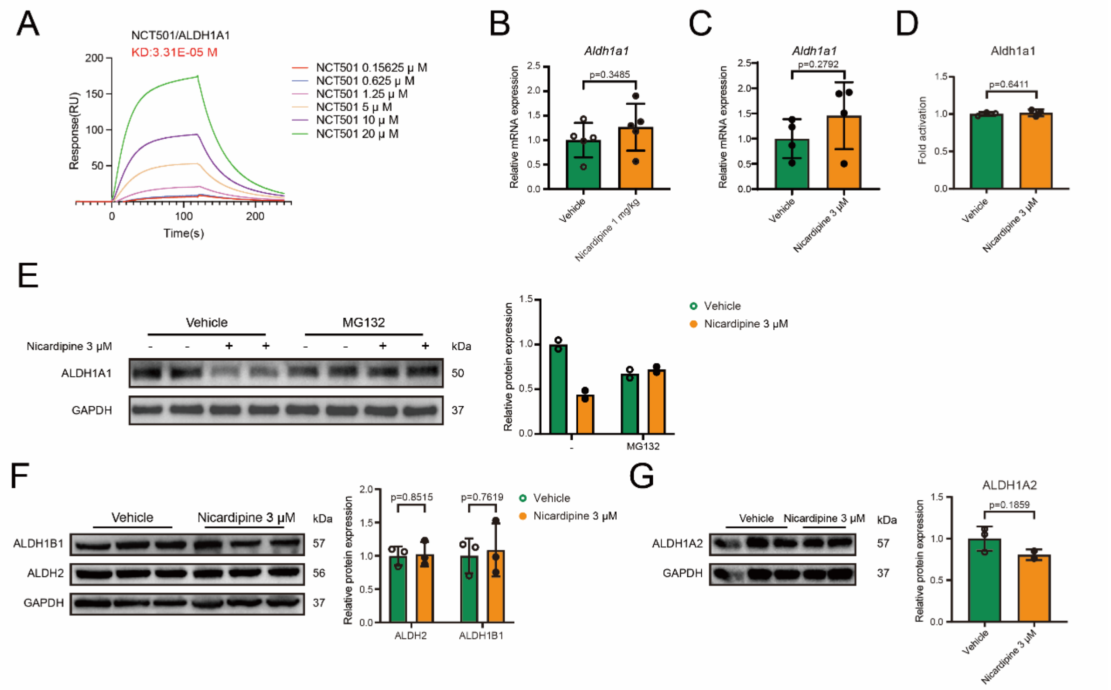

Appendix Figure S2. The expression of Aldh1a1 in nicardipine-treated mice and TKPTS cells.

A BIAcore was used to measure the binding of NCT501 to recombinant human Aldh1a1 protein.

B qPCR analysis for *Aldh1a1* expression in nicardipine-treated mice (n = 5 in each group, biological replicates).

C qPCR analysis for *Aldh1a1* expression in TKPTS cells with different concentrations of nicardipine (n = 4 in each group, biological replicates).

D Luciferase assay was performed to detect the transcriptional activity of Aldh1a1 in nicardipine-treated TKPTS cells (n = 3 in each group, biological replicates).

E Representative immunoblotted bands and quantification of ALDH1A1 in nicardipine-treated TKPTS cells with or without MG132 pretreatment (n = 2 in each group, biological replicates).

F,G Representative immunoblot bands and quantification of ALDH2, ALDH1B1, ALDH1A2 in nicardipine-treated TKPTS cells (n = 2-3 in each group, biological replicates).

Data information: Data are represented as mean ± SD. Student's t-test.

Appendix Figure S3

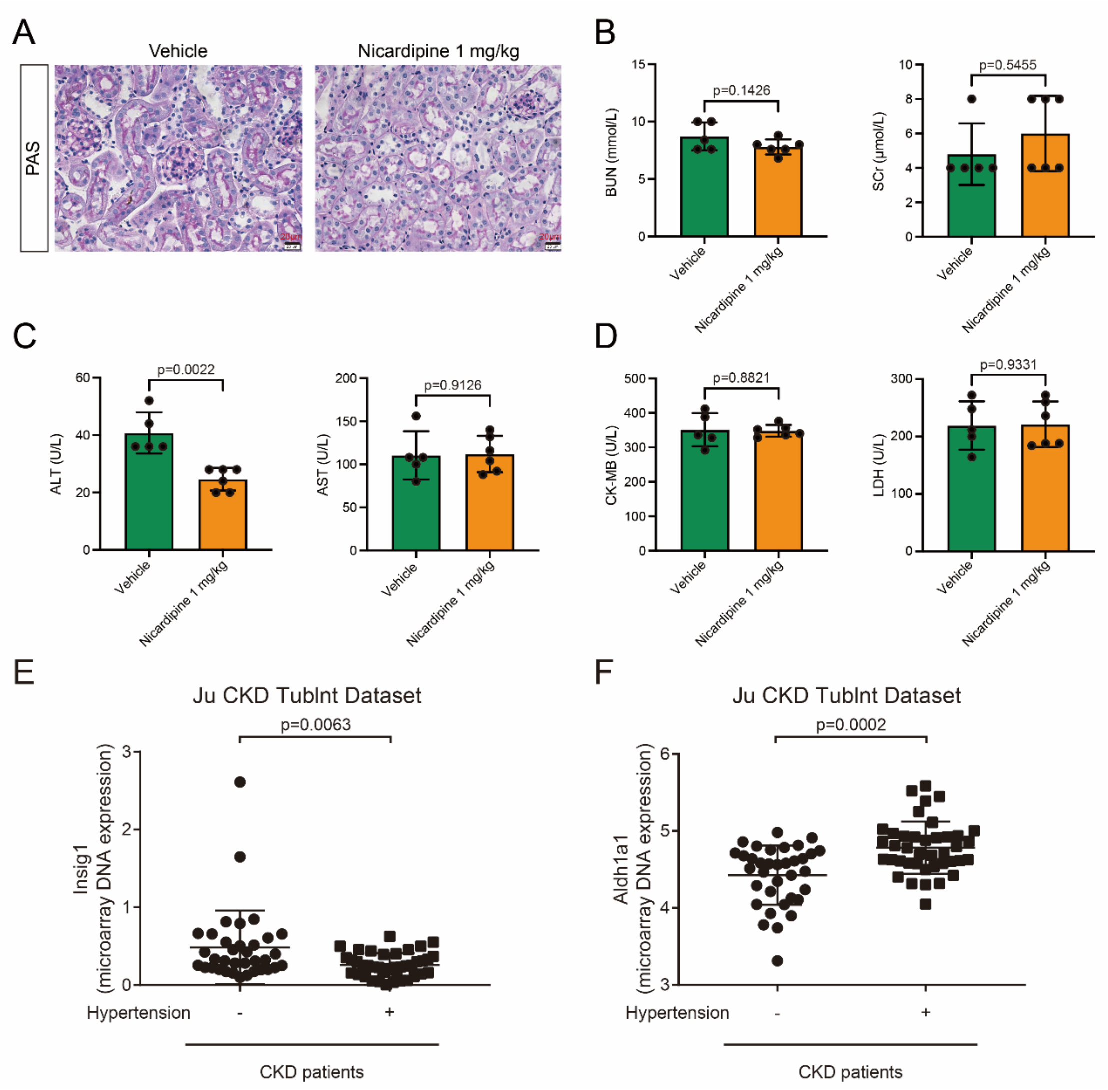

Appendix Figure S3. Assessment of toxicity in nicardipine-treated mice and the expressions of Insig1 and Aldh1a1 in CKD patients with hypertension.

A Representative images of PAS staining in the nicardipine-treated kidneys (scale bars, 20 μm; n = 5 in Sham group; n = 6 in Nicardipine group, biological replicates).

B BUN and SCr levels were measured in the nicardipine-treated kidneys (n = 5 in Sham group; n = 6 in Nicardipine group, biological replicates).

C ALT and AST levels were measured in the nicardipine-treated kidneys (n = 5 in Sham group; n = 6 in Nicardipine group, biological replicates).

D CK-MB and LDH levels were measured in the nicardipine-treated kidneys (n = 5 in Sham group; n = 6 in Nicardipine group, biological replicates).

E Correlation analysis of Insig1 expression and CKD patients with or without hypertension using Ju CKD Tublnt Dataset (no hypertension, n = 35; hypertension, n = 40).

F Correlation analysis of Aldh1a1 expression and CKD patients with or without hypertension using Ju CKD Tublnt Dataset (no hypertension, n = 35; hypertension, n = 40).

Data information: In (B, C), Data are represented as mean ± SD. Student's t-test or Mann Whitney test. In (D), Data are represented as mean ± SD. Student's t-test. In (E, F), Data are represented as mean ± SD. Mann Whitney test.
