# Supplementary figures and images for: Tubular insulin-induced gene 1 deficiency promotes NAD+ consumption and exacerbates kidney fibrosis (part 1 of 2)
Source: EMBO Mol Med. 2024 May 28;16(7):11. doi: 10.1038/s44321-024-00081-7 (PMC11251182; doi:10.1038/s44321-024-00081-7)

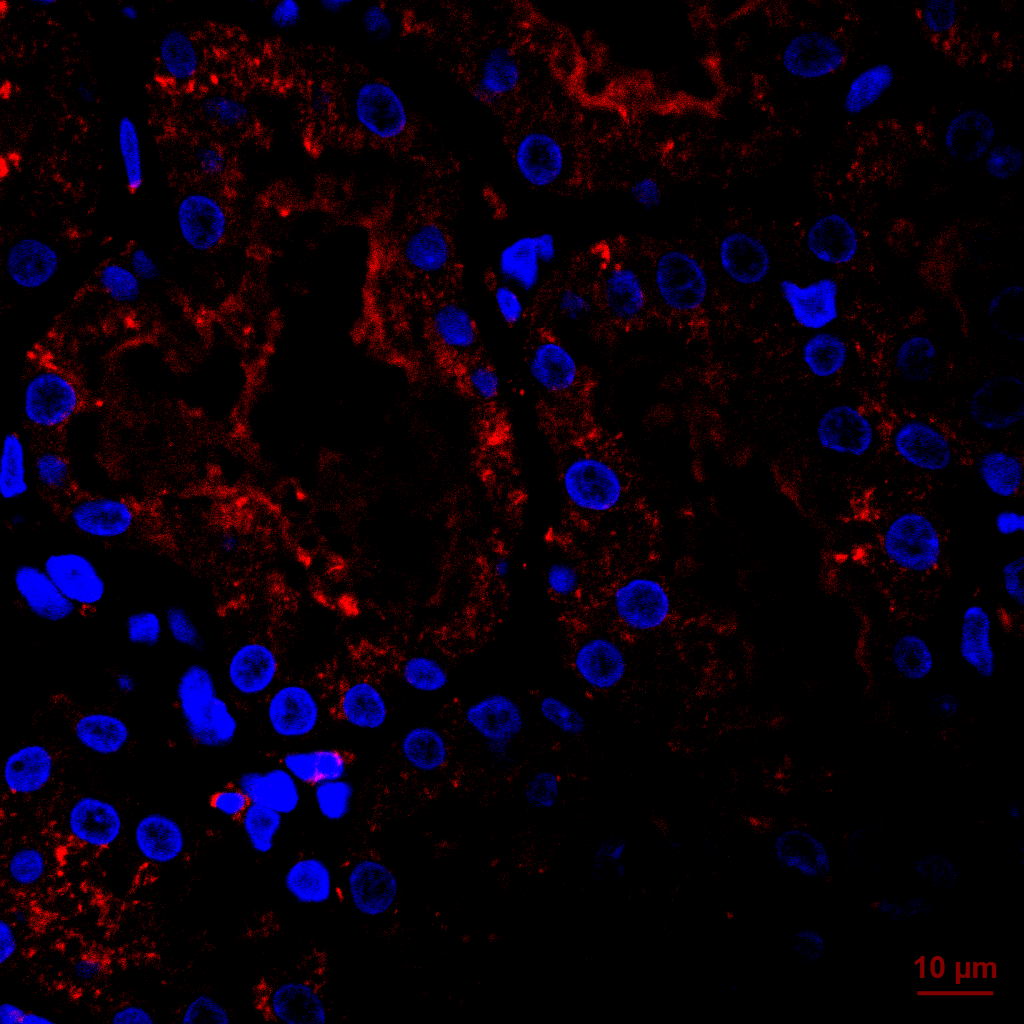

Supplement: Supplementary file 6 — Source data Fig. 1 [file 44321_2024_81_MOESM6_ESM.zip › Figure 1/1D/CKD IF.tif]

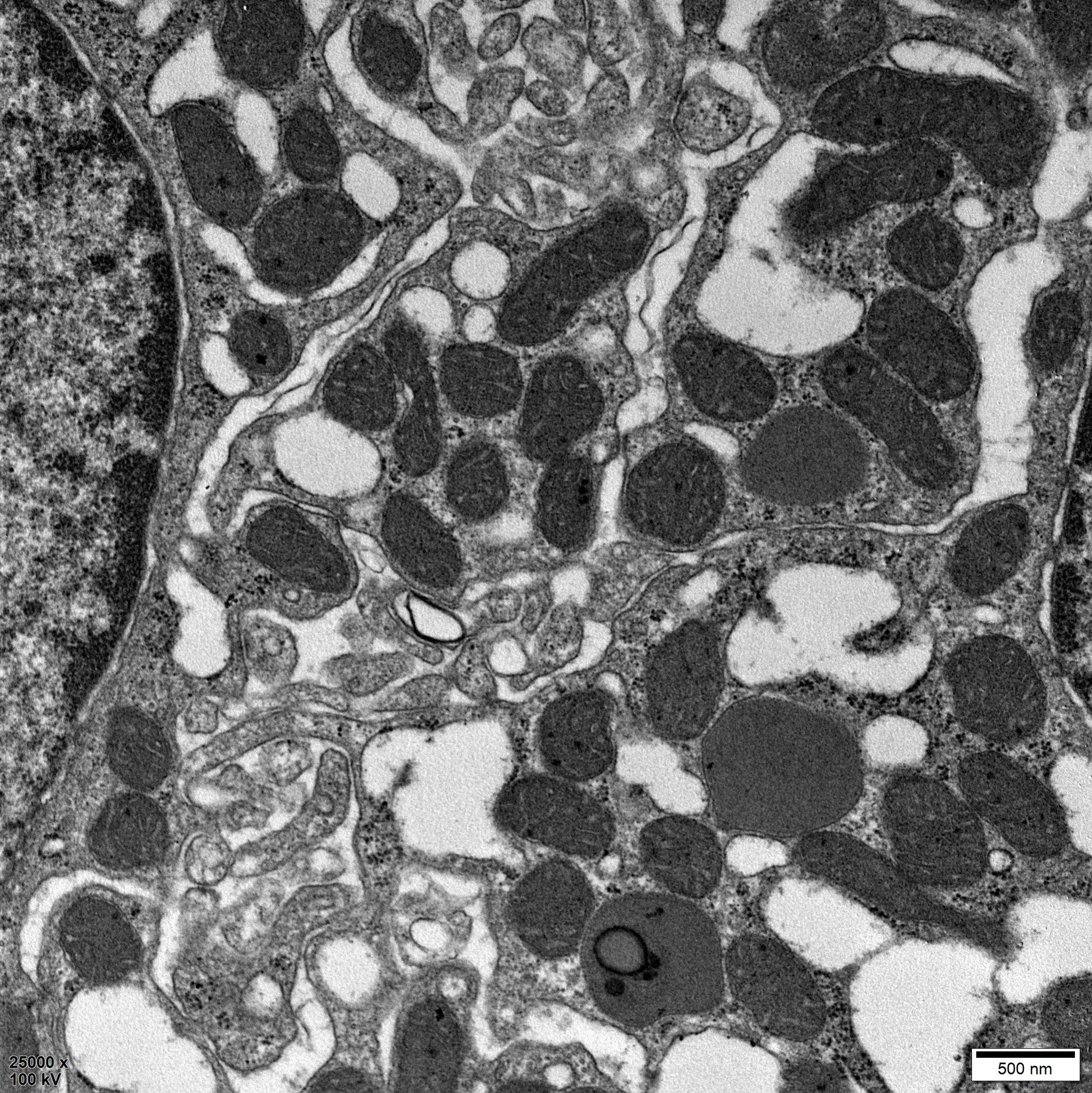

Supplement: Supplementary file 6 — Source data Fig. 1 [file 44321_2024_81_MOESM6_ESM.zip › Figure 1/1D/CKD TEM.jpg]

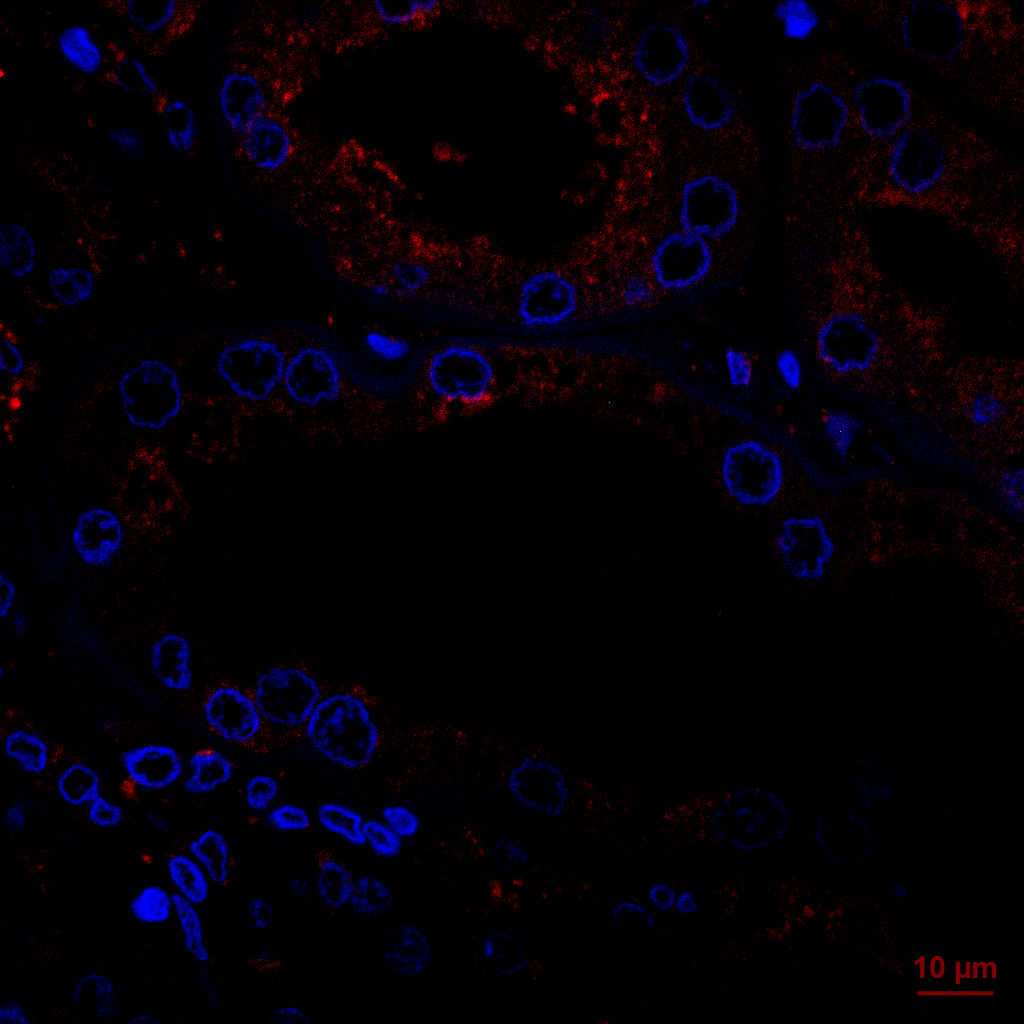

Supplement: Supplementary file 6 — Source data Fig. 1 [file 44321_2024_81_MOESM6_ESM.zip › Figure 1/1D/Health IF.tif]

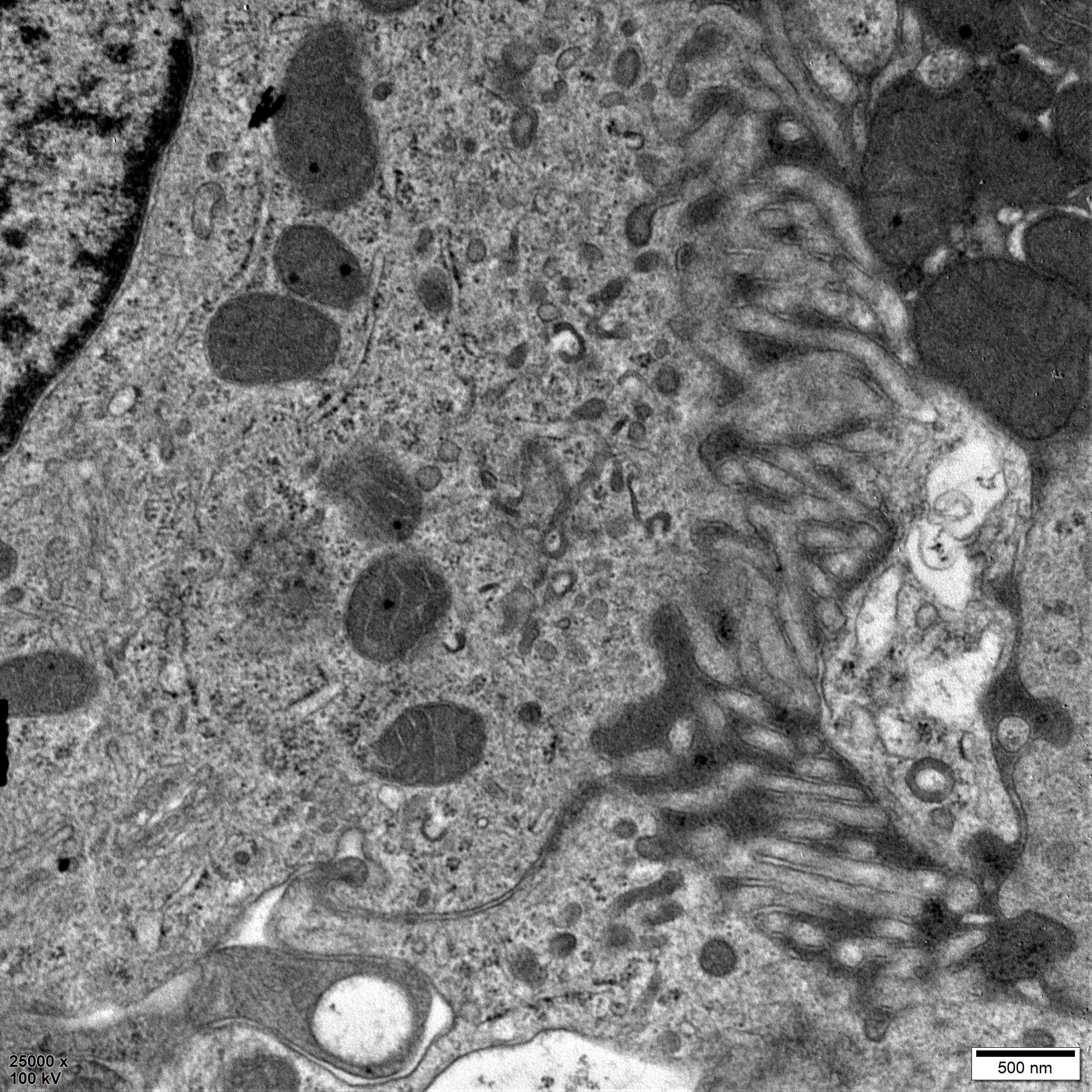

Supplement: Supplementary file 6 — Source data Fig. 1 [file 44321_2024_81_MOESM6_ESM.zip › Figure 1/1D/Health TEM .jpg]

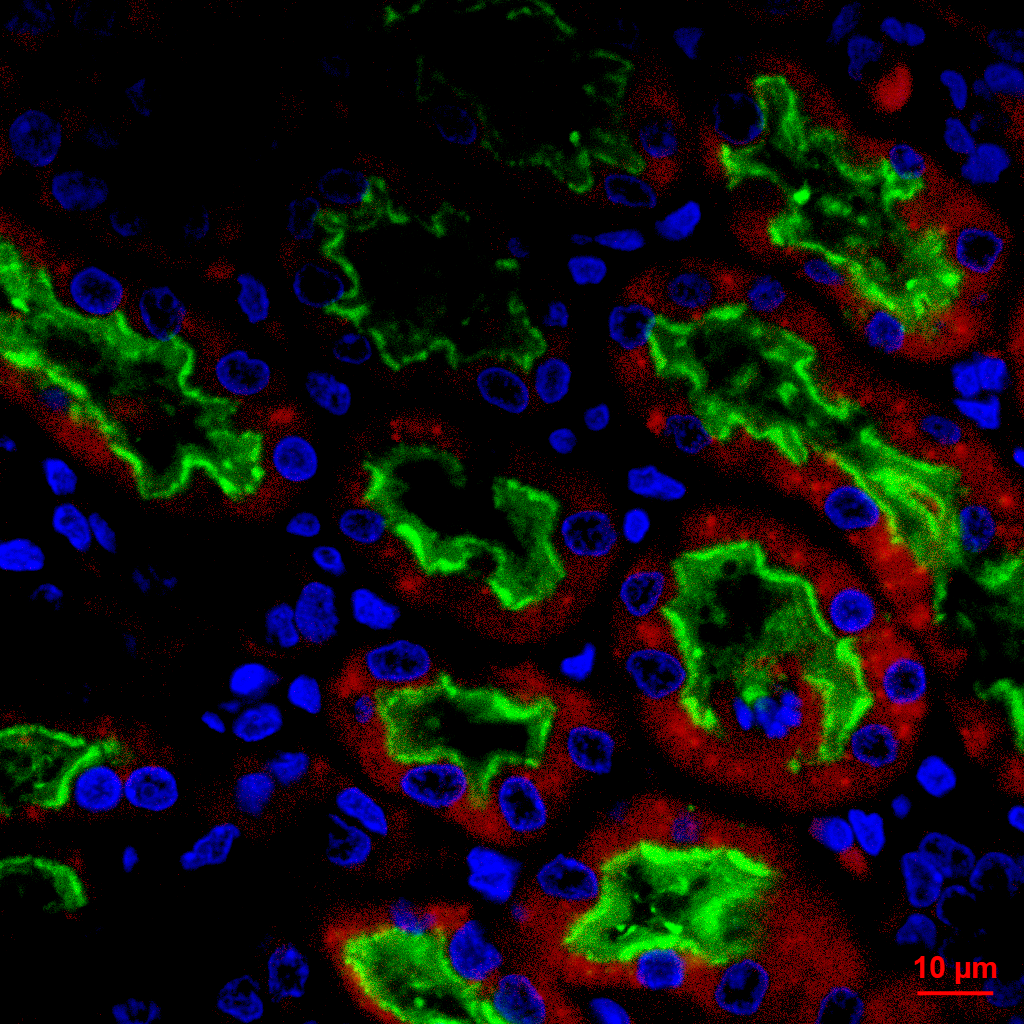

Supplement: Supplementary file 6 — Source data Fig. 1 [file 44321_2024_81_MOESM6_ESM.zip › Figure 1/1K/CKD Insig1_LTL.tif]

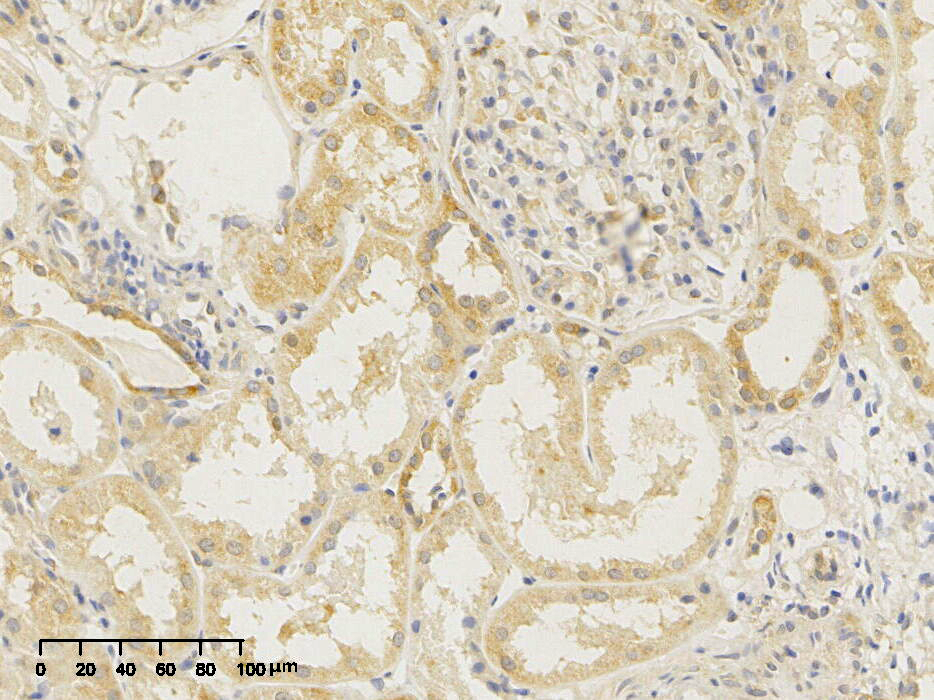

Supplement: Supplementary file 6 — Source data Fig. 1 [file 44321_2024_81_MOESM6_ESM.zip › Figure 1/1N/CKD.tif]

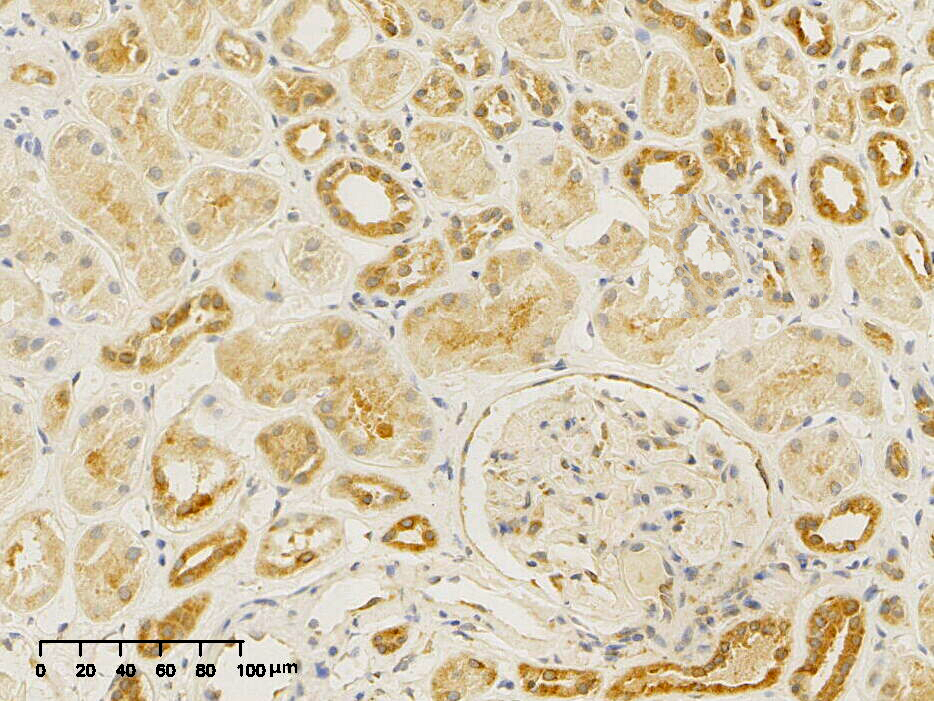

Supplement: Supplementary file 6 — Source data Fig. 1 [file 44321_2024_81_MOESM6_ESM.zip › Figure 1/1N/Normal.tif]

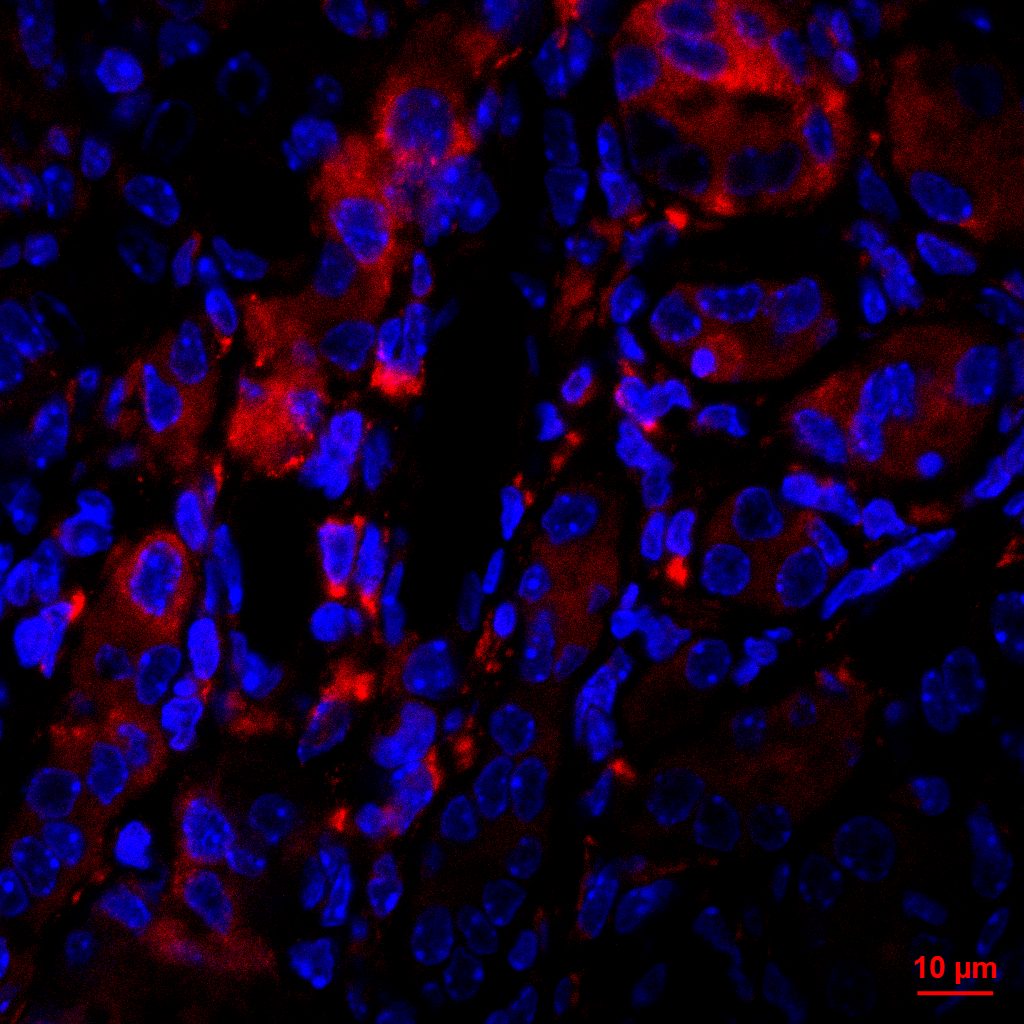

Supplement: Supplementary file 7 — Source data Fig. 2 [file 44321_2024_81_MOESM7_ESM.zip › Figure 2/2I/Insig1∩üäKap+ UUO IF.tif]

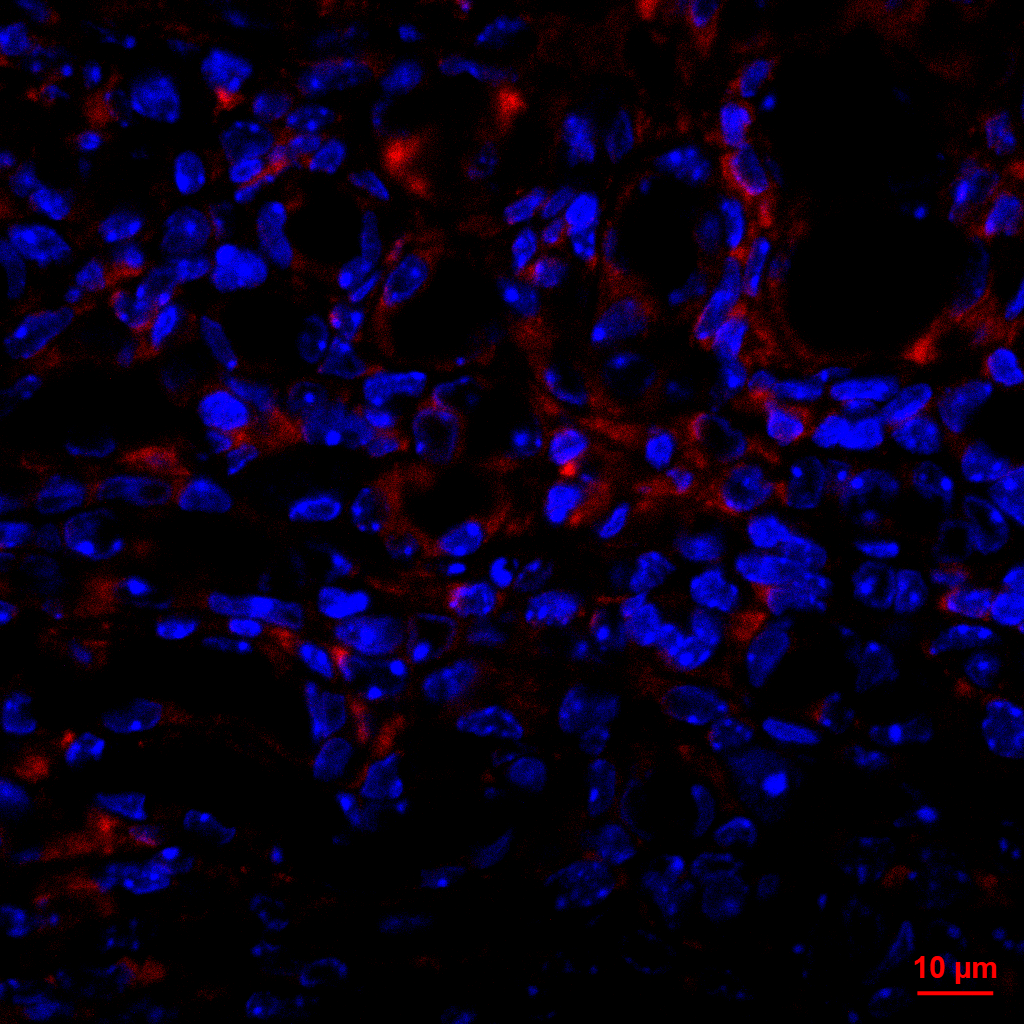

Supplement: Supplementary file 7 — Source data Fig. 2 [file 44321_2024_81_MOESM7_ESM.zip › Figure 2/2I/Insig1floxflox+ UUO IF.tif]

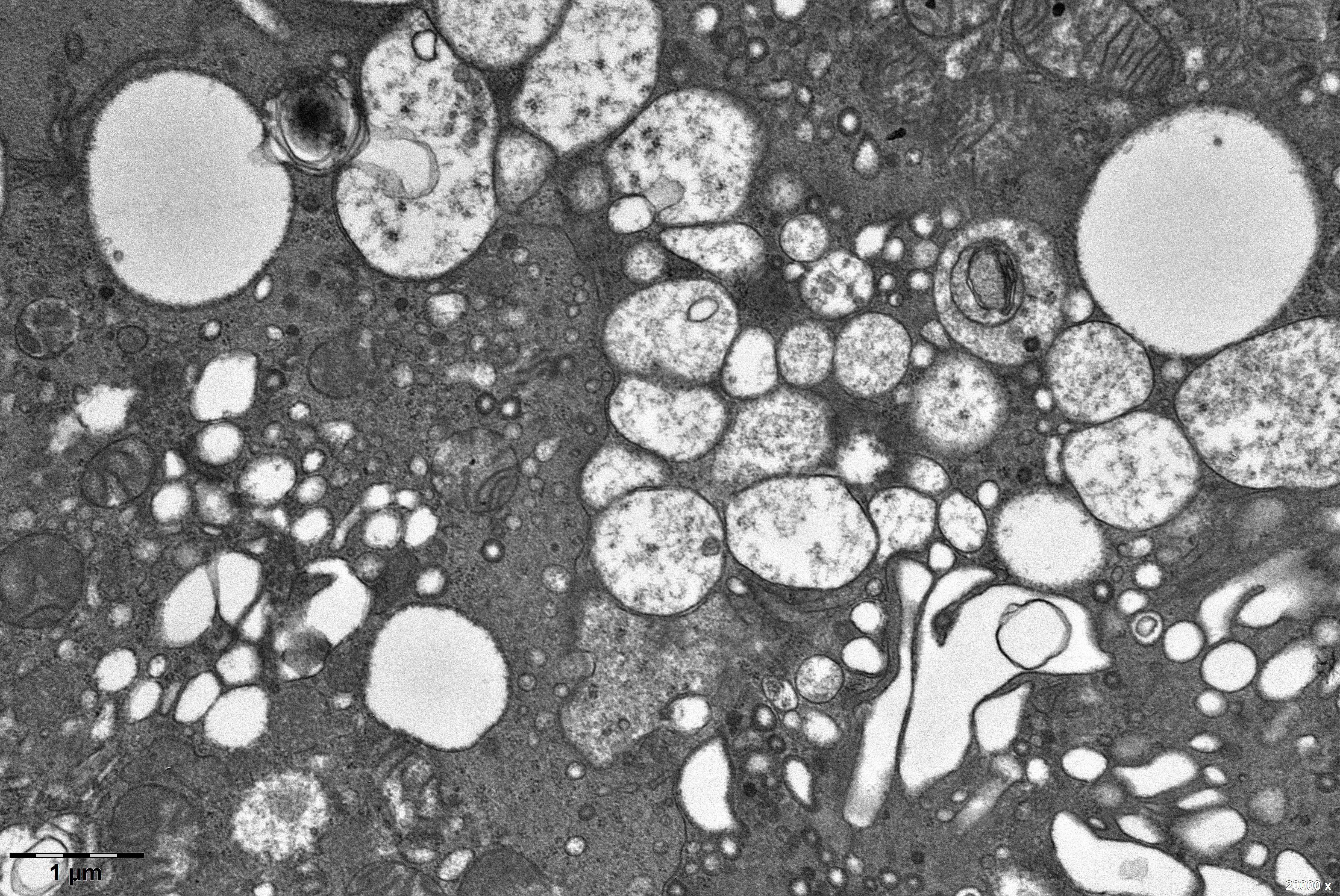

Supplement: Supplementary file 7 — Source data Fig. 2 [file 44321_2024_81_MOESM7_ESM.zip › Figure 2/2H/Insig1∩üäKap+ UUO TEM.tif]

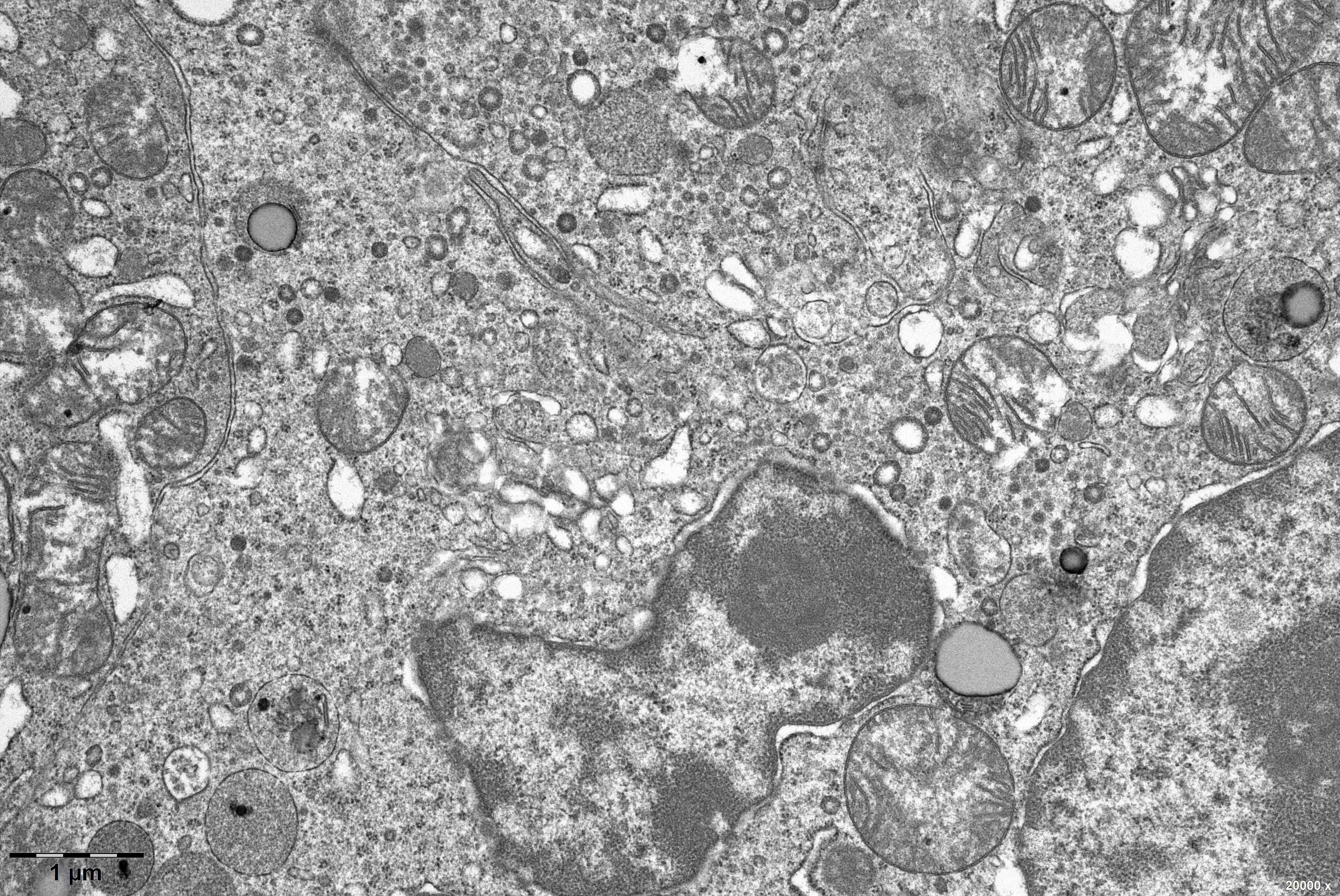

Supplement: Supplementary file 7 — Source data Fig. 2 [file 44321_2024_81_MOESM7_ESM.zip › Figure 2/2H/Insig1floxflox+ UUO TEM.tif]

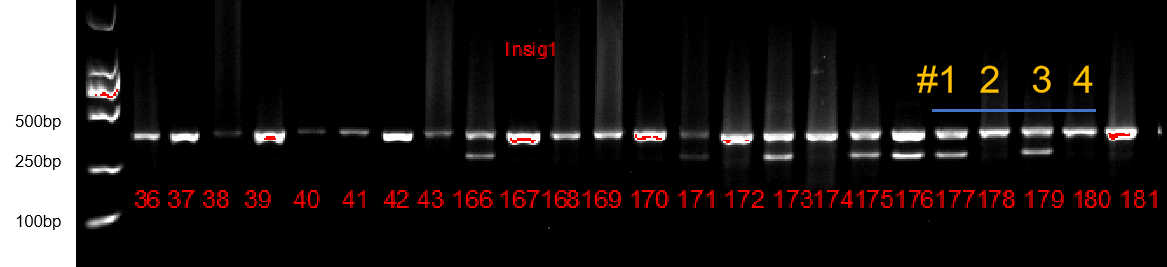

Supplement: Supplementary file 7 — Source data Fig. 2 [file 44321_2024_81_MOESM7_ESM.zip › Figure 2/2A/Gel Insig1floxflox.tif]

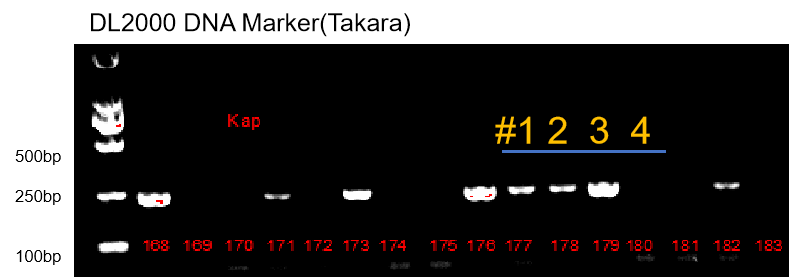

Supplement: Supplementary file 7 — Source data Fig. 2 [file 44321_2024_81_MOESM7_ESM.zip › Figure 2/2A/Gel Kap-Cre.tif]

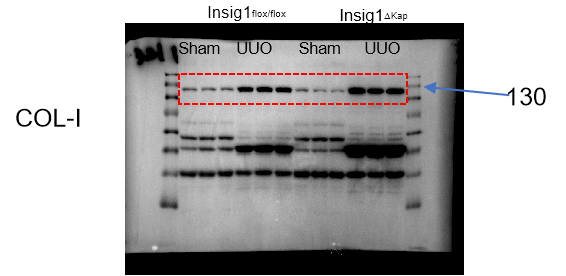

Supplement: Supplementary file 7 — Source data Fig. 2 [file 44321_2024_81_MOESM7_ESM.zip › Figure 2/2F/western COL-I.tif]

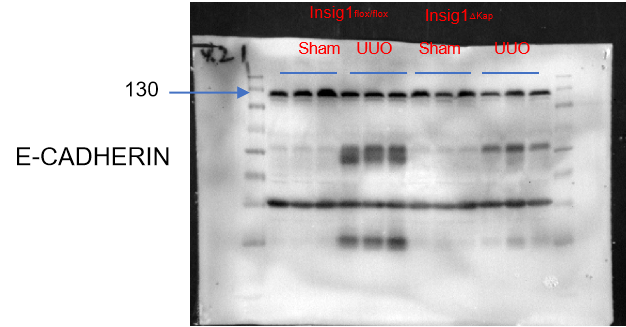

Supplement: Supplementary file 7 — Source data Fig. 2 [file 44321_2024_81_MOESM7_ESM.zip › Figure 2/2F/western E-CADHERIN.tif]

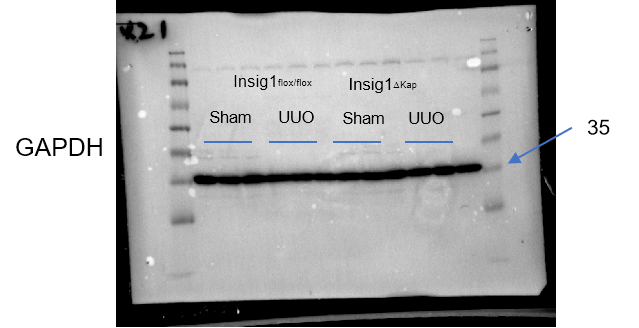

Supplement: Supplementary file 7 — Source data Fig. 2 [file 44321_2024_81_MOESM7_ESM.zip › Figure 2/2F/western GAPDH(E-CADHERIN).tif]

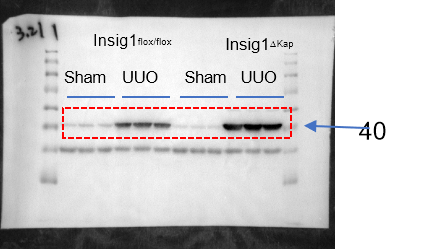

Supplement: Supplementary file 7 — Source data Fig. 2 [file 44321_2024_81_MOESM7_ESM.zip › Figure 2/2F/western ╬▒-SMA.tif]

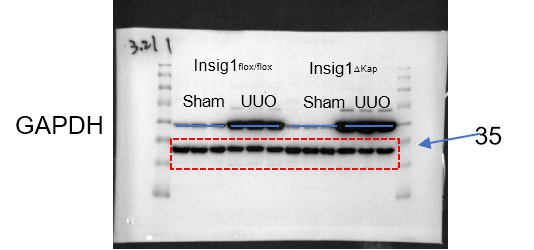

Supplement: Supplementary file 7 — Source data Fig. 2 [file 44321_2024_81_MOESM7_ESM.zip › Figure 2/2F/western GAPDH(COL-I,╬▒-SMA, VIM).tif]

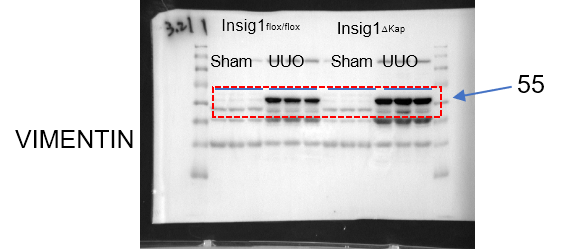

Supplement: Supplementary file 7 — Source data Fig. 2 [file 44321_2024_81_MOESM7_ESM.zip › Figure 2/2F/western VIMENTIN.tif]

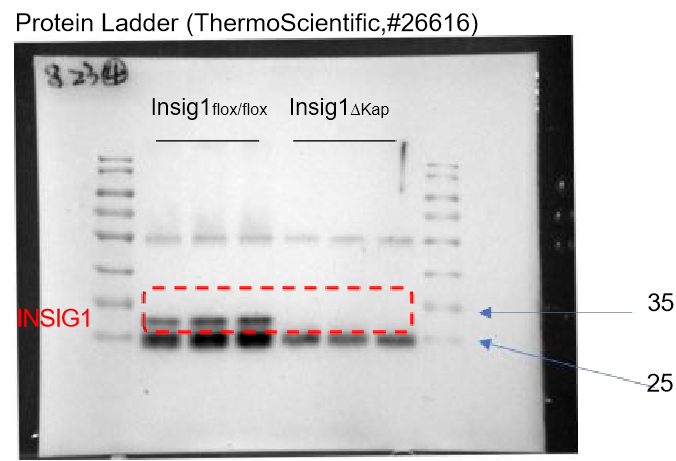

Supplement: Supplementary file 7 — Source data Fig. 2 [file 44321_2024_81_MOESM7_ESM.zip › Figure 2/2B/western INSIG1.tif]

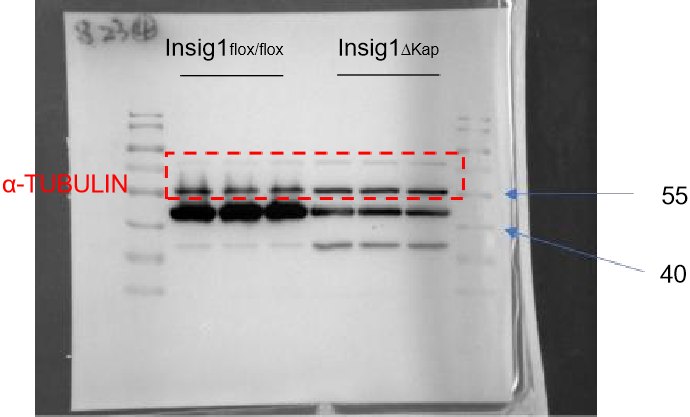

Supplement: Supplementary file 7 — Source data Fig. 2 [file 44321_2024_81_MOESM7_ESM.zip › Figure 2/2B/western ╬▒-TUBULIN.tif]

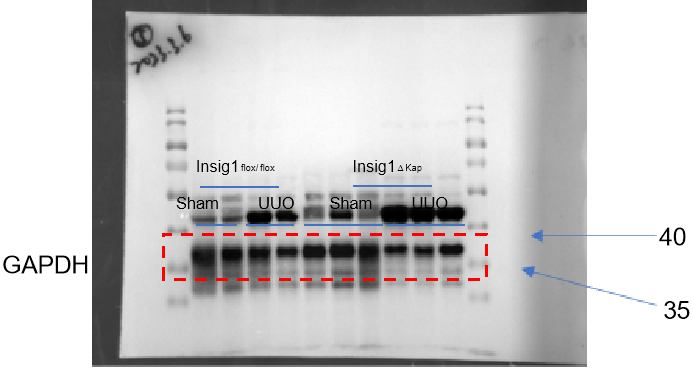

Supplement: Supplementary file 7 — Source data Fig. 2 [file 44321_2024_81_MOESM7_ESM.zip › Figure 2/2F/Repeats/western GAPDH repeat(COL-I,╬▒-SMA).tif]

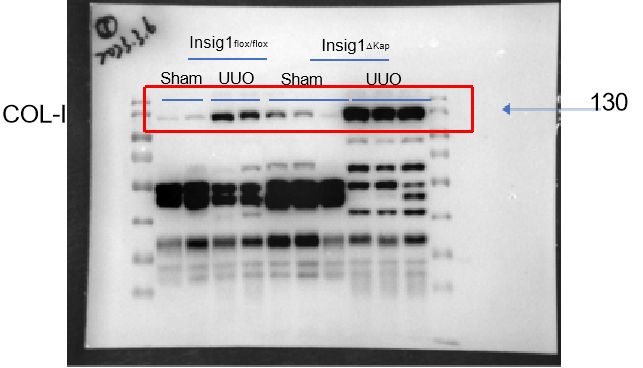

Supplement: Supplementary file 7 — Source data Fig. 2 [file 44321_2024_81_MOESM7_ESM.zip › Figure 2/2F/Repeats/western COL-I repeat.tif]

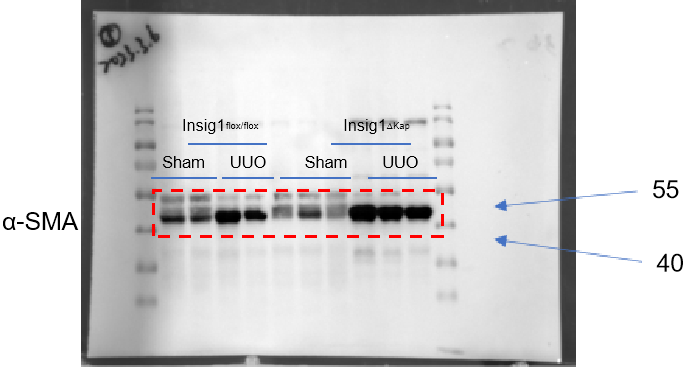

Supplement: Supplementary file 7 — Source data Fig. 2 [file 44321_2024_81_MOESM7_ESM.zip › Figure 2/2F/Repeats/western ╬▒-SMA repeat.tif]

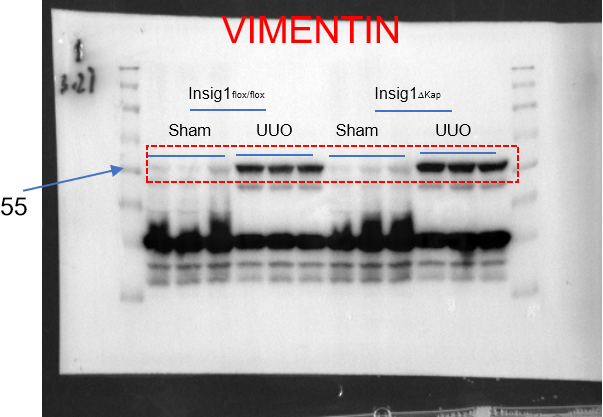

Supplement: Supplementary file 7 — Source data Fig. 2 [file 44321_2024_81_MOESM7_ESM.zip › Figure 2/2F/Repeats/western VIMENTIN repeat.tif]

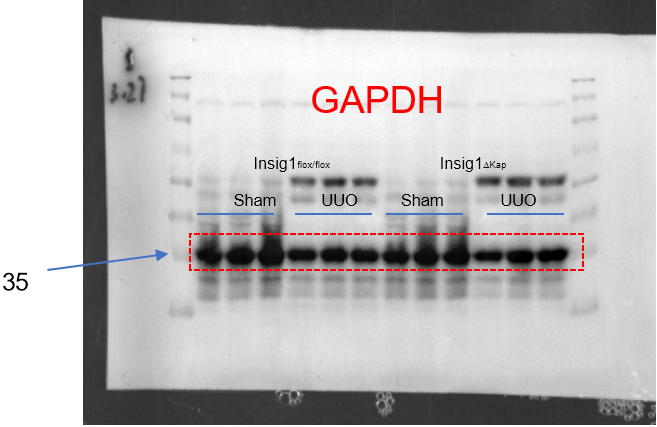

Supplement: Supplementary file 7 — Source data Fig. 2 [file 44321_2024_81_MOESM7_ESM.zip › Figure 2/2F/Repeats/western GAPDH repeat(E-CAD,VIM).tif]

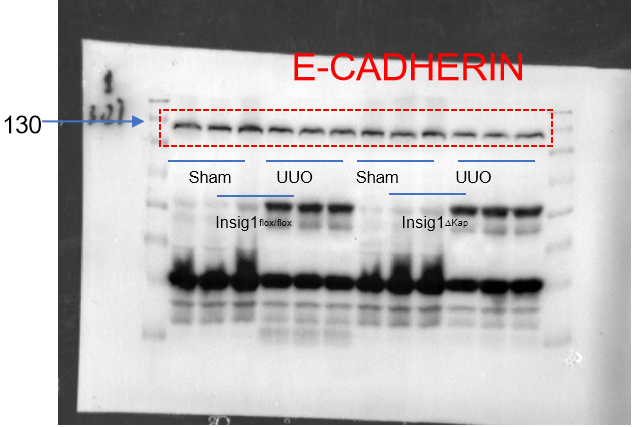

Supplement: Supplementary file 7 — Source data Fig. 2 [file 44321_2024_81_MOESM7_ESM.zip › Figure 2/2F/Repeats/western E-CADHERIN repeat.tif]

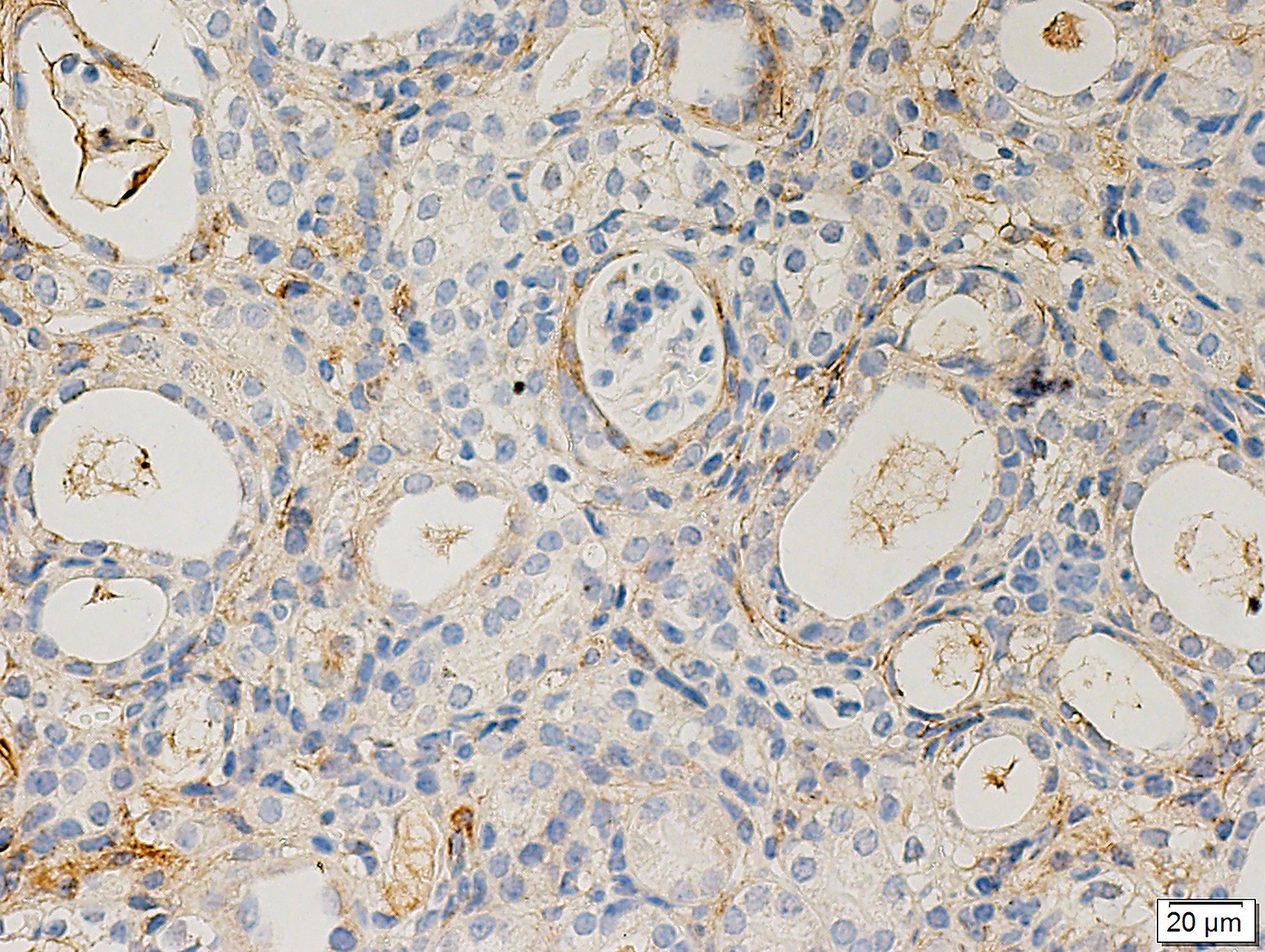

Supplement: Supplementary file 7 — Source data Fig. 2 [file 44321_2024_81_MOESM7_ESM.zip › Figure 2/2D/FN IHC/Insig1floxflox+ UUO.tif]

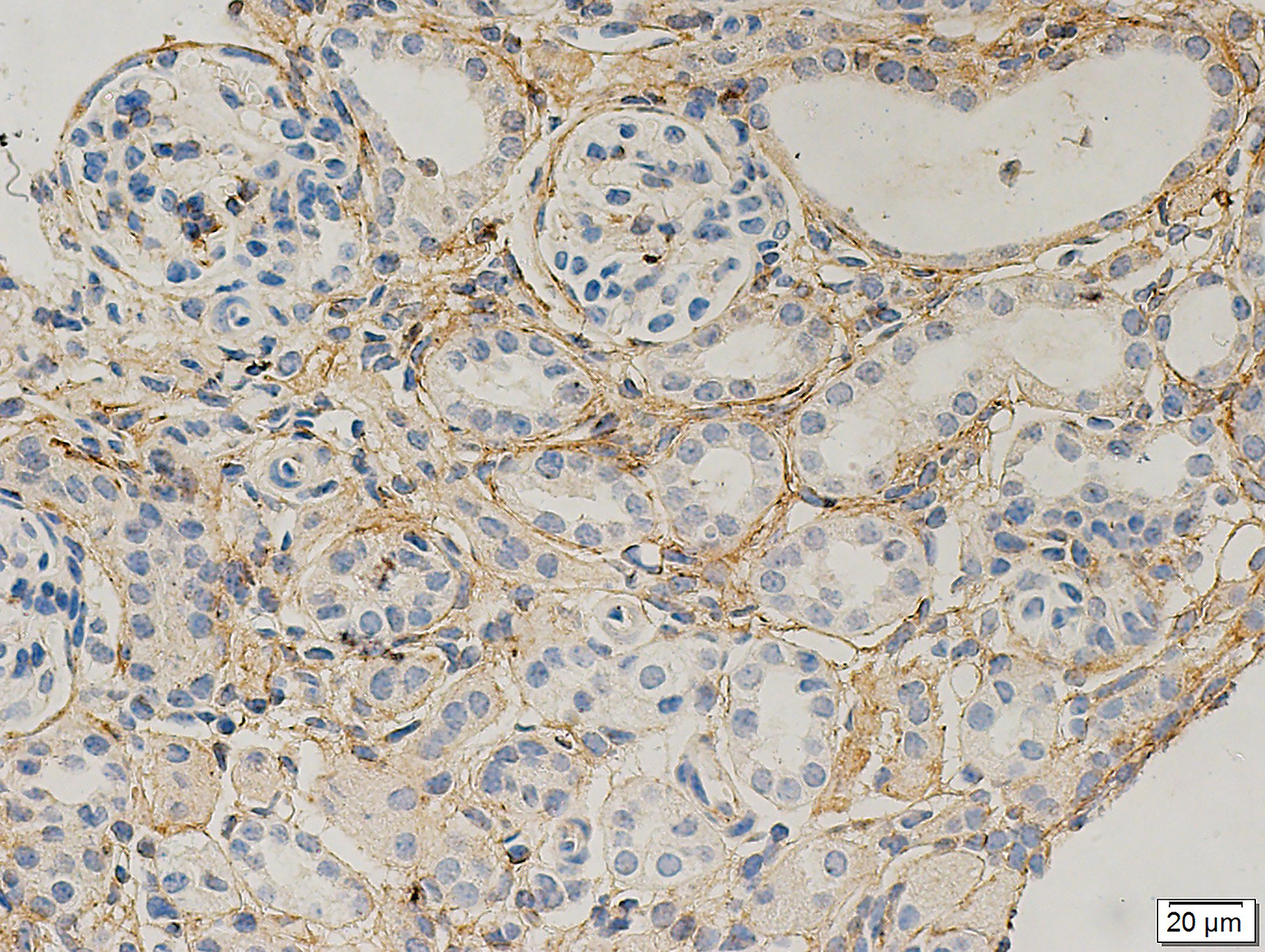

Supplement: Supplementary file 7 — Source data Fig. 2 [file 44321_2024_81_MOESM7_ESM.zip › Figure 2/2D/FN IHC/Insig1∩üäKap+ UUO.tif]

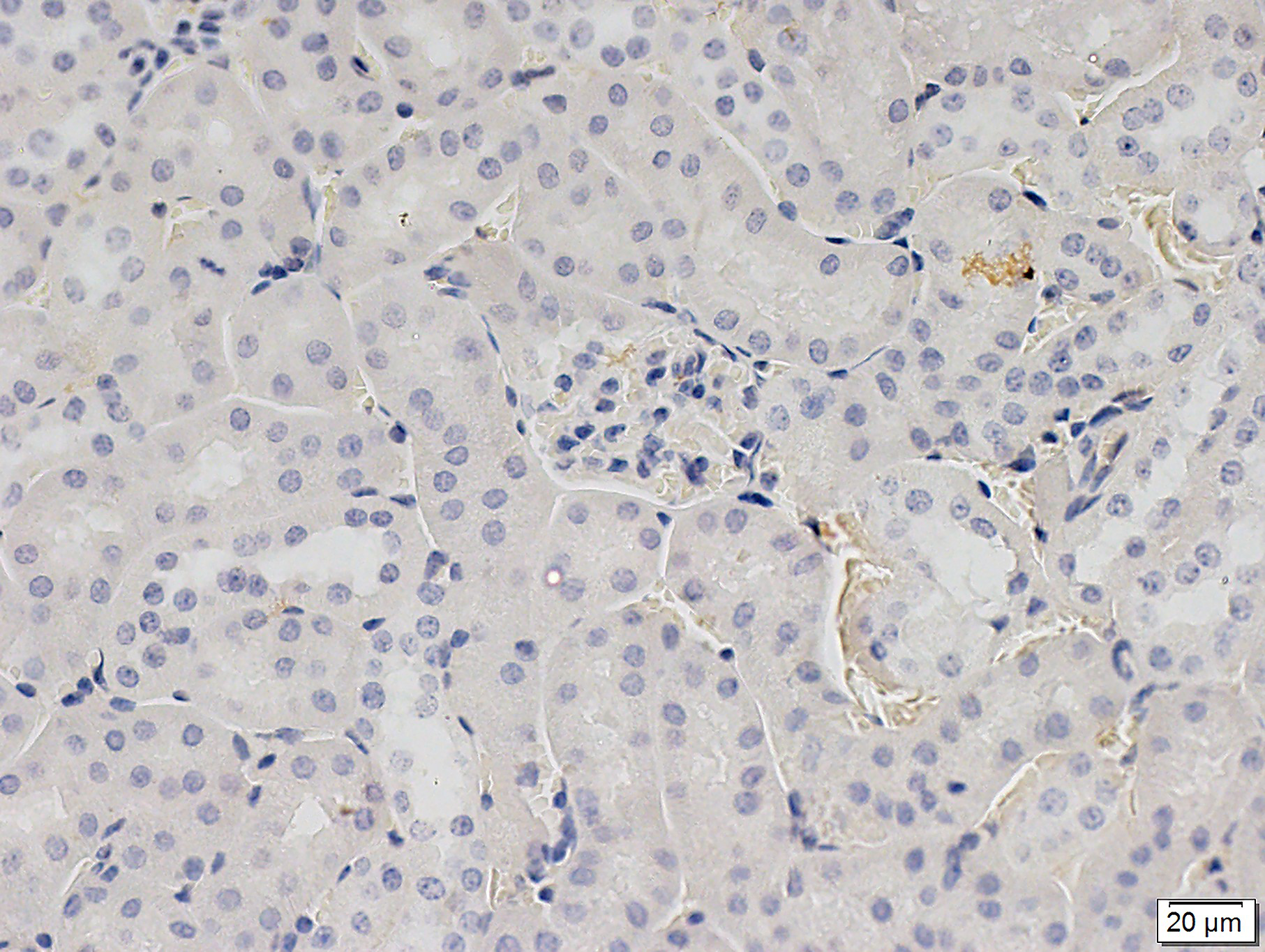

Supplement: Supplementary file 7 — Source data Fig. 2 [file 44321_2024_81_MOESM7_ESM.zip › Figure 2/2D/FN IHC/Insig1floxflox.tif]

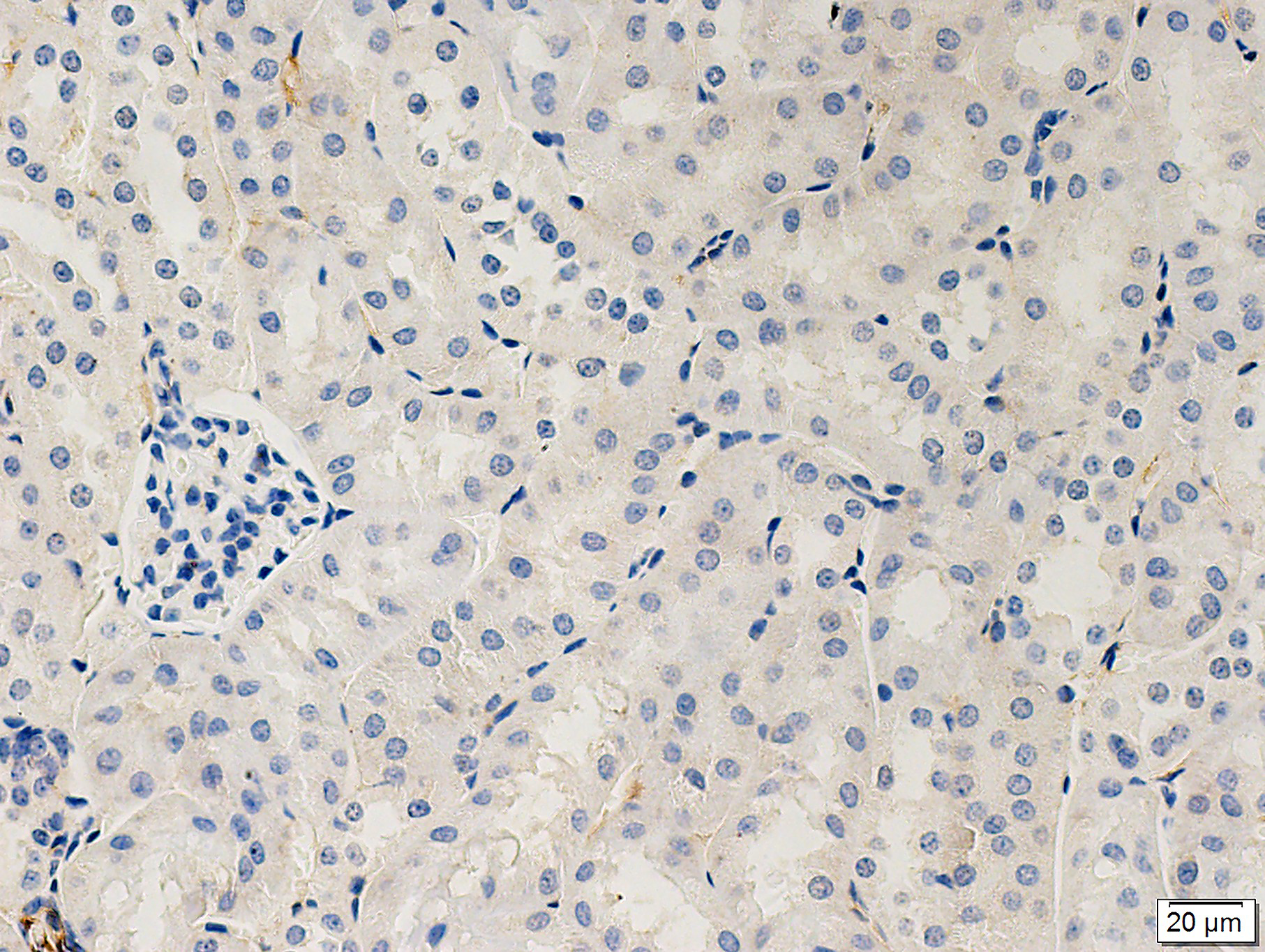

Supplement: Supplementary file 7 — Source data Fig. 2 [file 44321_2024_81_MOESM7_ESM.zip › Figure 2/2D/FN IHC/Insig1∩üäKap.tif]

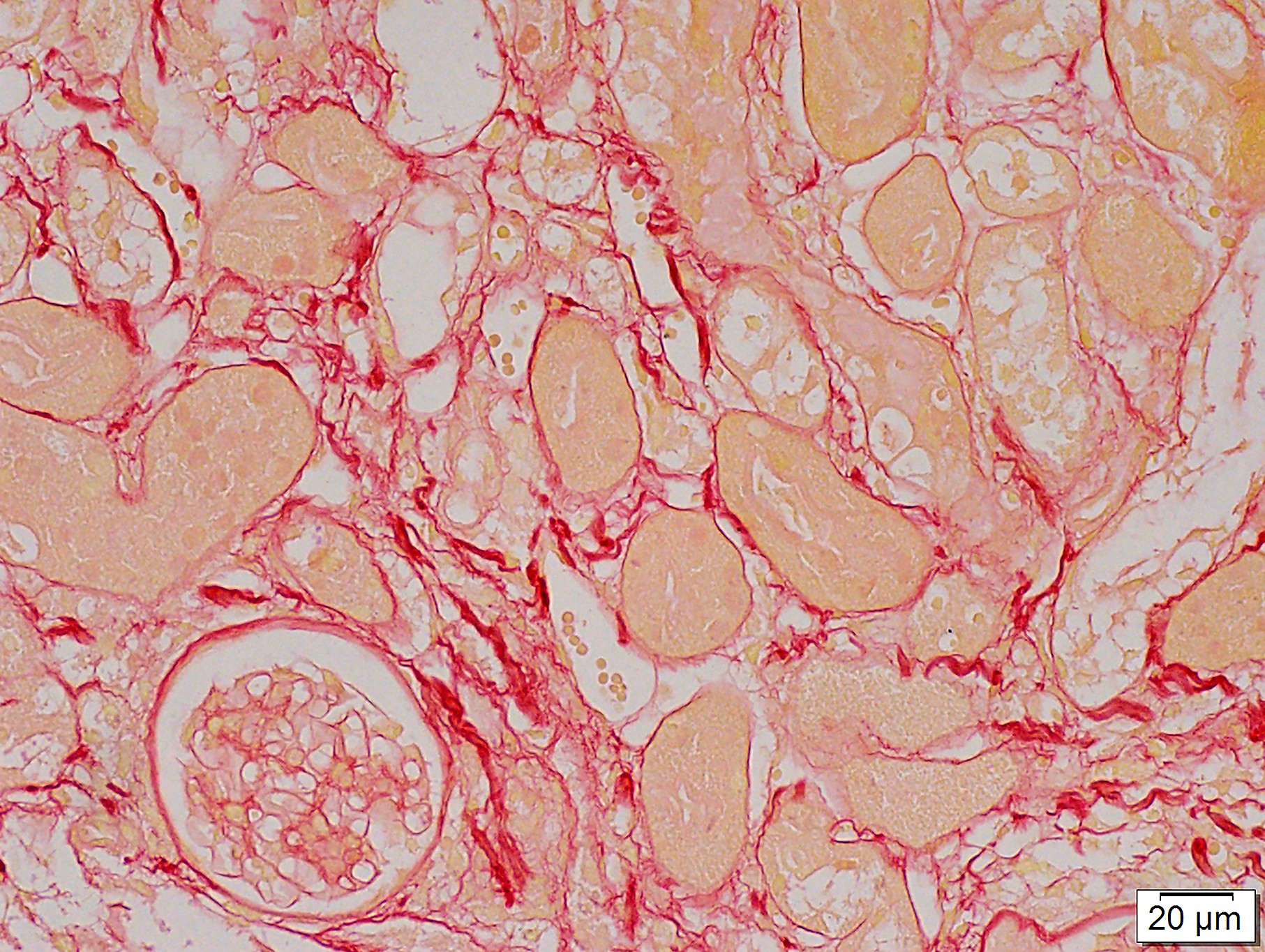

Supplement: Supplementary file 7 — Source data Fig. 2 [file 44321_2024_81_MOESM7_ESM.zip › Figure 2/2D/Sirius red/Insig1floxflox+UUO.tif]

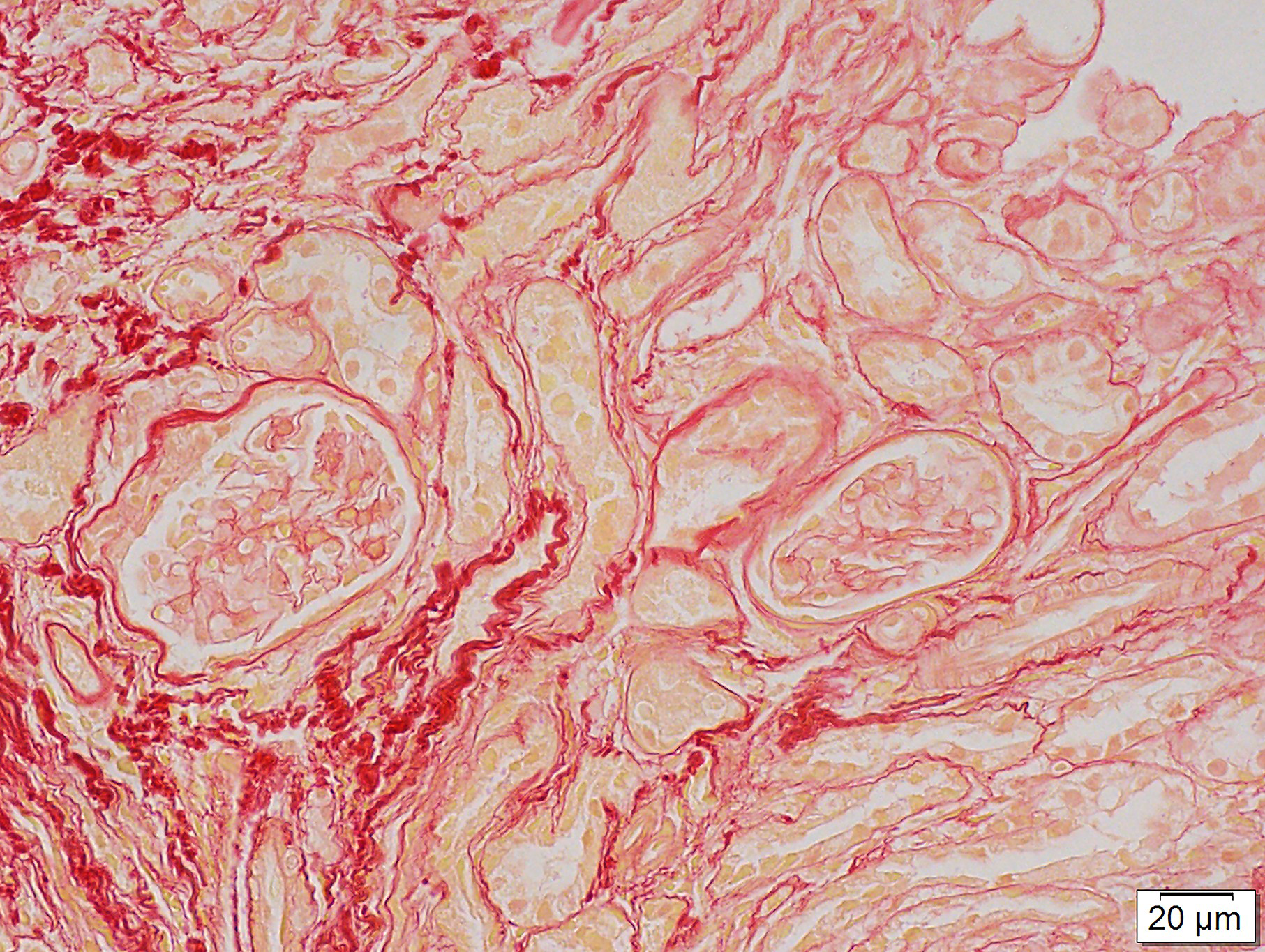

Supplement: Supplementary file 7 — Source data Fig. 2 [file 44321_2024_81_MOESM7_ESM.zip › Figure 2/2D/Sirius red/Insig1∩üäKap+ UUO.tif]

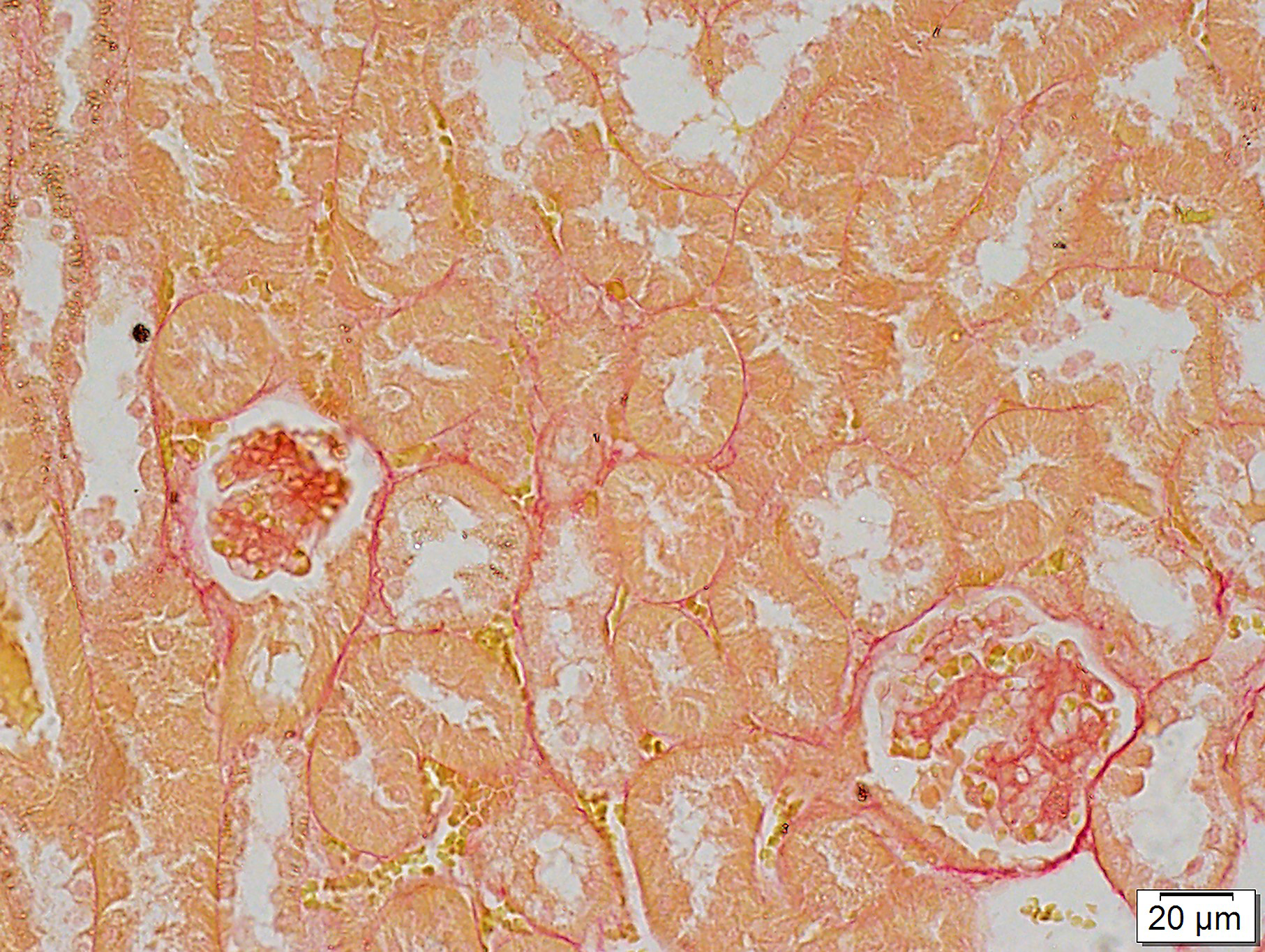

Supplement: Supplementary file 7 — Source data Fig. 2 [file 44321_2024_81_MOESM7_ESM.zip › Figure 2/2D/Sirius red/Insig1floxflox.tif]

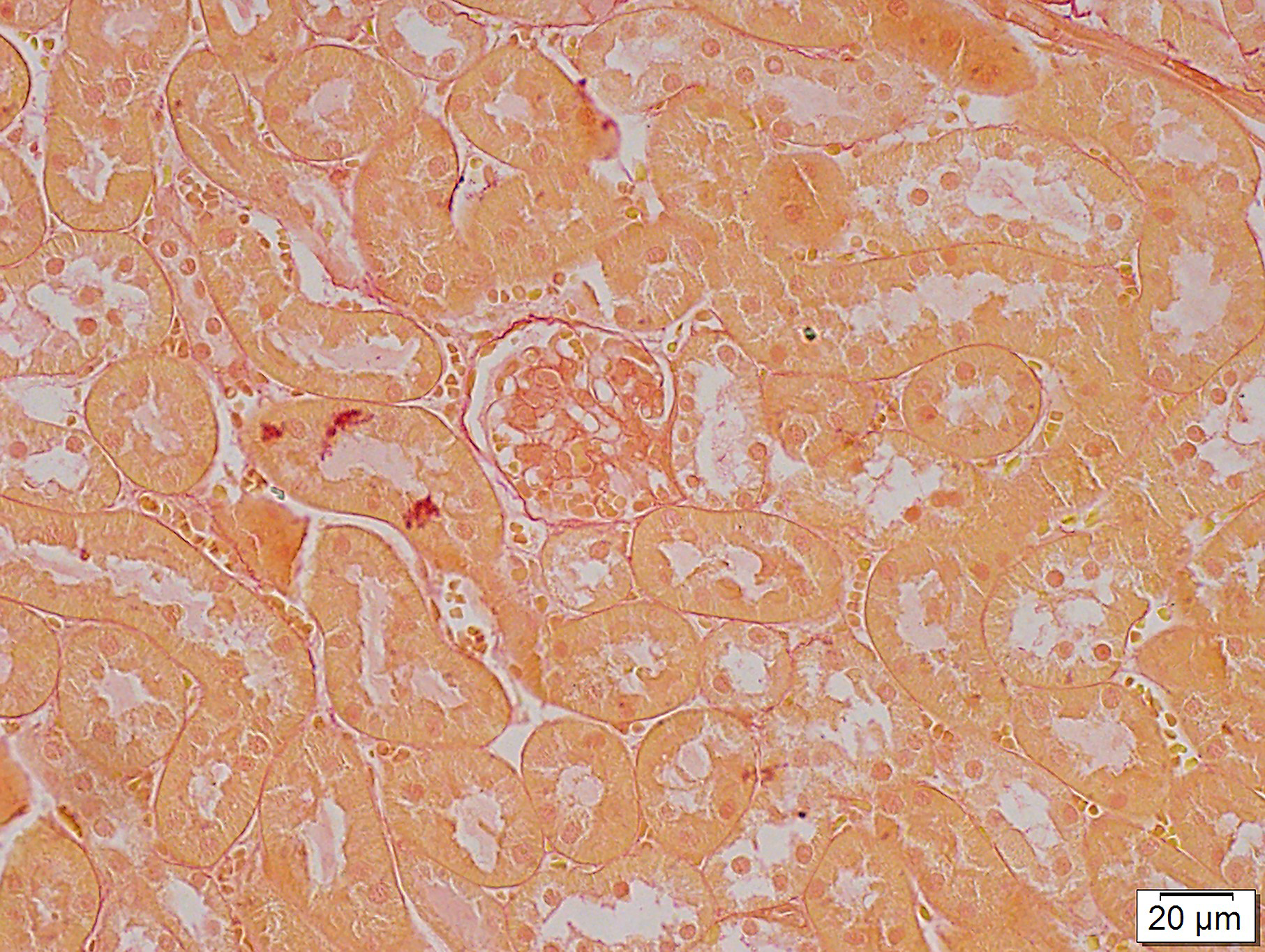

Supplement: Supplementary file 7 — Source data Fig. 2 [file 44321_2024_81_MOESM7_ESM.zip › Figure 2/2D/Sirius red/Insig1∩üäKap.tif]

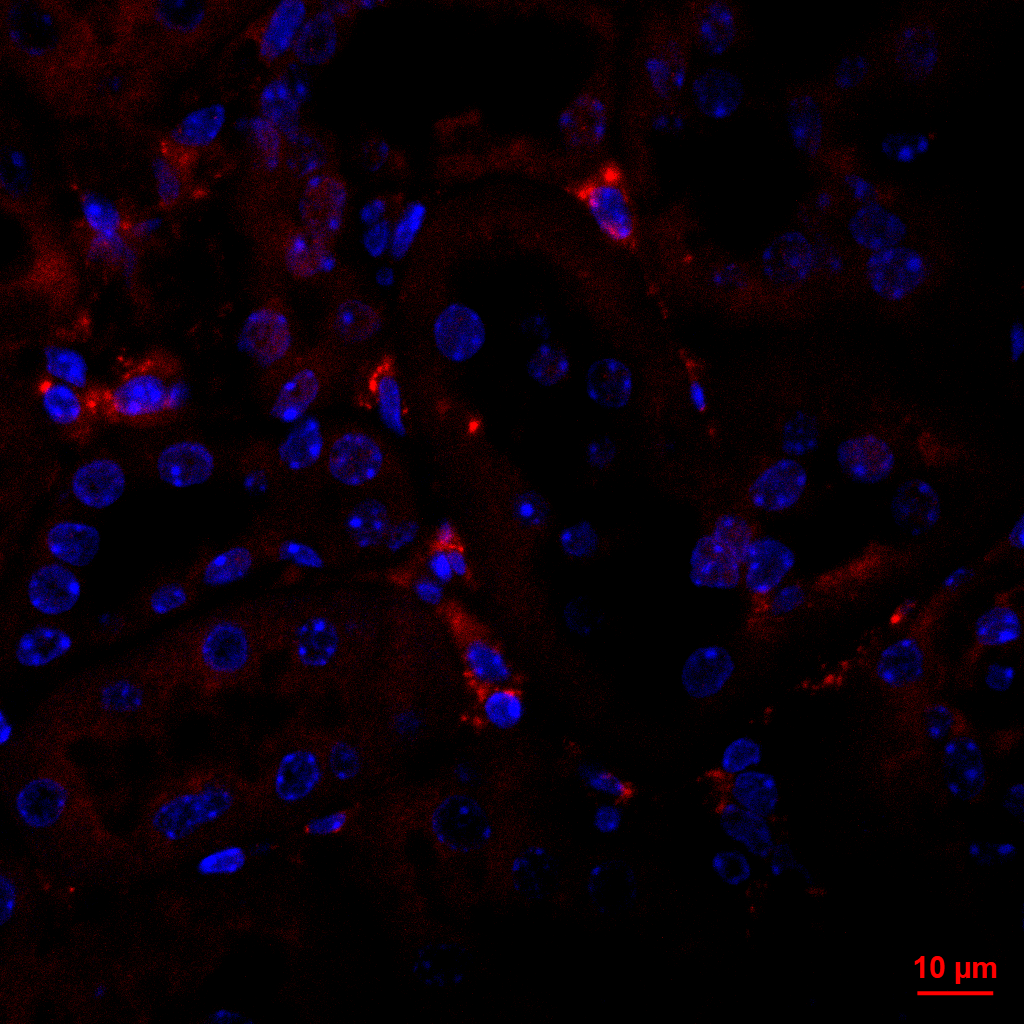

Supplement: Supplementary file 8 — Source data Fig. 3 [file 44321_2024_81_MOESM8_ESM.zip › Figure 3/3H/IK-56Nx.tif]

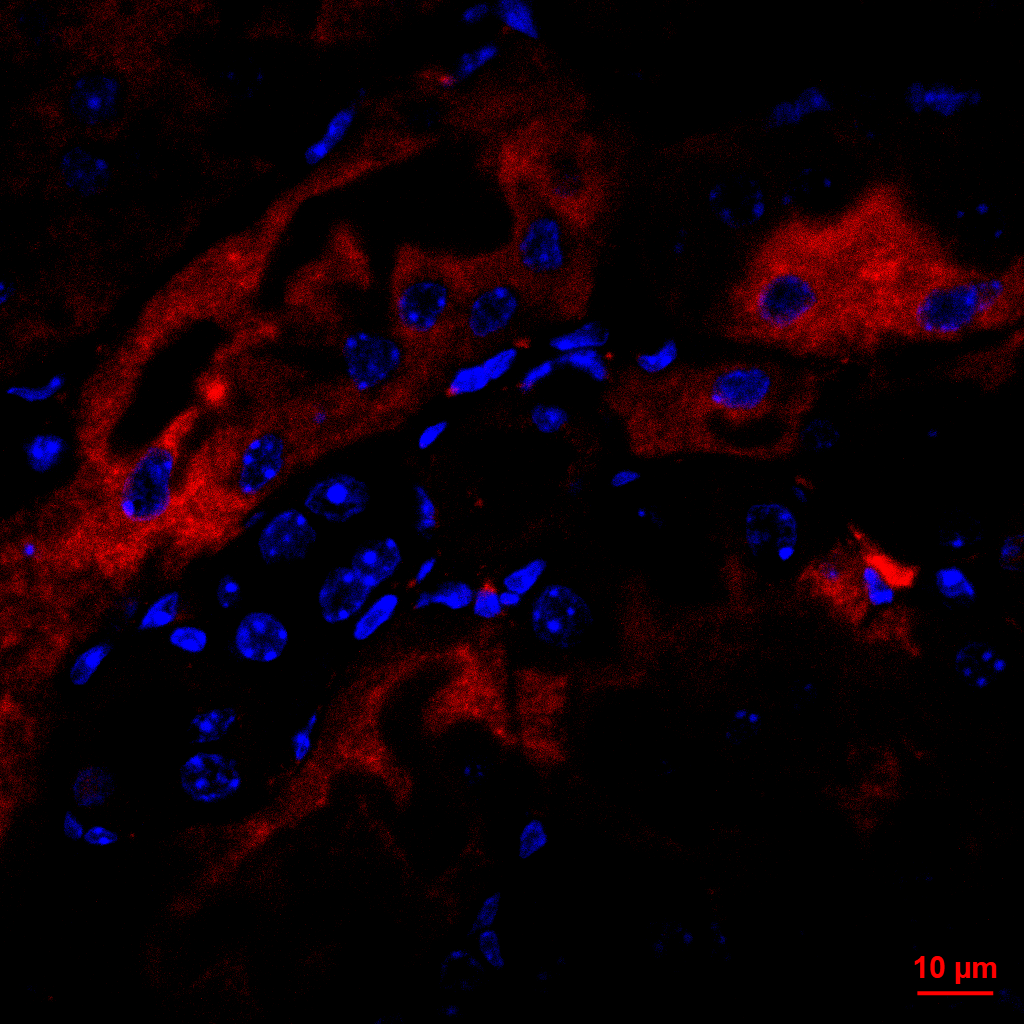

Supplement: Supplementary file 8 — Source data Fig. 3 [file 44321_2024_81_MOESM8_ESM.zip › Figure 3/3H/IK+ 56Nx.tif]

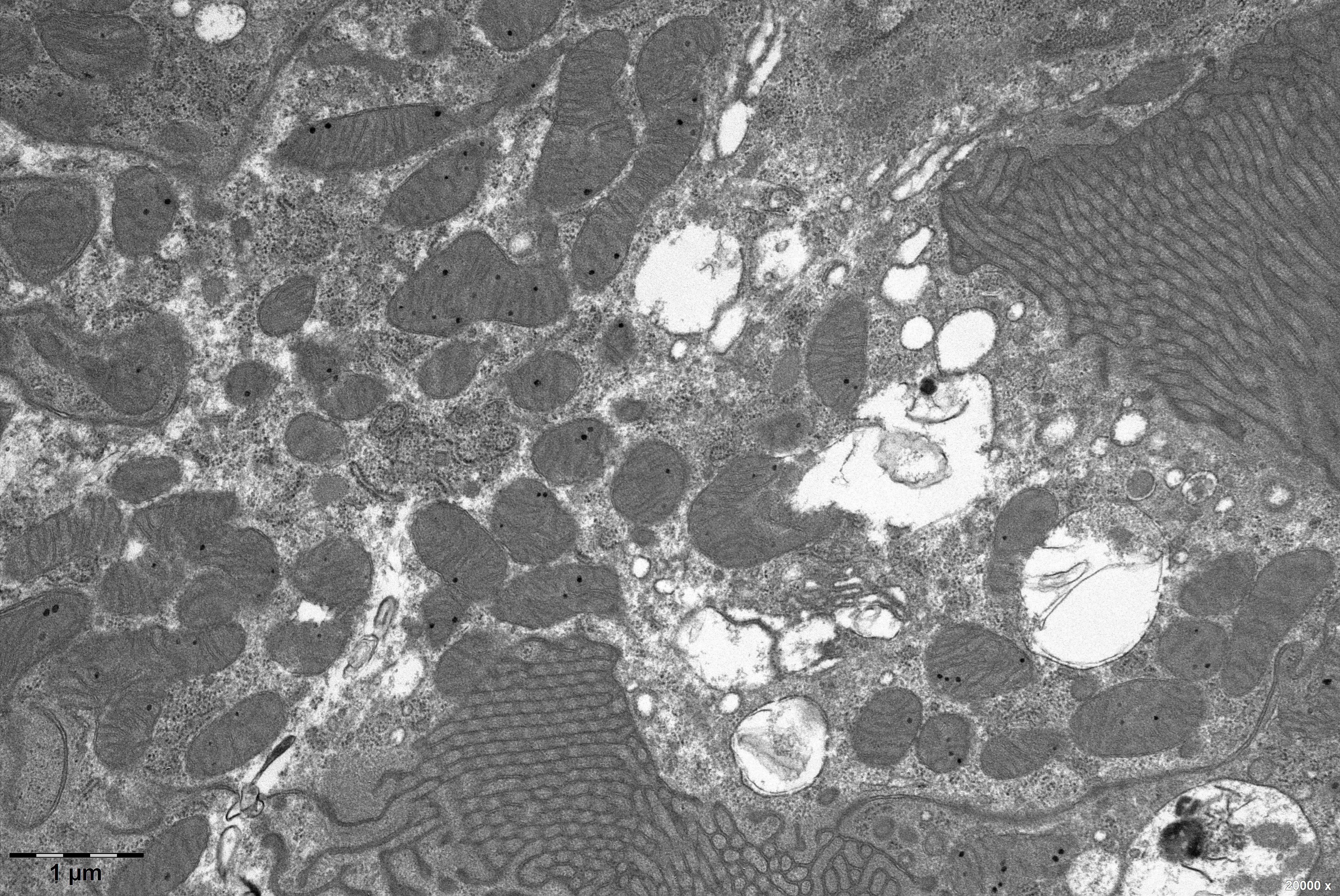

Supplement: Supplementary file 8 — Source data Fig. 3 [file 44321_2024_81_MOESM8_ESM.zip › Figure 3/3G/Insig1floxflox+56Nx.tif]

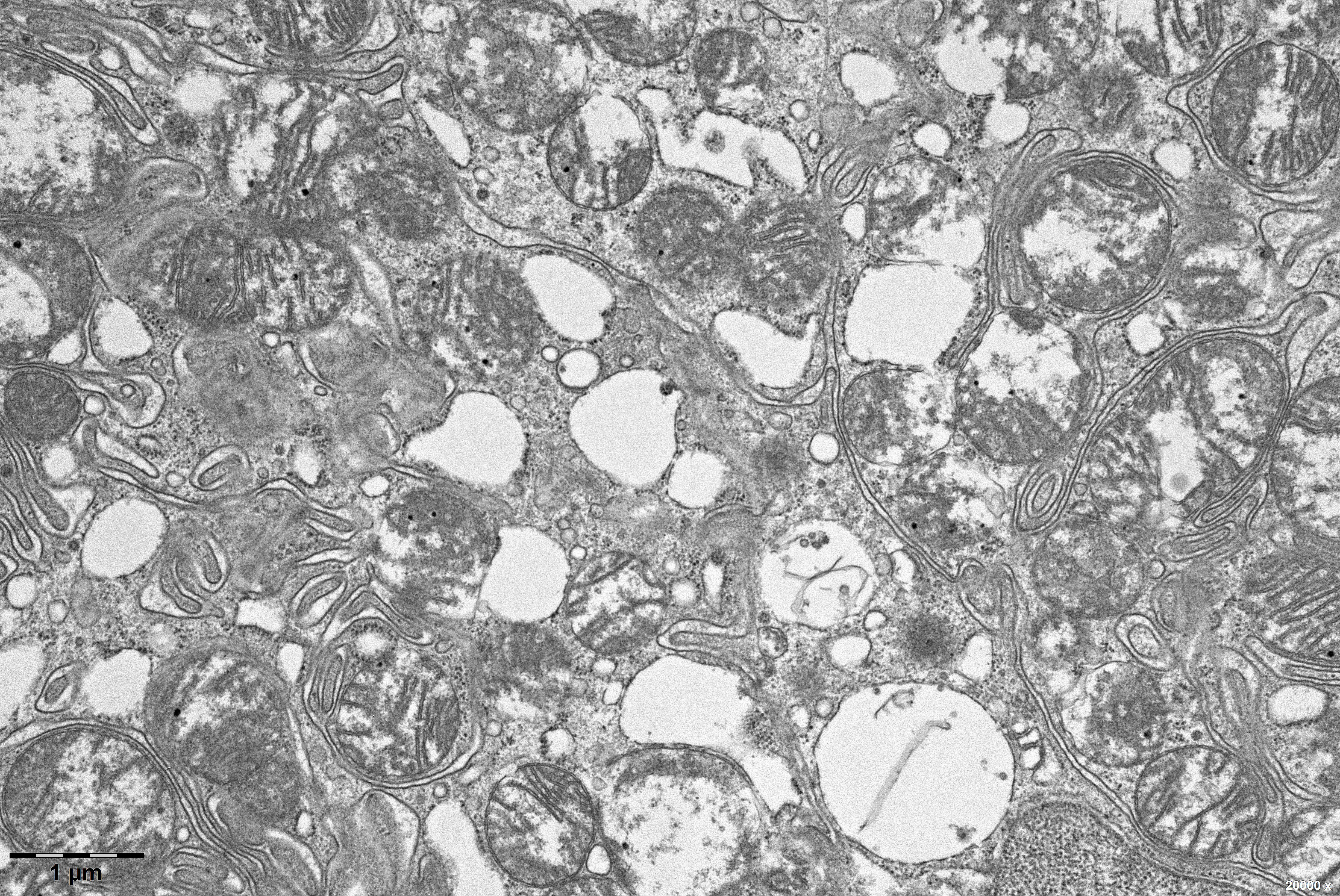

Supplement: Supplementary file 8 — Source data Fig. 3 [file 44321_2024_81_MOESM8_ESM.zip › Figure 3/3G/Insig1∩üäKap+ 56Nx.tif]

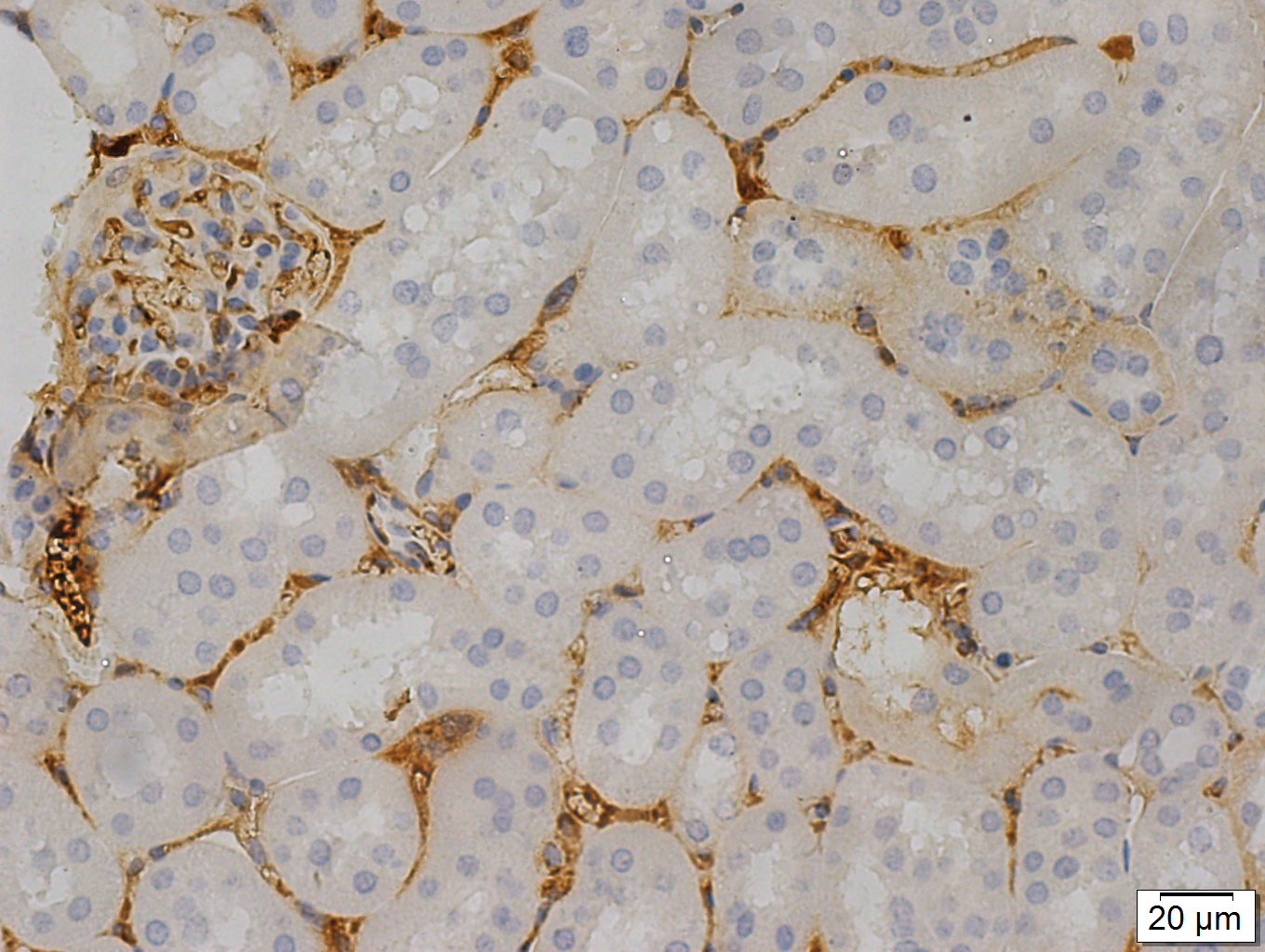

Supplement: Supplementary file 8 — Source data Fig. 3 [file 44321_2024_81_MOESM8_ESM.zip › Figure 3/3C/FN IHC/Insig1∩üäKap+56NX.tif]

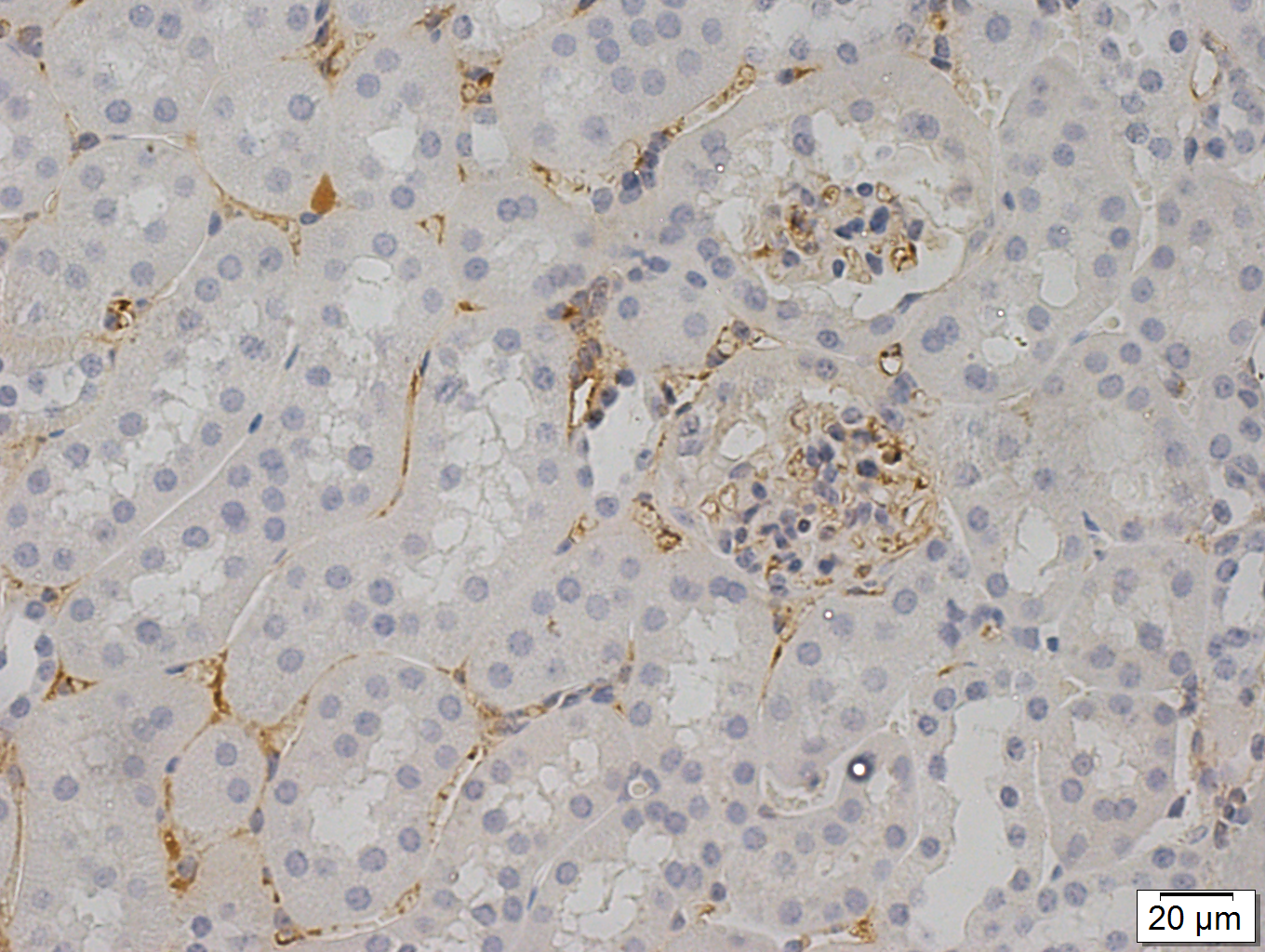

Supplement: Supplementary file 8 — Source data Fig. 3 [file 44321_2024_81_MOESM8_ESM.zip › Figure 3/3C/FN IHC/Insig1floxflox+56Nx.tif]

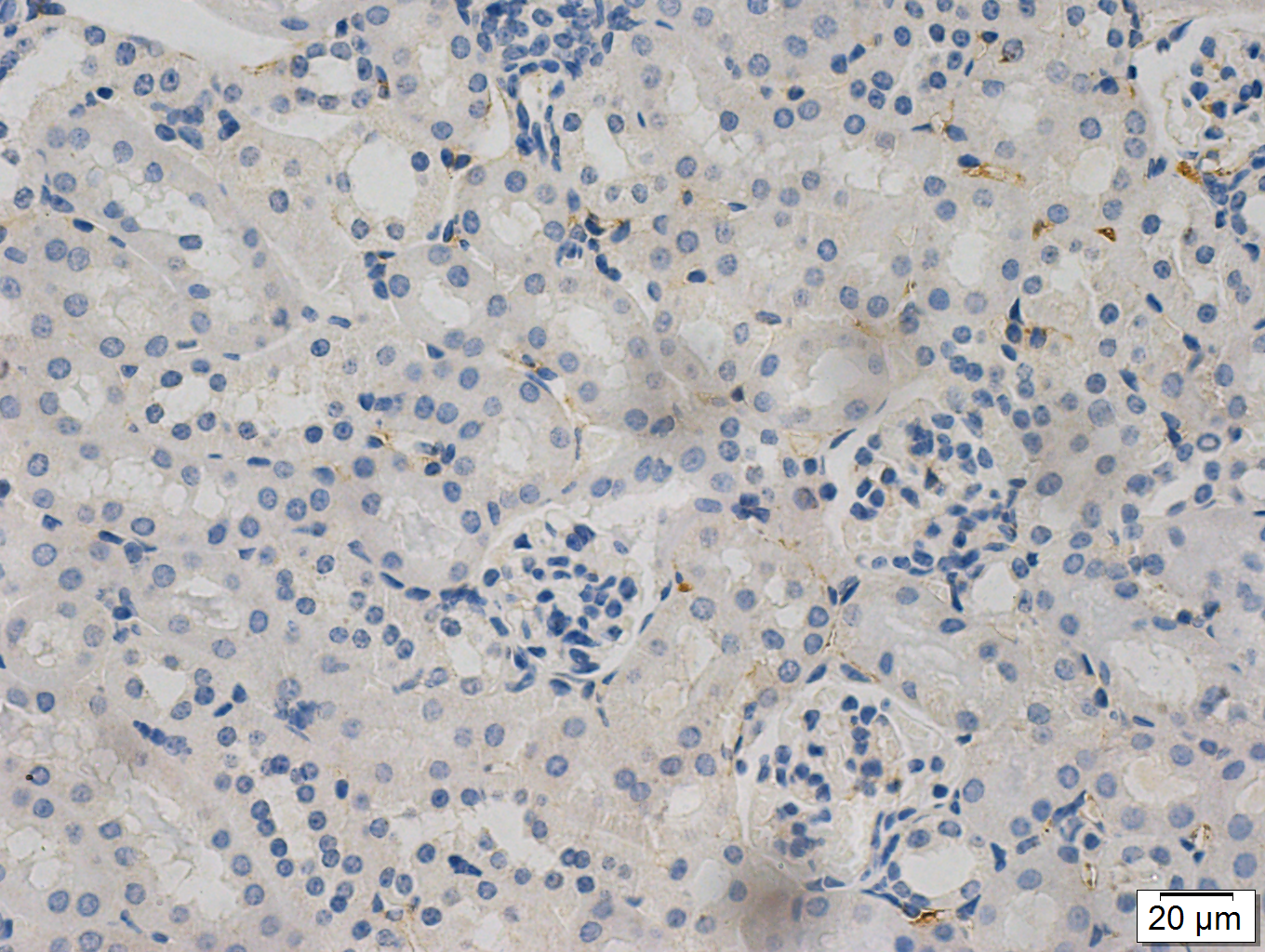

Supplement: Supplementary file 8 — Source data Fig. 3 [file 44321_2024_81_MOESM8_ESM.zip › Figure 3/3C/FN IHC/Insig1floxflox.tif]

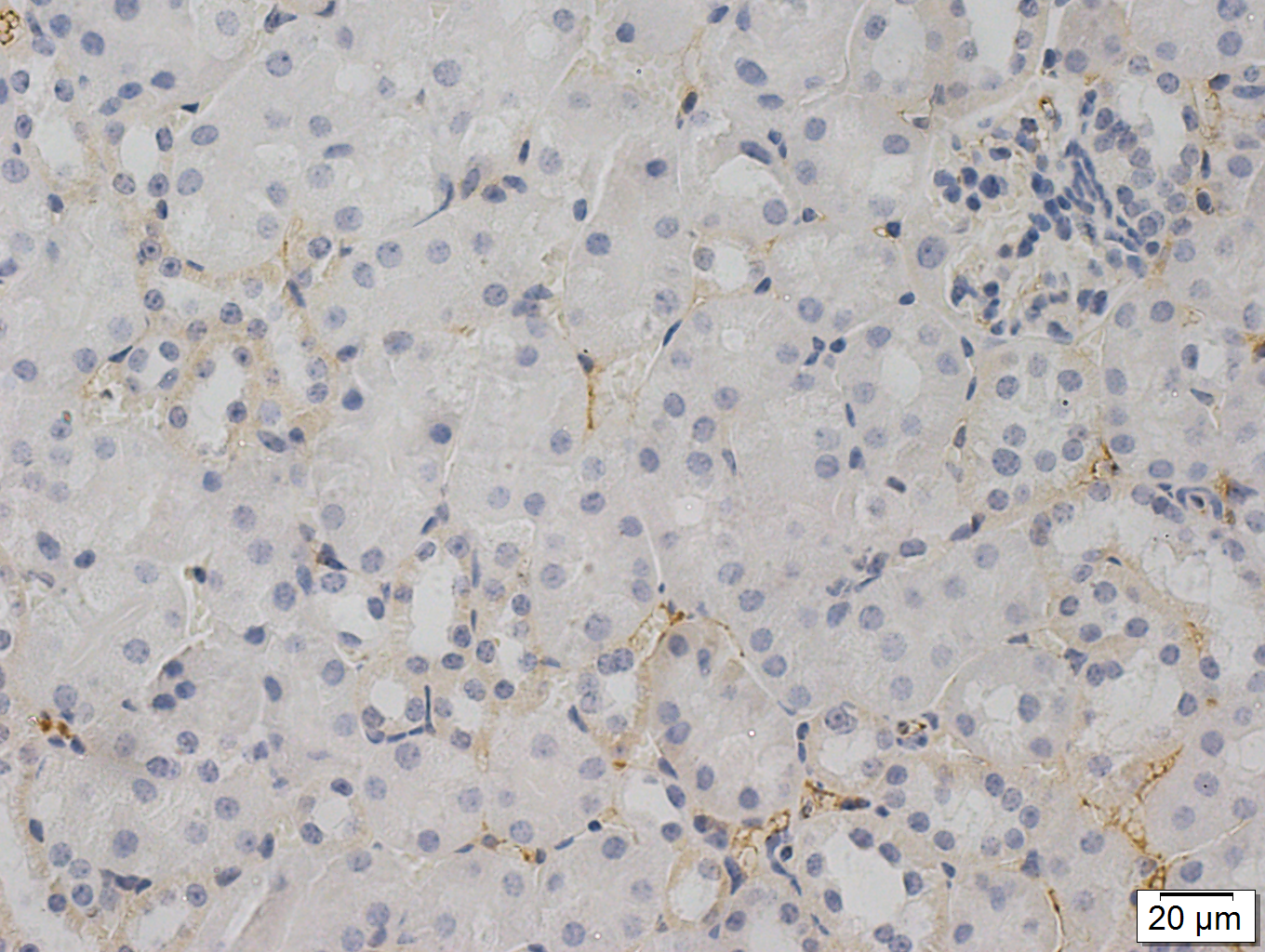

Supplement: Supplementary file 8 — Source data Fig. 3 [file 44321_2024_81_MOESM8_ESM.zip › Figure 3/3C/FN IHC/Insig1∩üäKap.tif]

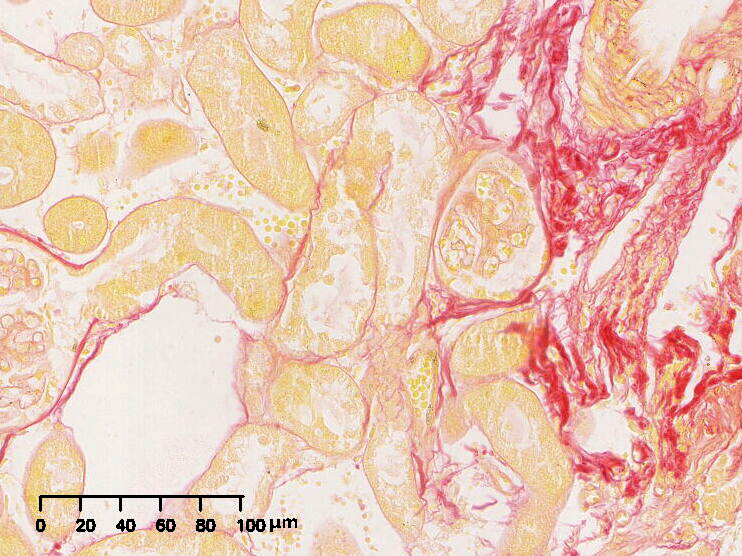

Supplement: Supplementary file 8 — Source data Fig. 3 [file 44321_2024_81_MOESM8_ESM.zip › Figure 3/3C/Sirius red/Insig1∩üäKap+56NX.tif]

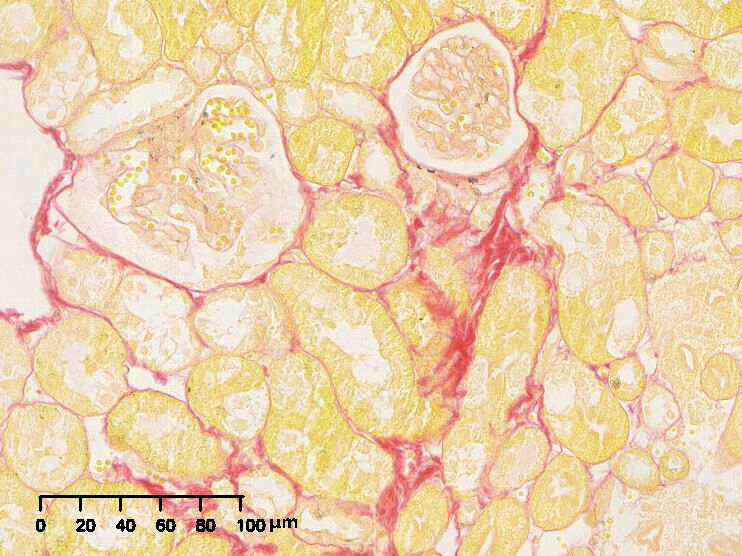

Supplement: Supplementary file 8 — Source data Fig. 3 [file 44321_2024_81_MOESM8_ESM.zip › Figure 3/3C/Sirius red/Insig1floxflox+56NX.tif]

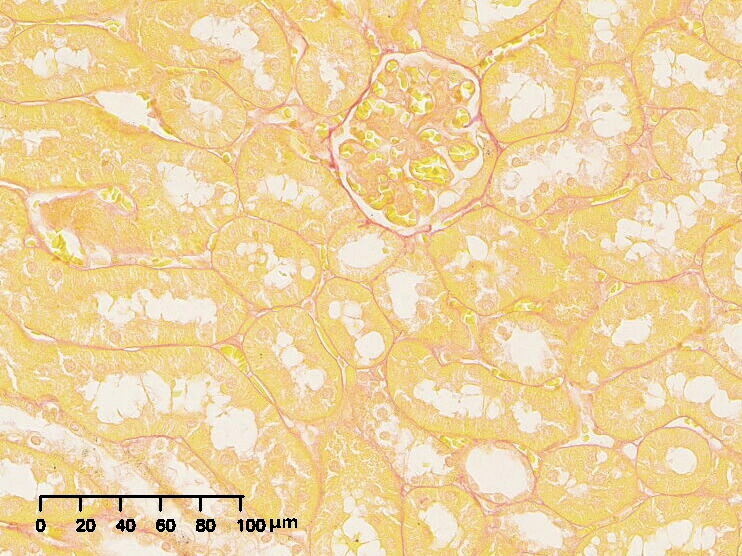

Supplement: Supplementary file 8 — Source data Fig. 3 [file 44321_2024_81_MOESM8_ESM.zip › Figure 3/3C/Sirius red/Insig1floxflox.tif]

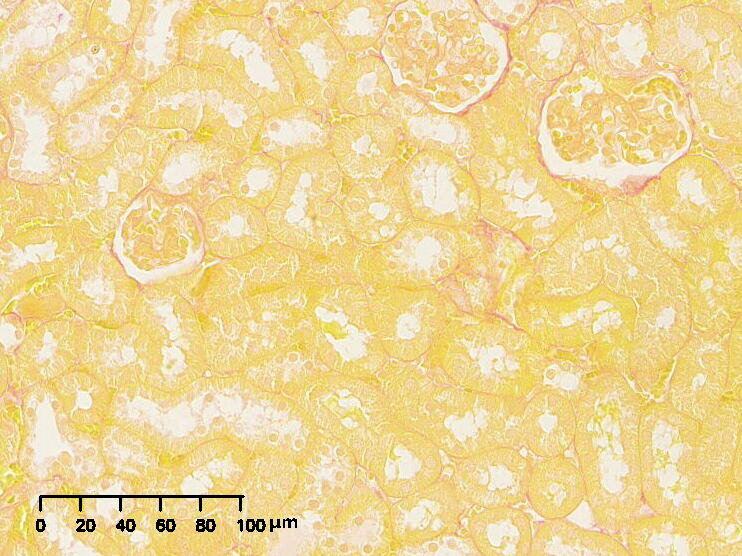

Supplement: Supplementary file 8 — Source data Fig. 3 [file 44321_2024_81_MOESM8_ESM.zip › Figure 3/3C/Sirius red/Insig1∩üäKap.tif]

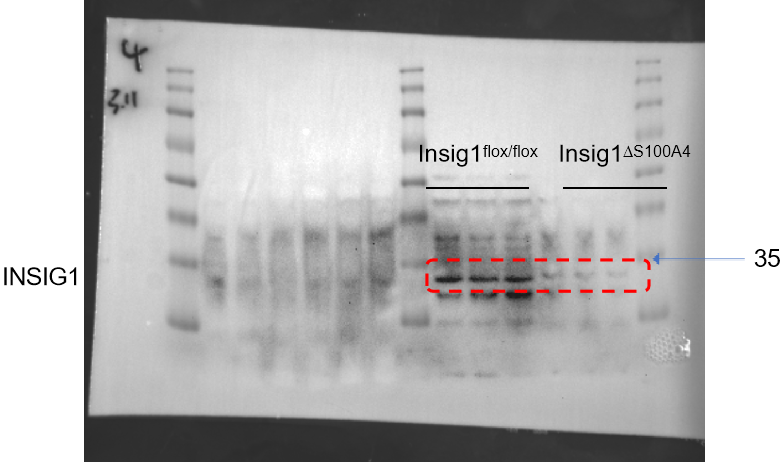

Supplement: Supplementary file 9 — Source data Fig. 4 [file 44321_2024_81_MOESM9_ESM.zip › Figure 4/4B/western INSIG1.tif]

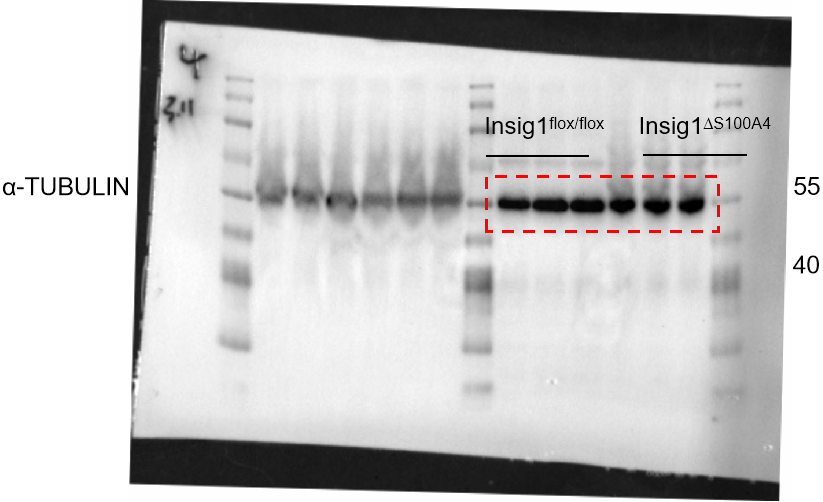

Supplement: Supplementary file 9 — Source data Fig. 4 [file 44321_2024_81_MOESM9_ESM.zip › Figure 4/4B/western ╬▒-TUBULIN.tif]

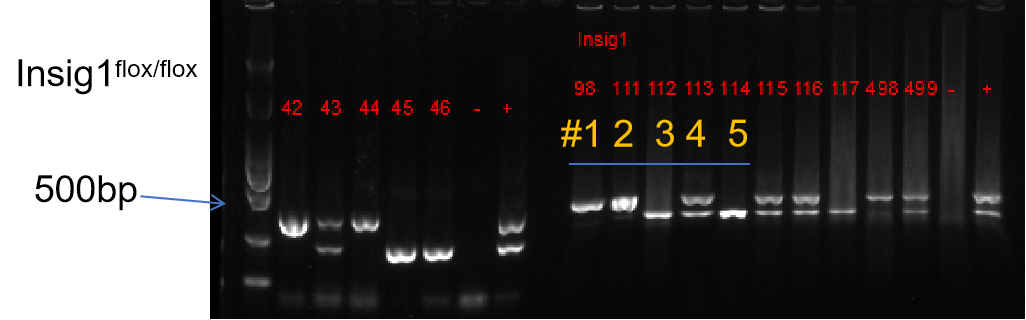

Supplement: Supplementary file 9 — Source data Fig. 4 [file 44321_2024_81_MOESM9_ESM.zip › Figure 4/4A/Gel Insig1floxflox.tif]

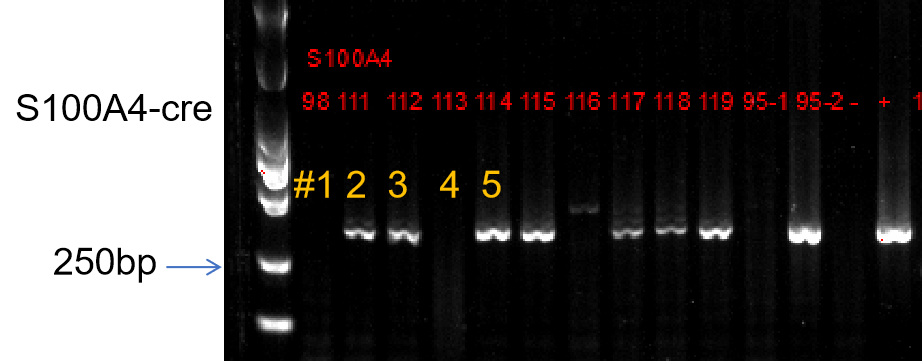

Supplement: Supplementary file 9 — Source data Fig. 4 [file 44321_2024_81_MOESM9_ESM.zip › Figure 4/4A/Gel S100A4-Cre.tif]

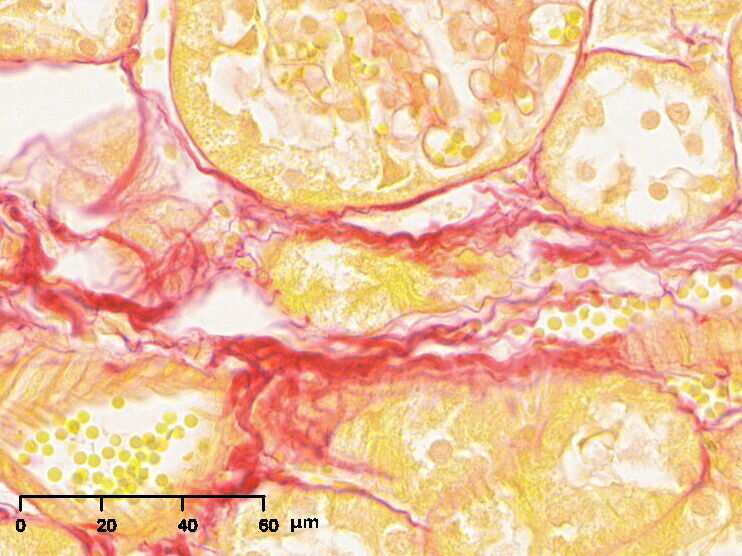

Supplement: Supplementary file 9 — Source data Fig. 4 [file 44321_2024_81_MOESM9_ESM.zip › Figure 4/4H/Insig1∩üäS100A4 +56NX.jpg]

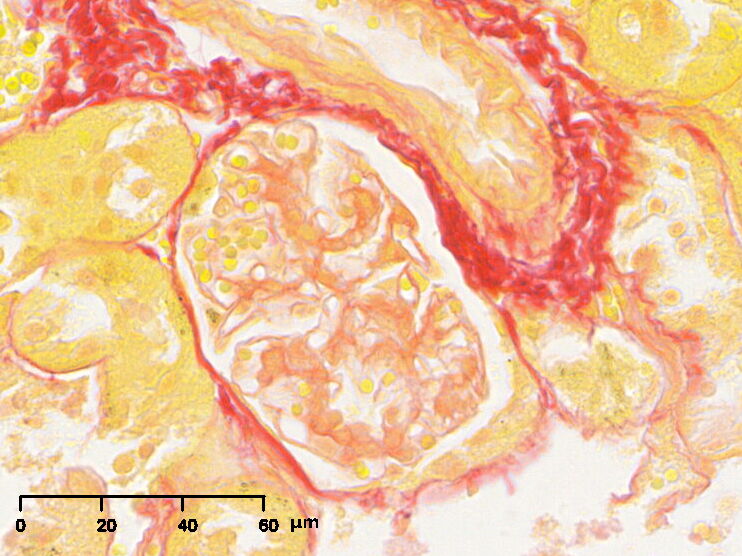

Supplement: Supplementary file 9 — Source data Fig. 4 [file 44321_2024_81_MOESM9_ESM.zip › Figure 4/4H/Insig1floxflox+56NX.jpg]

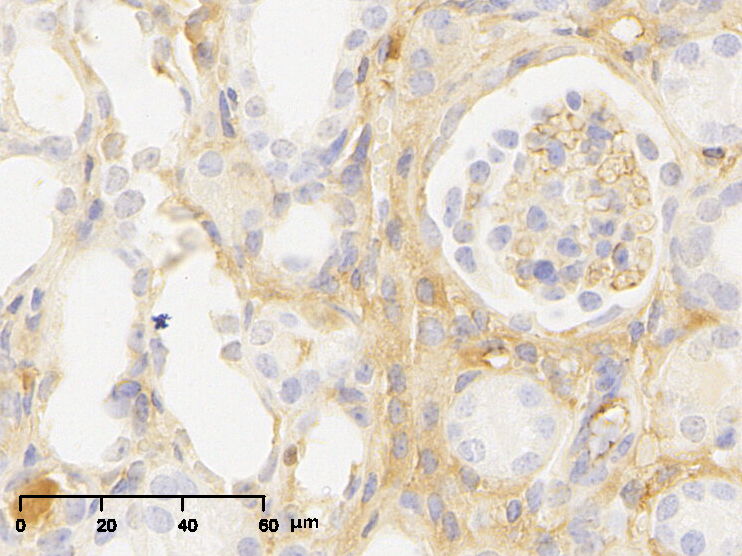

Supplement: Supplementary file 9 — Source data Fig. 4 [file 44321_2024_81_MOESM9_ESM.zip › Figure 4/4D/FN IHC/Insig1floxflox+UUO.jpg]

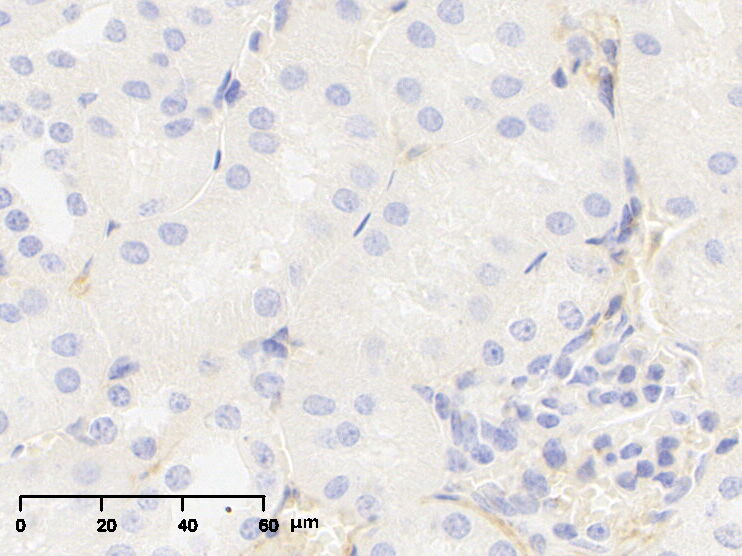

Supplement: Supplementary file 9 — Source data Fig. 4 [file 44321_2024_81_MOESM9_ESM.zip › Figure 4/4D/FN IHC/Insig1∩üäS100A4.jpg]

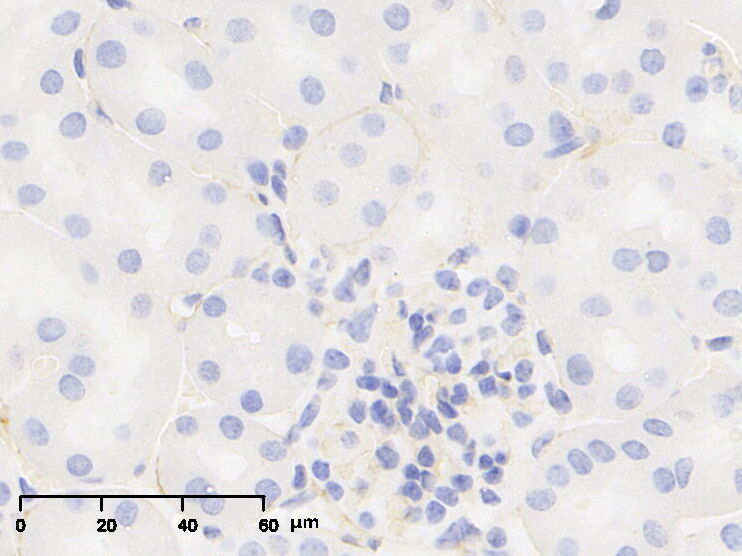

Supplement: Supplementary file 9 — Source data Fig. 4 [file 44321_2024_81_MOESM9_ESM.zip › Figure 4/4D/FN IHC/Insig1floxflox.jpg]

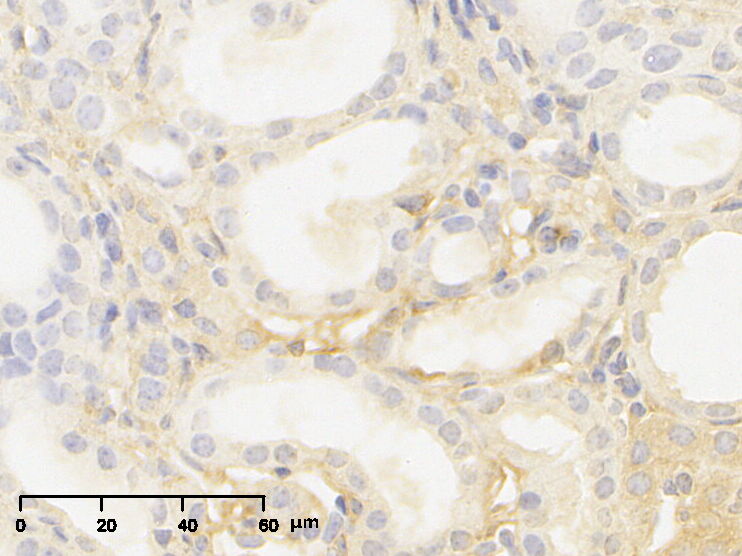

Supplement: Supplementary file 9 — Source data Fig. 4 [file 44321_2024_81_MOESM9_ESM.zip › Figure 4/4D/FN IHC/Insig1∩üäS100A4+UUO.jpg]

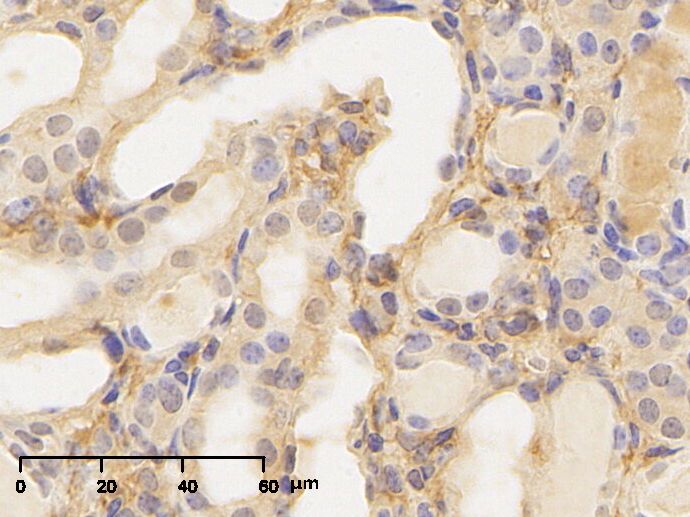

Supplement: Supplementary file 9 — Source data Fig. 4 [file 44321_2024_81_MOESM9_ESM.zip › Figure 4/4D/╬▒-SMA IHC/Insig1floxflox+UUO.jpg]

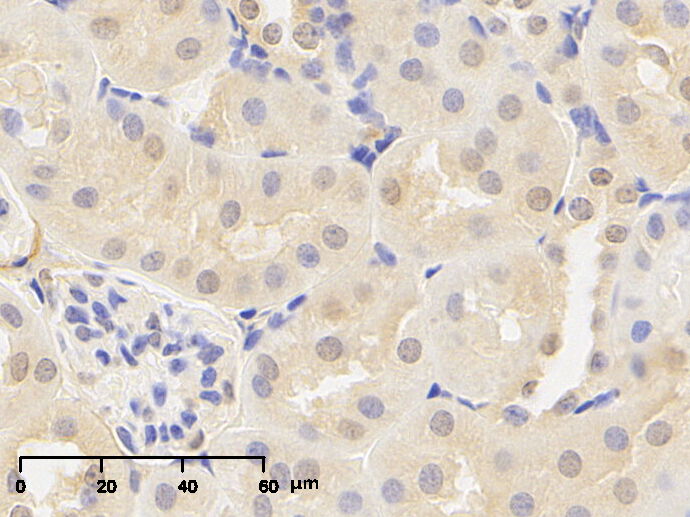

Supplement: Supplementary file 9 — Source data Fig. 4 [file 44321_2024_81_MOESM9_ESM.zip › Figure 4/4D/╬▒-SMA IHC/Insig1∩üäS100A4.jpg]

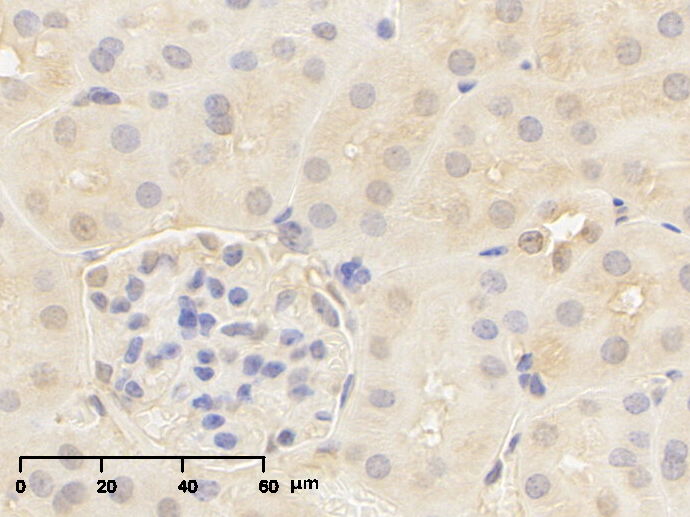

Supplement: Supplementary file 9 — Source data Fig. 4 [file 44321_2024_81_MOESM9_ESM.zip › Figure 4/4D/╬▒-SMA IHC/Insig1floxflox.jpg]

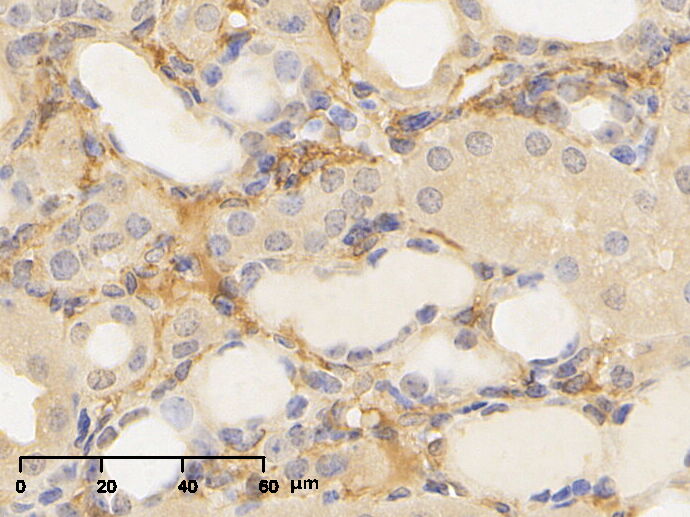

Supplement: Supplementary file 9 — Source data Fig. 4 [file 44321_2024_81_MOESM9_ESM.zip › Figure 4/4D/╬▒-SMA IHC/Insig1∩üäS100A4+UUO.jpg]

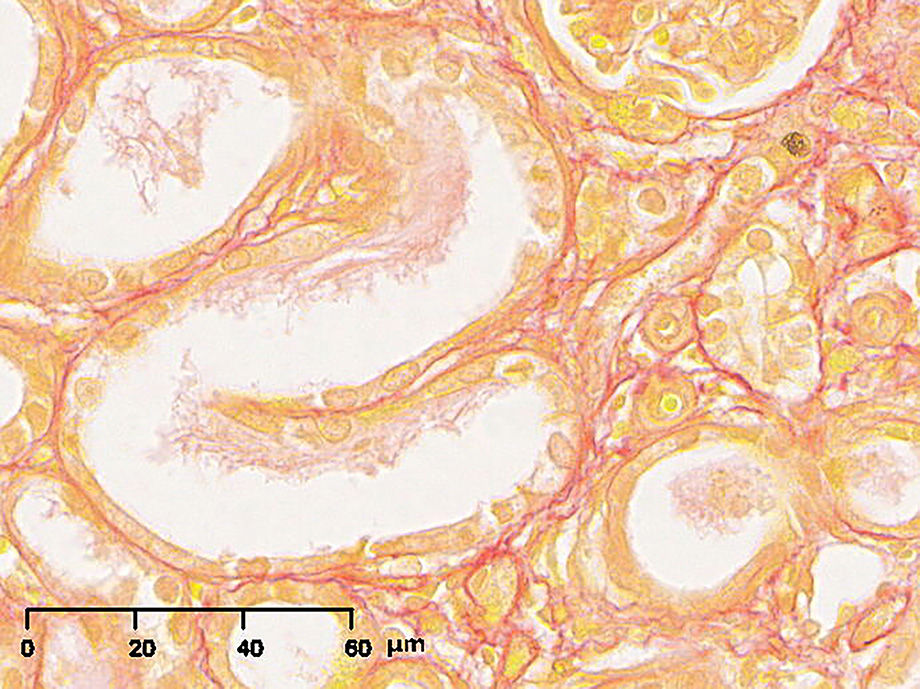

Supplement: Supplementary file 9 — Source data Fig. 4 [file 44321_2024_81_MOESM9_ESM.zip › Figure 4/4D/Sirius red/Insig1floxflox+UUO.jpg]

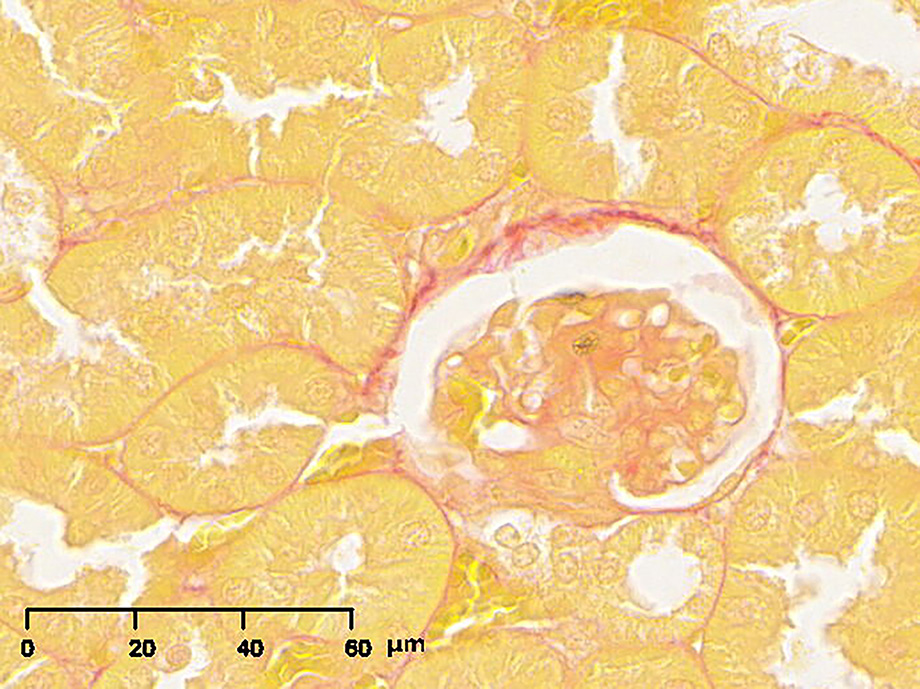

Supplement: Supplementary file 9 — Source data Fig. 4 [file 44321_2024_81_MOESM9_ESM.zip › Figure 4/4D/Sirius red/Insig1∩üäS100A4.jpg]

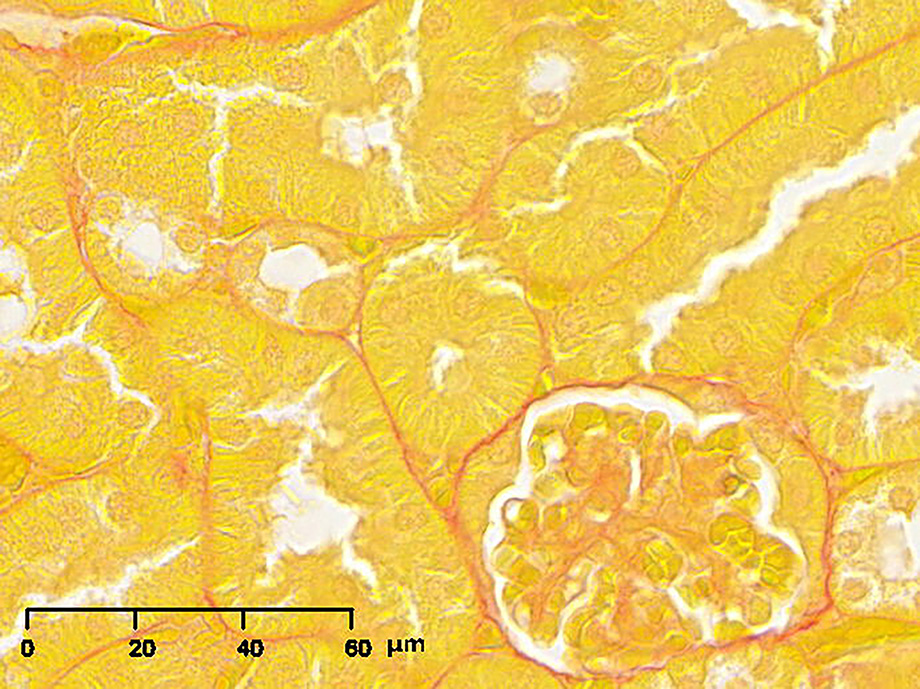

Supplement: Supplementary file 9 — Source data Fig. 4 [file 44321_2024_81_MOESM9_ESM.zip › Figure 4/4D/Sirius red/Insig1floxflox.jpg]

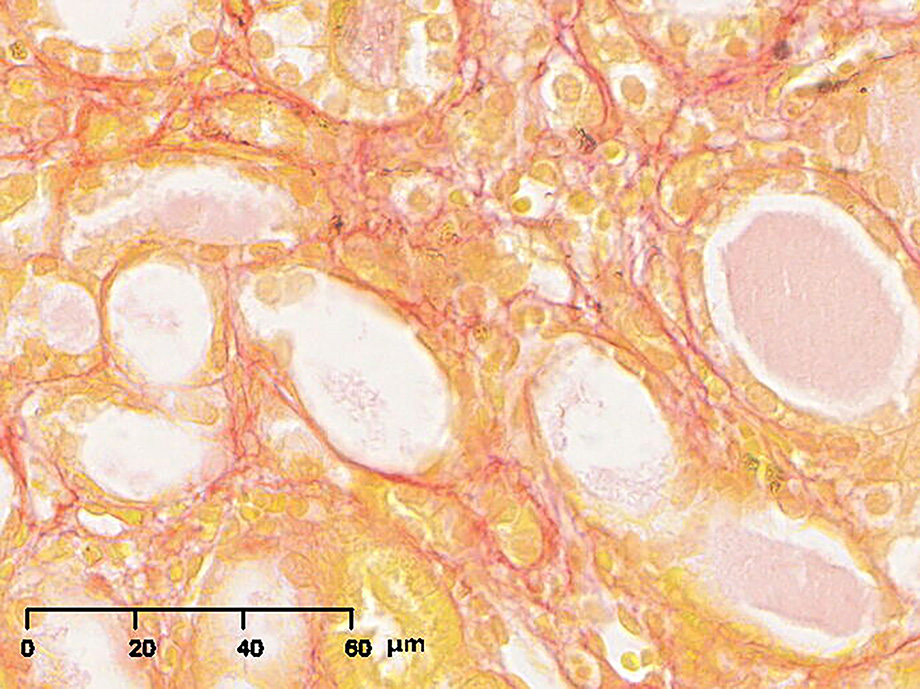

Supplement: Supplementary file 9 — Source data Fig. 4 [file 44321_2024_81_MOESM9_ESM.zip › Figure 4/4D/Sirius red/Insig1∩üäS100A4+UUO.jpg]

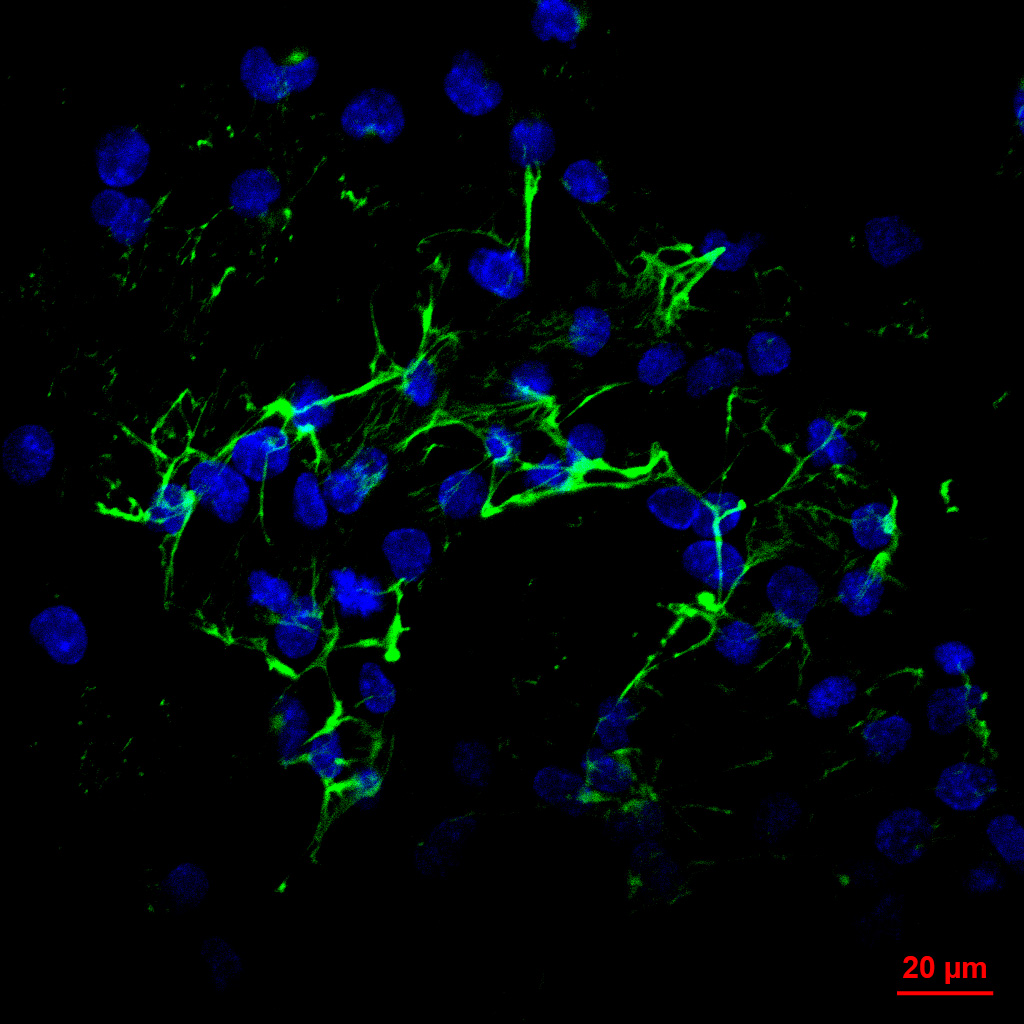

Supplement: Supplementary file 10 — Source data Fig. 5 [file 44321_2024_81_MOESM10_ESM.zip › Figure 5/5I/NC+TGF-╬▓1.jpg]

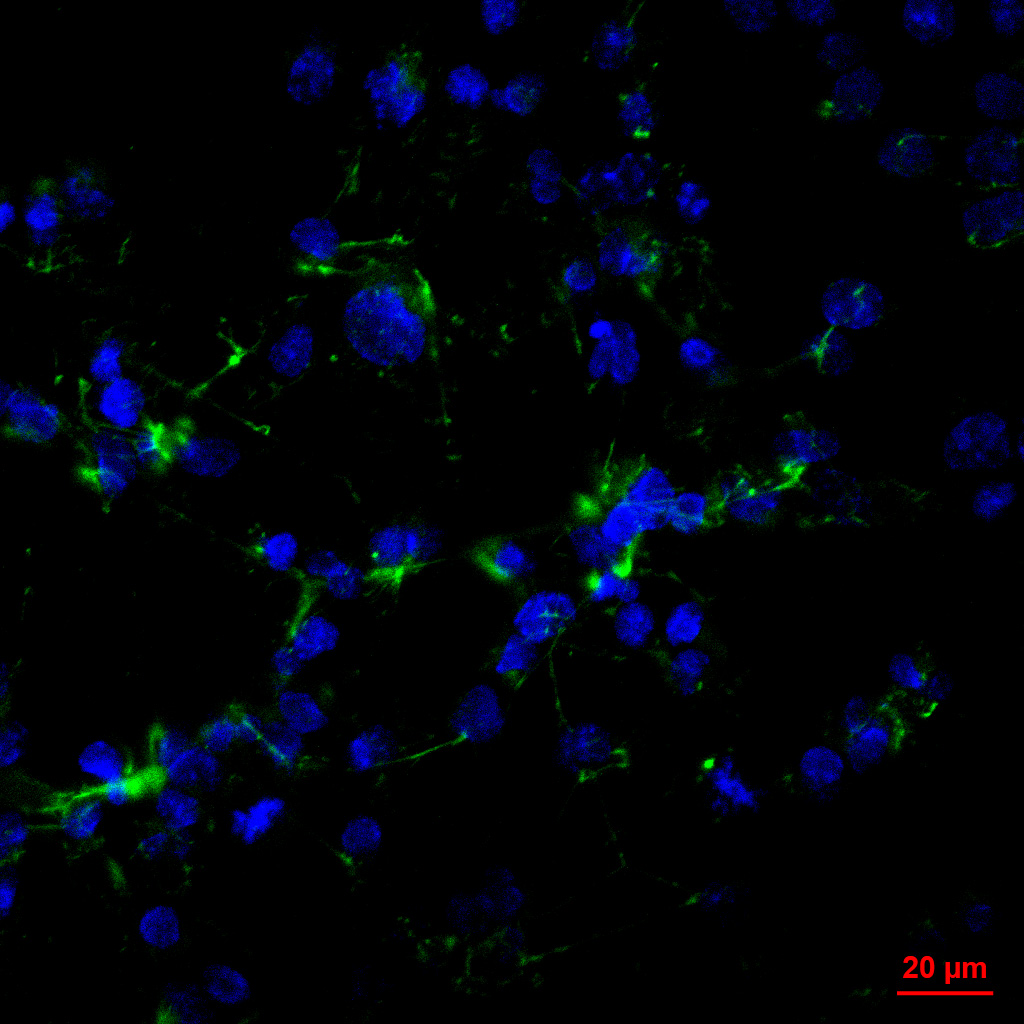

Supplement: Supplementary file 10 — Source data Fig. 5 [file 44321_2024_81_MOESM10_ESM.zip › Figure 5/5I/Insig1+TGF-╬▓1.jpg]

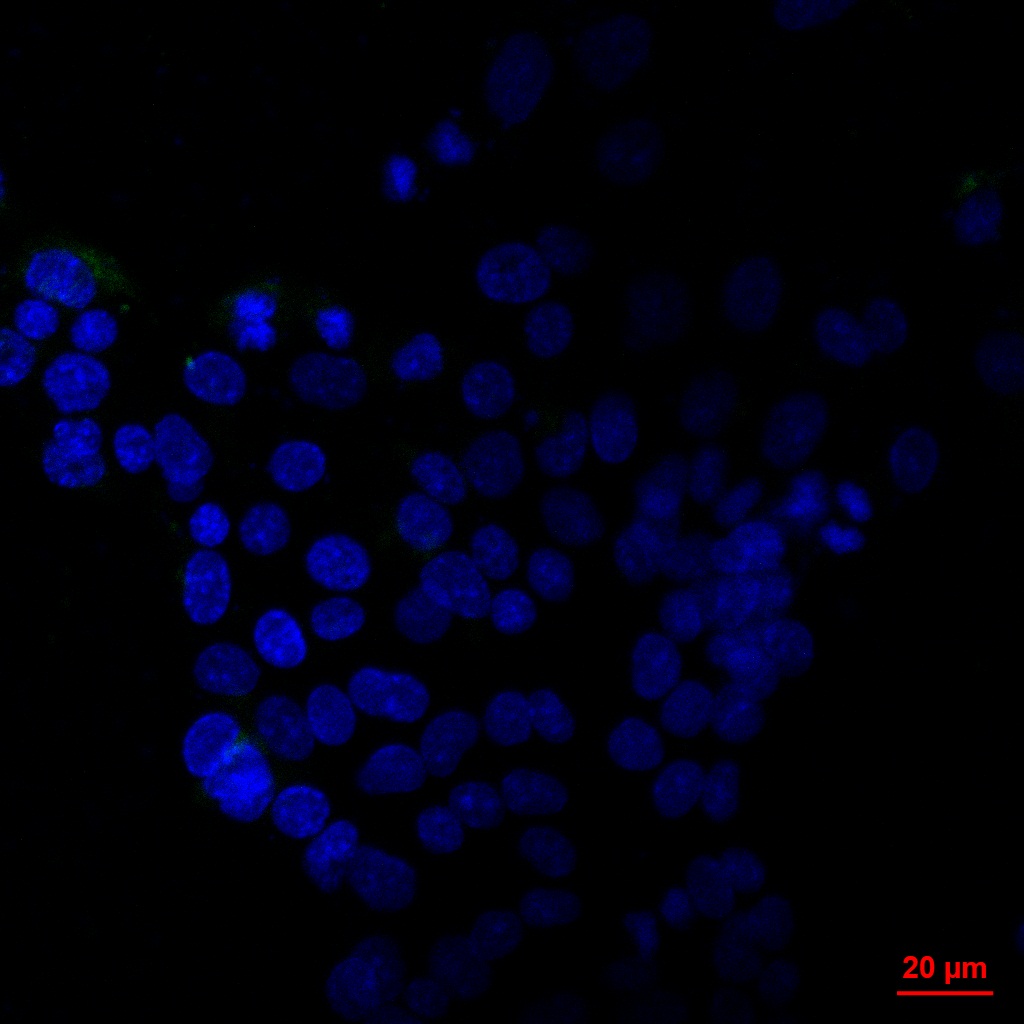

Supplement: Supplementary file 10 — Source data Fig. 5 [file 44321_2024_81_MOESM10_ESM.zip › Figure 5/5I/Insig1.jpg]

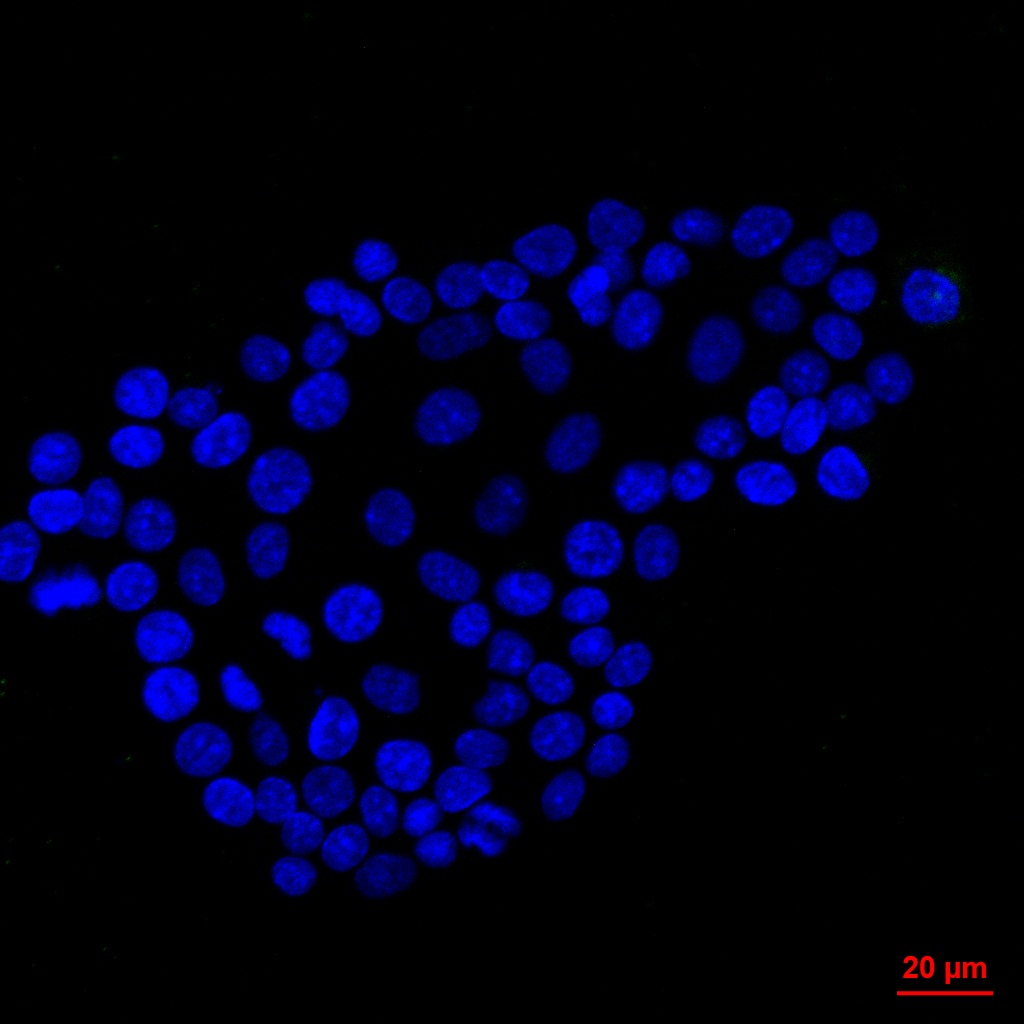

Supplement: Supplementary file 10 — Source data Fig. 5 [file 44321_2024_81_MOESM10_ESM.zip › Figure 5/5I/NC.jpg]

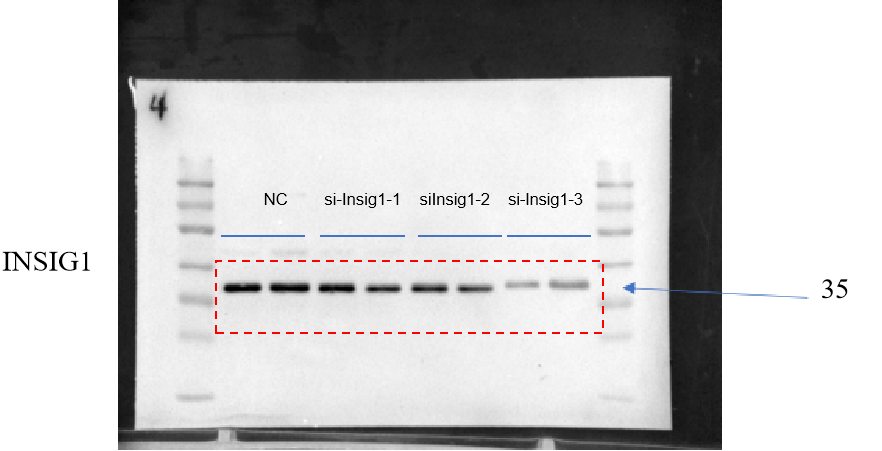

Supplement: Supplementary file 10 — Source data Fig. 5 [file 44321_2024_81_MOESM10_ESM.zip › Figure 5/5A/western INSIG1.tif]

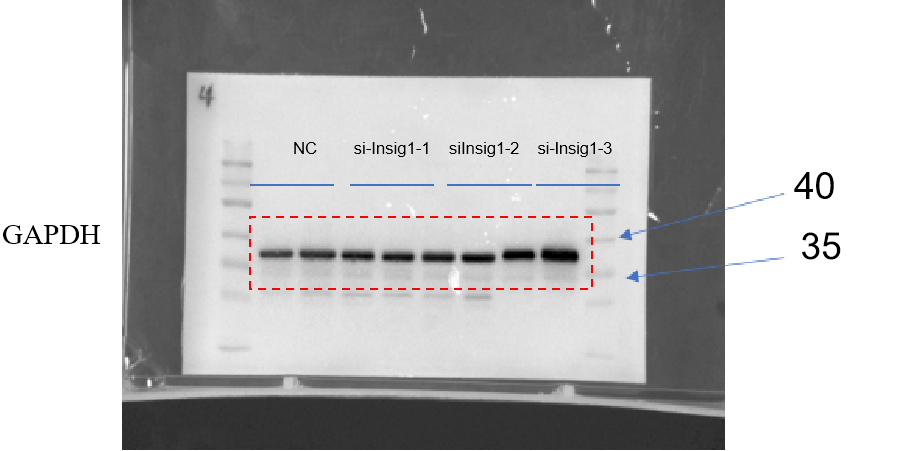

Supplement: Supplementary file 10 — Source data Fig. 5 [file 44321_2024_81_MOESM10_ESM.zip › Figure 5/5A/western GAPDH.tif]

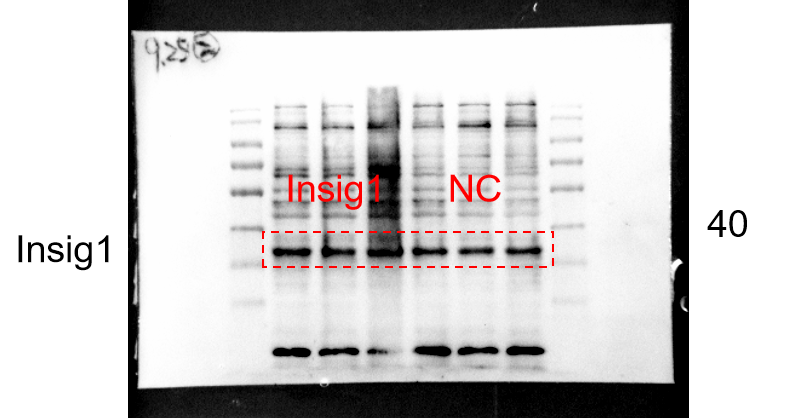

Supplement: Supplementary file 10 — Source data Fig. 5 [file 44321_2024_81_MOESM10_ESM.zip › Figure 5/5F/western INSIG1.tif]

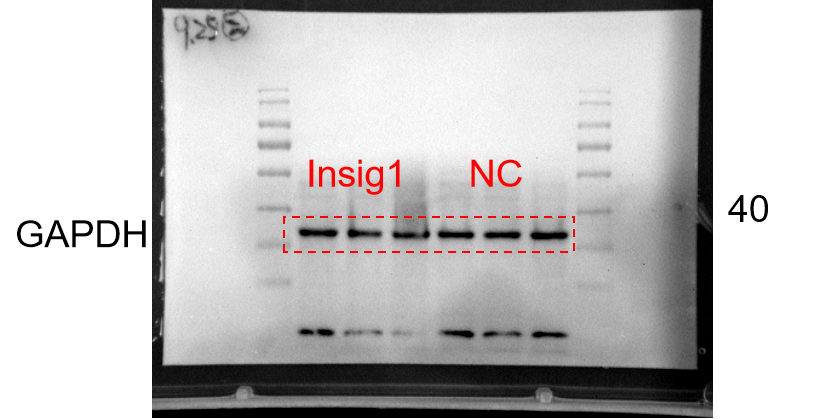

Supplement: Supplementary file 10 — Source data Fig. 5 [file 44321_2024_81_MOESM10_ESM.zip › Figure 5/5F/western GAPDH.tif]

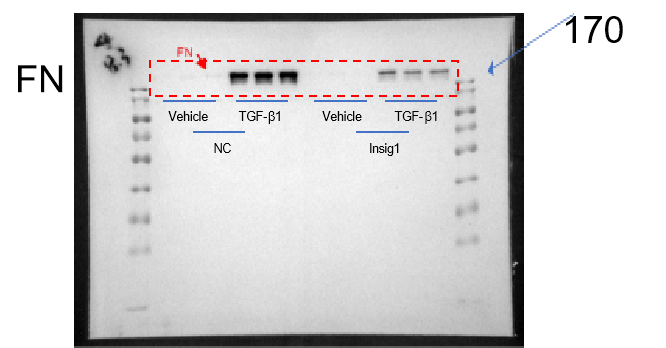

Supplement: Supplementary file 10 — Source data Fig. 5 [file 44321_2024_81_MOESM10_ESM.zip › Figure 5/5H/western FN.tif]

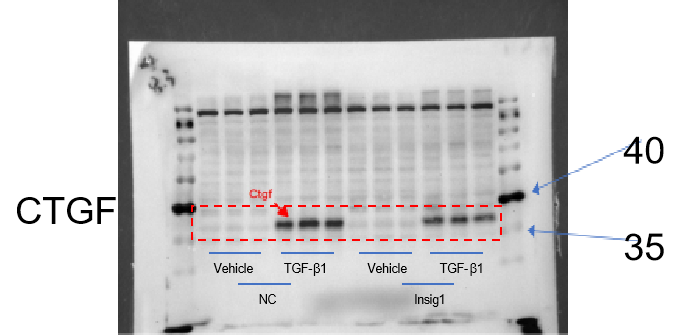

Supplement: Supplementary file 10 — Source data Fig. 5 [file 44321_2024_81_MOESM10_ESM.zip › Figure 5/5H/western CTGF.tif]

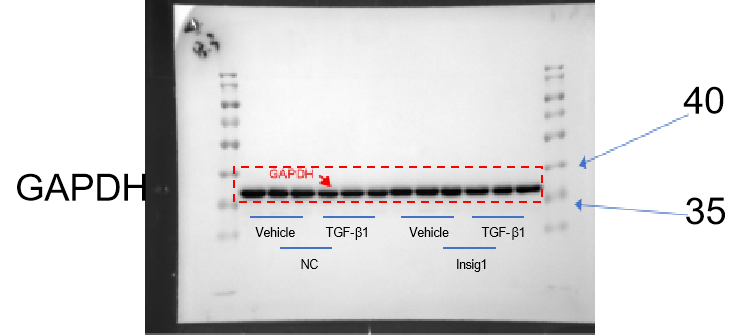

Supplement: Supplementary file 10 — Source data Fig. 5 [file 44321_2024_81_MOESM10_ESM.zip › Figure 5/5H/western GAPDH.tif]

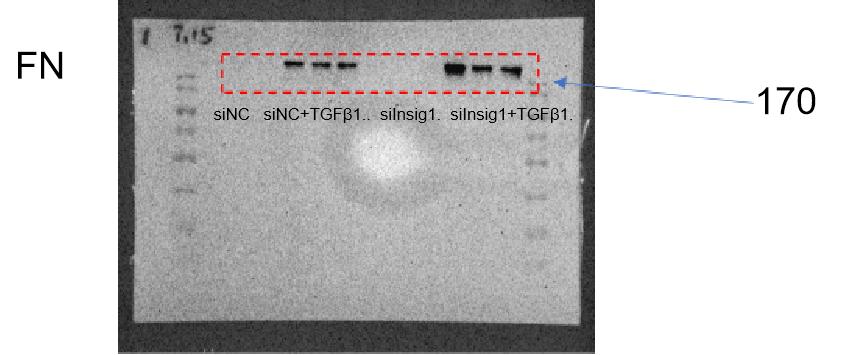

Supplement: Supplementary file 10 — Source data Fig. 5 [file 44321_2024_81_MOESM10_ESM.zip › Figure 5/5C/western FN.tif]

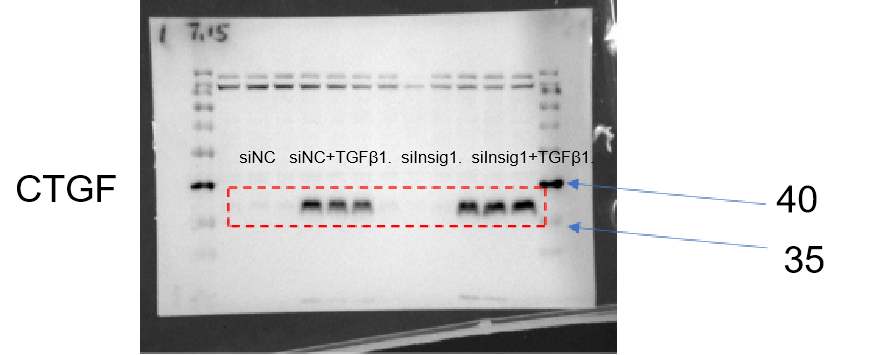

Supplement: Supplementary file 10 — Source data Fig. 5 [file 44321_2024_81_MOESM10_ESM.zip › Figure 5/5C/western CTGF.tif]

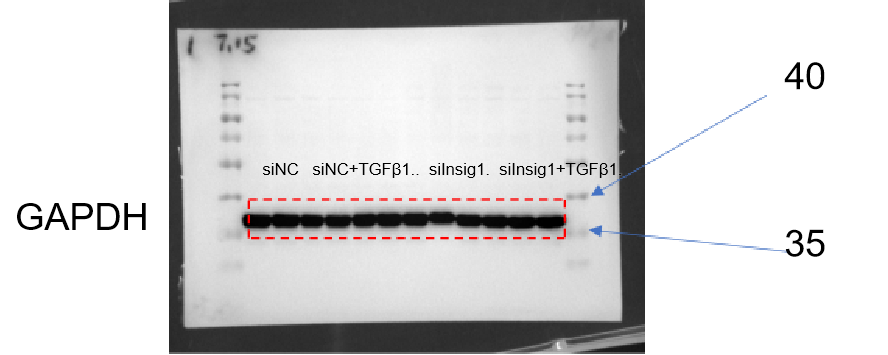

Supplement: Supplementary file 10 — Source data Fig. 5 [file 44321_2024_81_MOESM10_ESM.zip › Figure 5/5C/western GAPDH.tif]

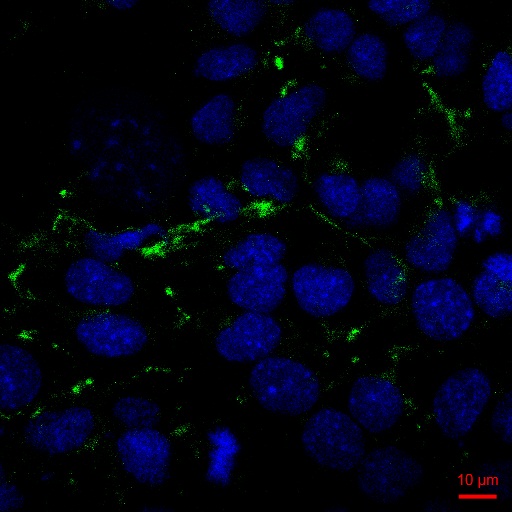

Supplement: Supplementary file 10 — Source data Fig. 5 [file 44321_2024_81_MOESM10_ESM.zip › Figure 5/5D/si-NC+TGF-╬▓1.jpg]

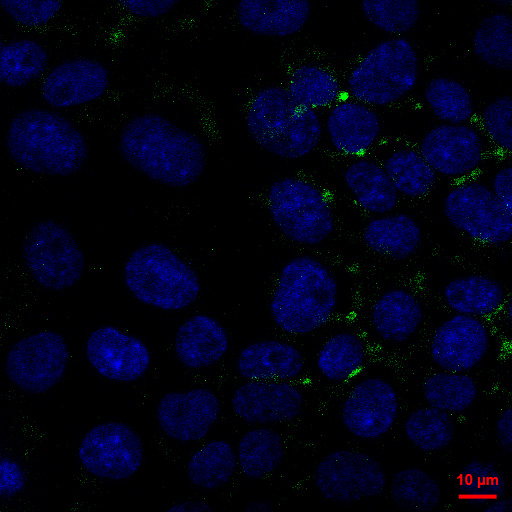

Supplement: Supplementary file 10 — Source data Fig. 5 [file 44321_2024_81_MOESM10_ESM.zip › Figure 5/5D/si-Insig1.jpg]

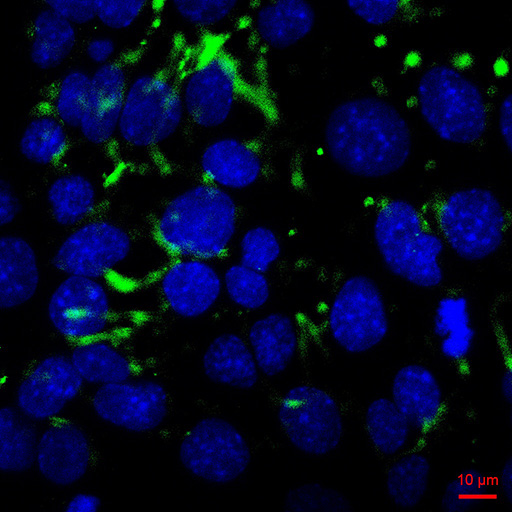

Supplement: Supplementary file 10 — Source data Fig. 5 [file 44321_2024_81_MOESM10_ESM.zip › Figure 5/5D/si-Insig1+TGF-╬▓1.jpg]

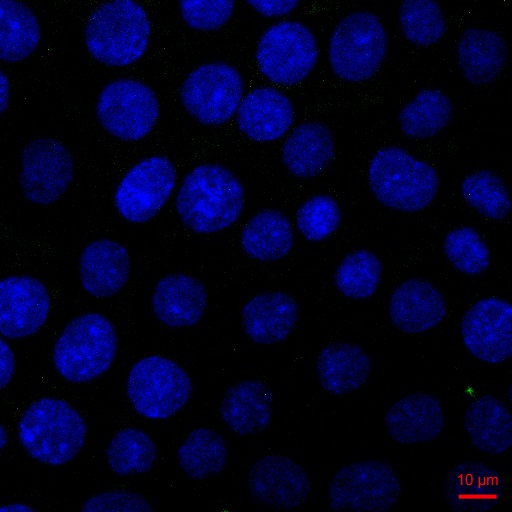

Supplement: Supplementary file 10 — Source data Fig. 5 [file 44321_2024_81_MOESM10_ESM.zip › Figure 5/5D/si-NC.jpg]

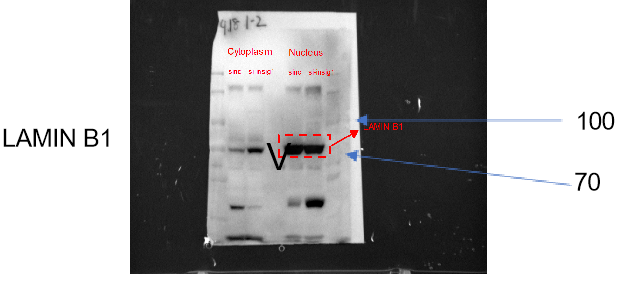

Supplement: Supplementary file 11 — Source data Fig. 6 [file 44321_2024_81_MOESM11_ESM.zip › Figure 6/6H/western LAMIN B1.tif]

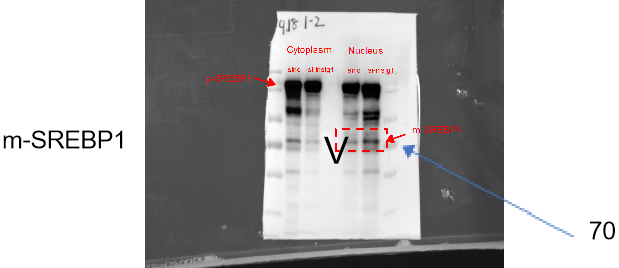

Supplement: Supplementary file 11 — Source data Fig. 6 [file 44321_2024_81_MOESM11_ESM.zip › Figure 6/6H/western m-SREBP1.tif]

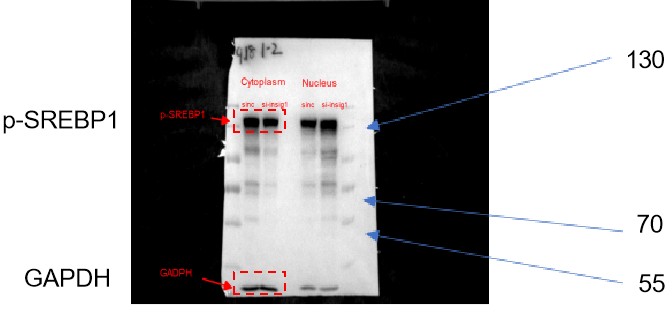

Supplement: Supplementary file 11 — Source data Fig. 6 [file 44321_2024_81_MOESM11_ESM.zip › Figure 6/6H/western p-SREBP1,GAPDH.tif]

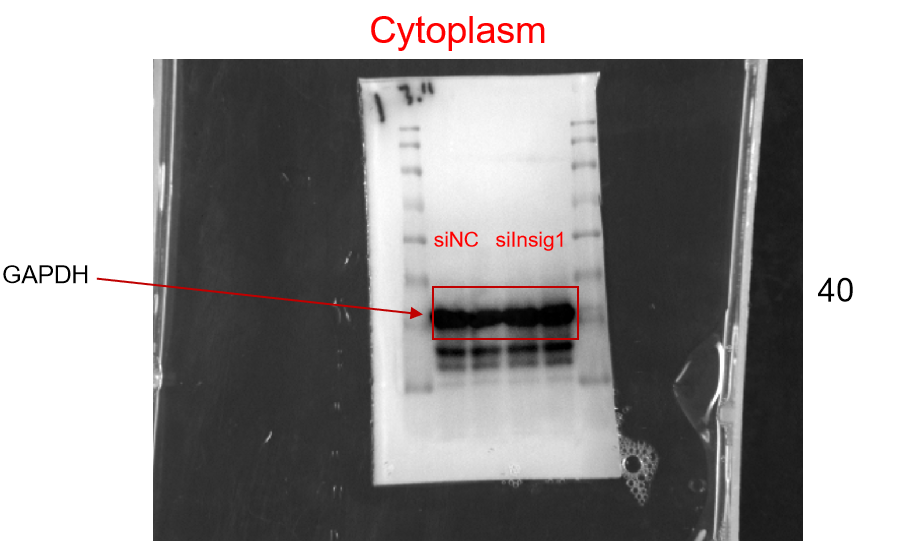

Supplement: Supplementary file 11 — Source data Fig. 6 [file 44321_2024_81_MOESM11_ESM.zip › Figure 6/6H/repeat/western GAPDH repeat.tif]

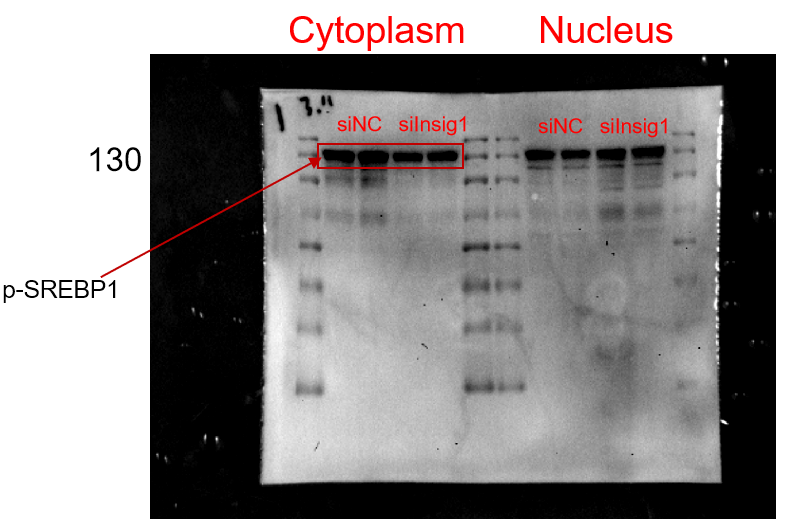

Supplement: Supplementary file 11 — Source data Fig. 6 [file 44321_2024_81_MOESM11_ESM.zip › Figure 6/6H/repeat/western p-SREBP1 repeat.tif]

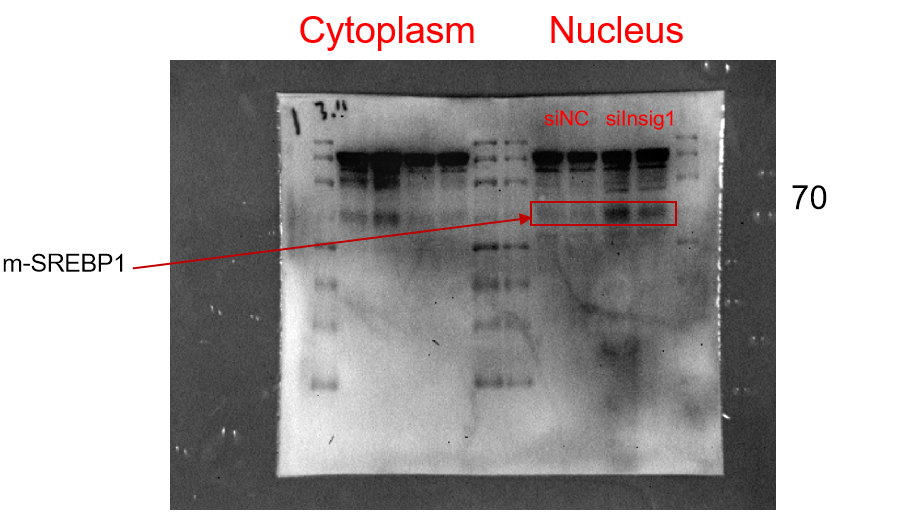

Supplement: Supplementary file 11 — Source data Fig. 6 [file 44321_2024_81_MOESM11_ESM.zip › Figure 6/6H/repeat/western m-SREBP1 repeat.tif]

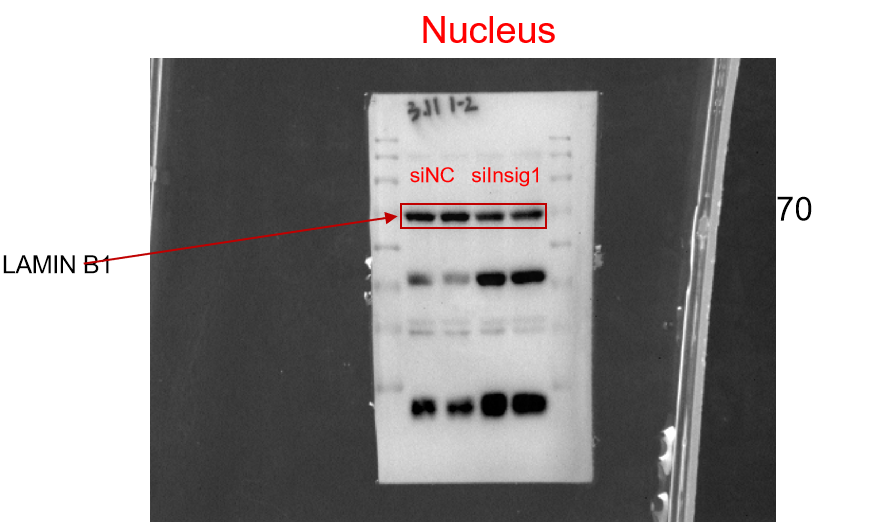

Supplement: Supplementary file 11 — Source data Fig. 6 [file 44321_2024_81_MOESM11_ESM.zip › Figure 6/6H/repeat/western LAMIN B1 repeat.tif]

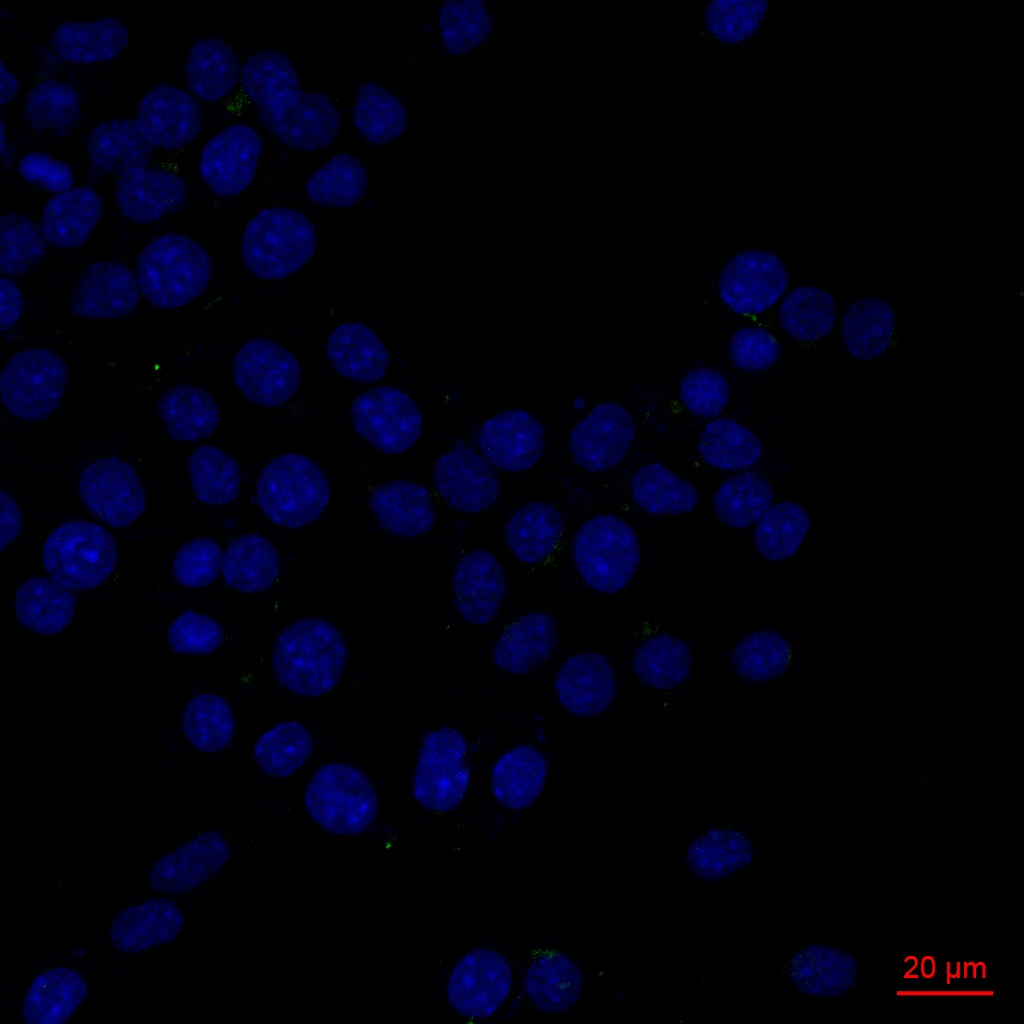

Supplement: Supplementary file 12 — Source data Fig. 7 [file 44321_2024_81_MOESM12_ESM.zip › Figure 7/7J/Aldh1a1.jpg]

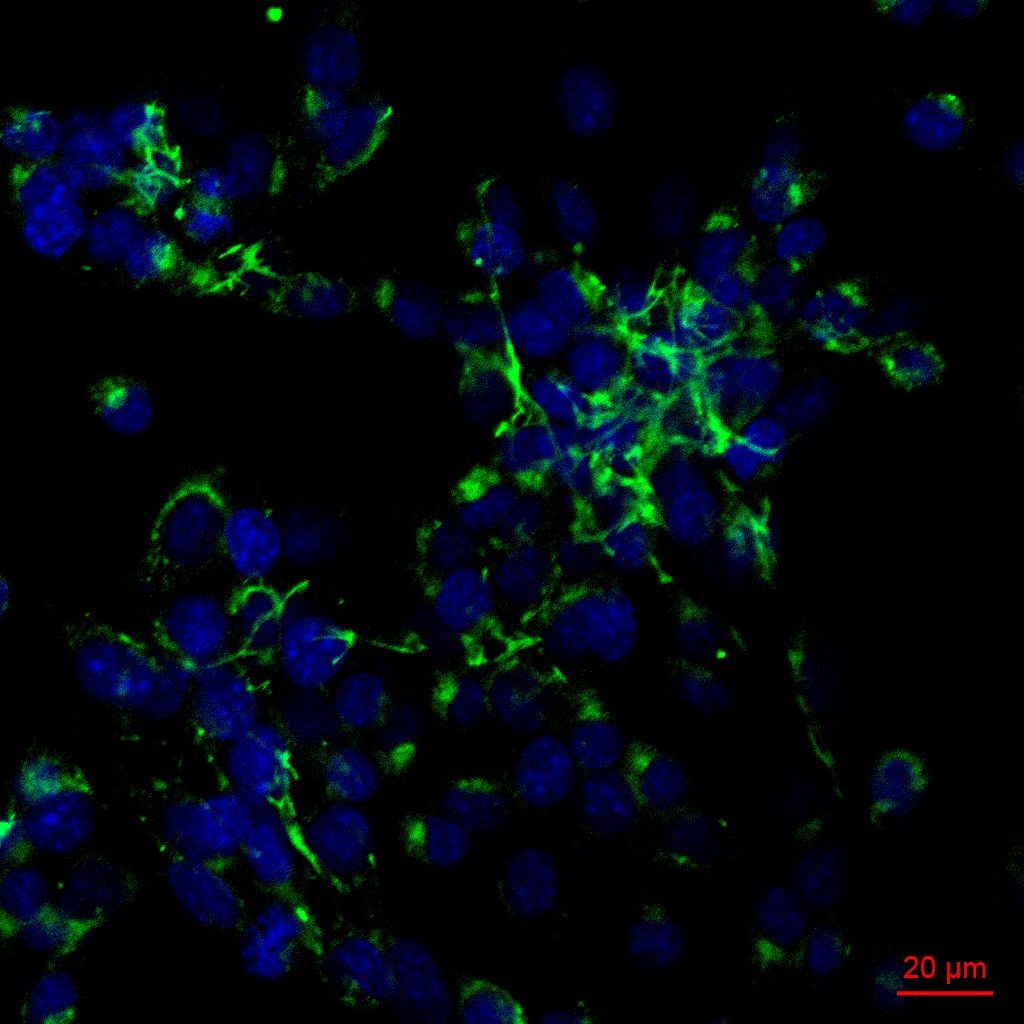

Supplement: Supplementary file 12 — Source data Fig. 7 [file 44321_2024_81_MOESM12_ESM.zip › Figure 7/7J/Aldh1a1+TGF-╬▓1.jpg]

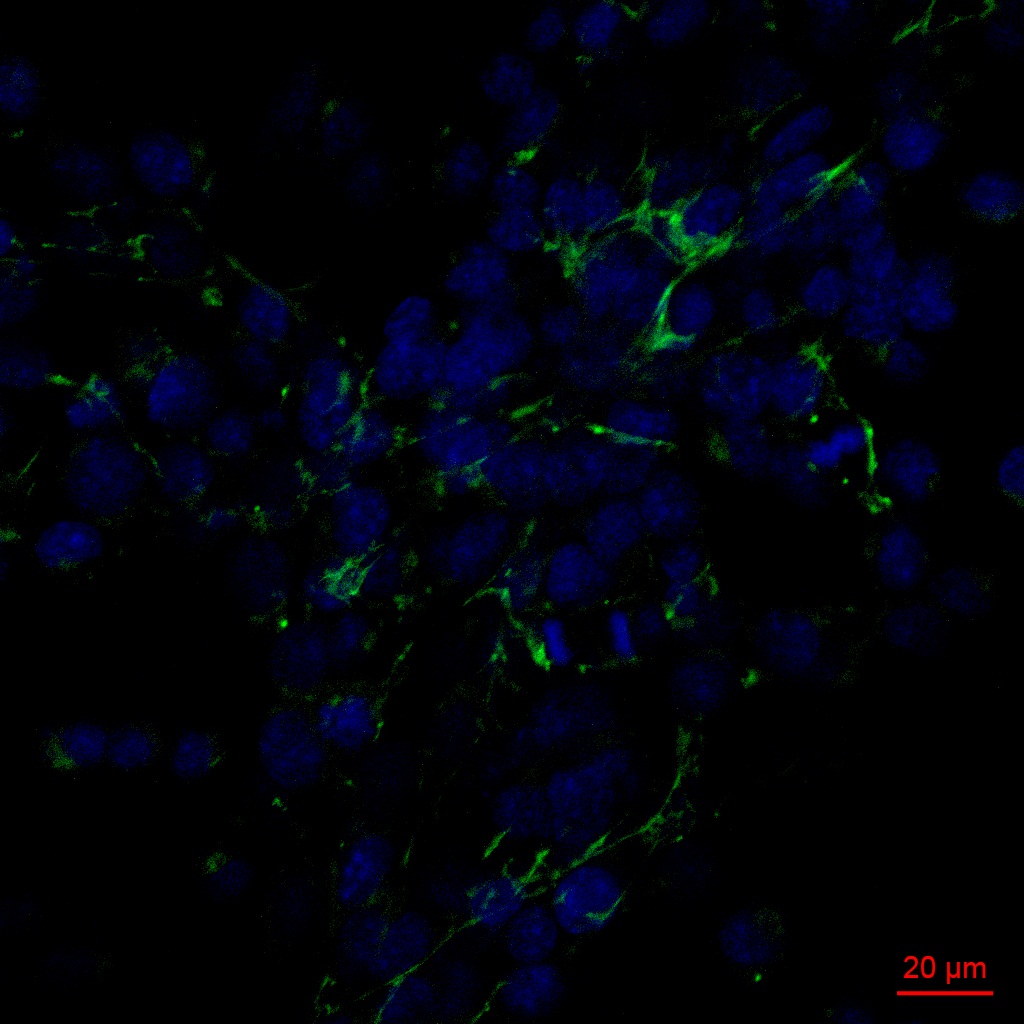

Supplement: Supplementary file 12 — Source data Fig. 7 [file 44321_2024_81_MOESM12_ESM.zip › Figure 7/7J/NC+TGF-╬▓1.jpg]

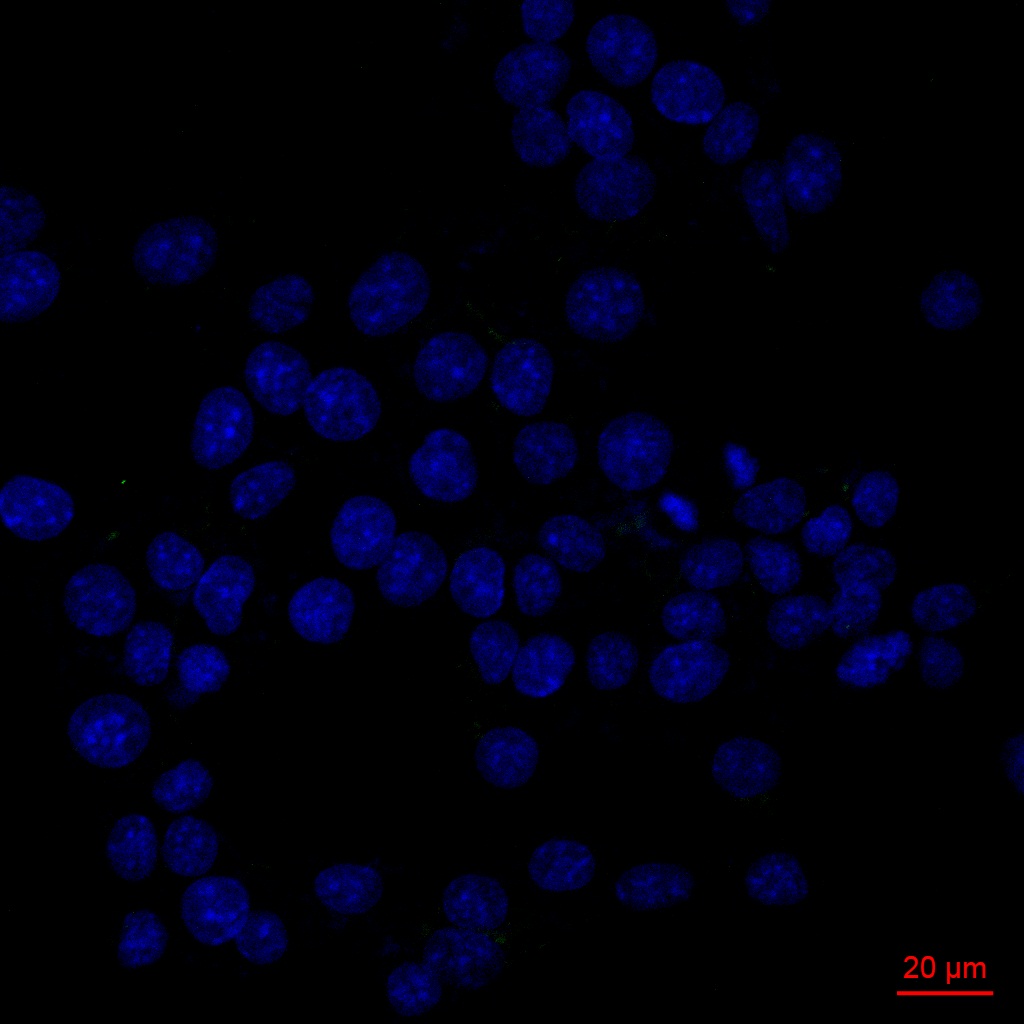

Supplement: Supplementary file 12 — Source data Fig. 7 [file 44321_2024_81_MOESM12_ESM.zip › Figure 7/7J/NC.jpg]

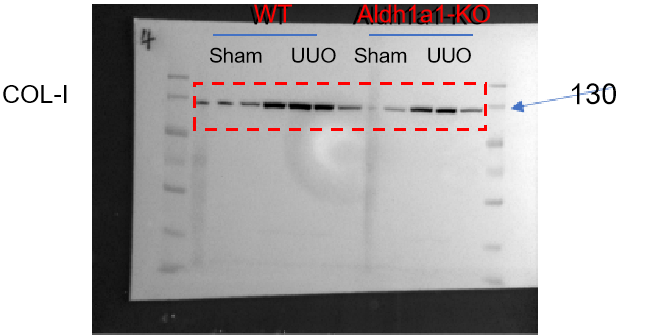

Supplement: Supplementary file 12 — Source data Fig. 7 [file 44321_2024_81_MOESM12_ESM.zip › Figure 7/7E/western COL-I.tif]

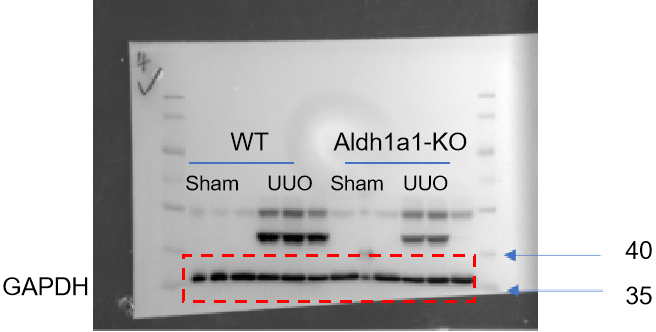

Supplement: Supplementary file 12 — Source data Fig. 7 [file 44321_2024_81_MOESM12_ESM.zip › Figure 7/7E/western GAPDH.tif]

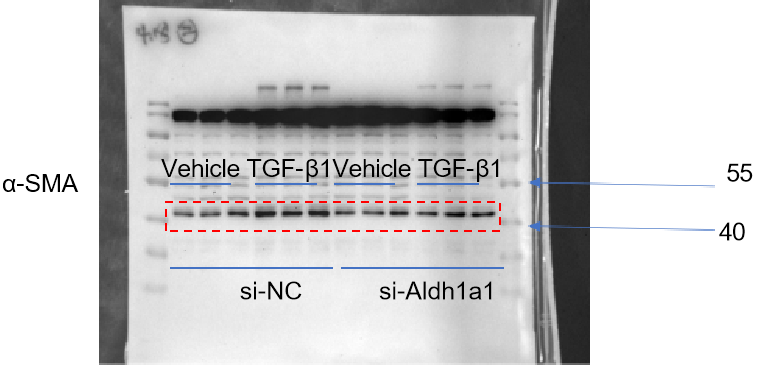

Supplement: Supplementary file 12 — Source data Fig. 7 [file 44321_2024_81_MOESM12_ESM.zip › Figure 7/7L/western ╬▒-SMA.tif]

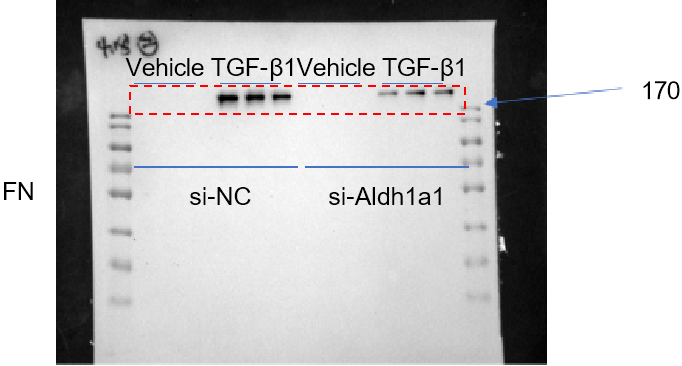

Supplement: Supplementary file 12 — Source data Fig. 7 [file 44321_2024_81_MOESM12_ESM.zip › Figure 7/7L/western FN.tif]

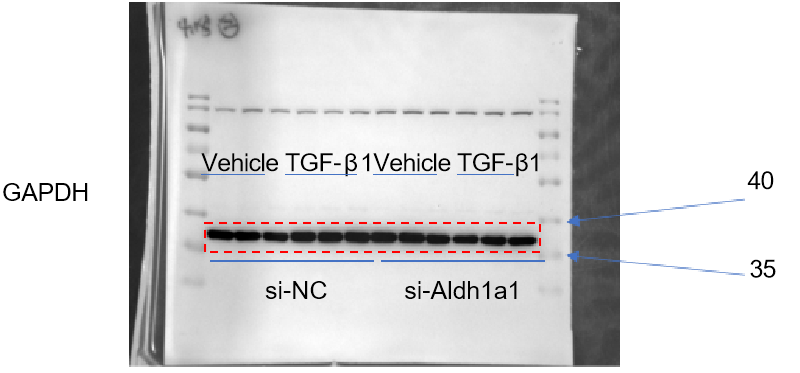

Supplement: Supplementary file 12 — Source data Fig. 7 [file 44321_2024_81_MOESM12_ESM.zip › Figure 7/7L/western GAPDH.tif]

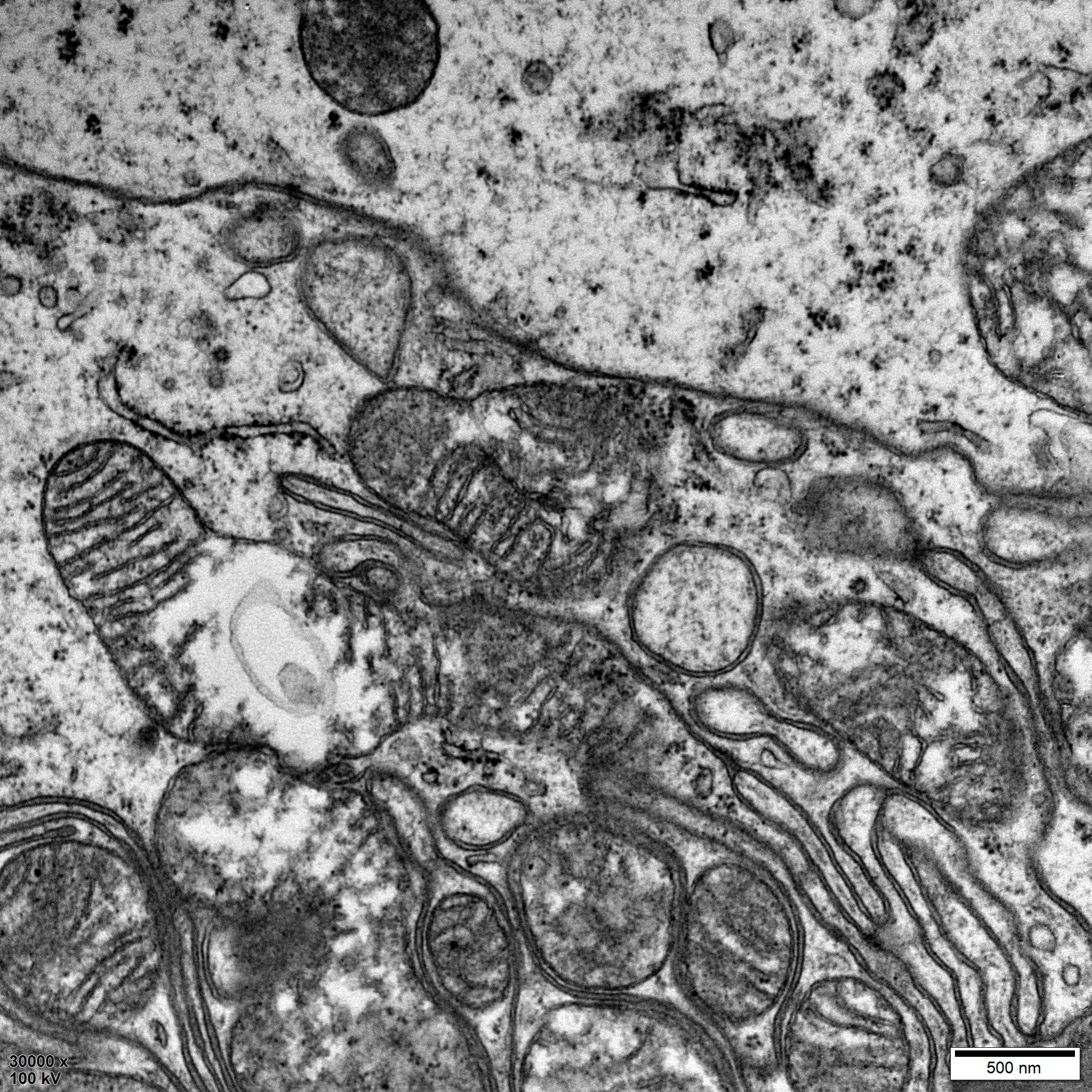

Supplement: Supplementary file 12 — Source data Fig. 7 [file 44321_2024_81_MOESM12_ESM.zip › Figure 7/7G/Aldh1a1-KO UUO.jpg]
