# Supplementary figures and images for: Tubular insulin-induced gene 1 deficiency promotes NAD+ consumption and exacerbates kidney fibrosis (part 2 of 2)
Source: EMBO Mol Med. 2024 May 28;16(7):11. doi: 10.1038/s44321-024-00081-7 (PMC11251182; doi:10.1038/s44321-024-00081-7)

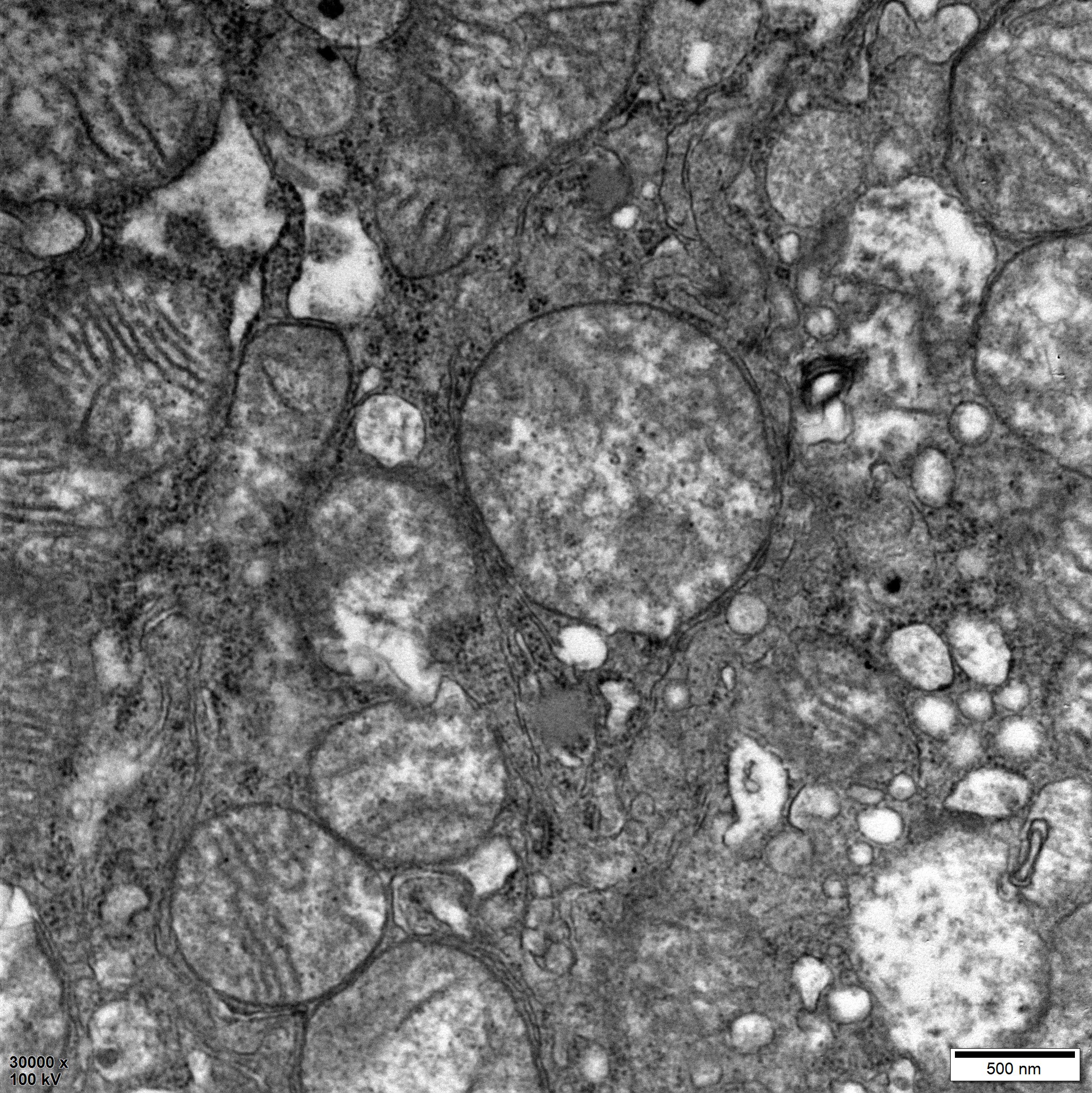

Supplement: Supplementary file 12 — Source data Fig. 7 [file 44321_2024_81_MOESM12_ESM.zip › Figure 7/7G/WT UUO.jpg]

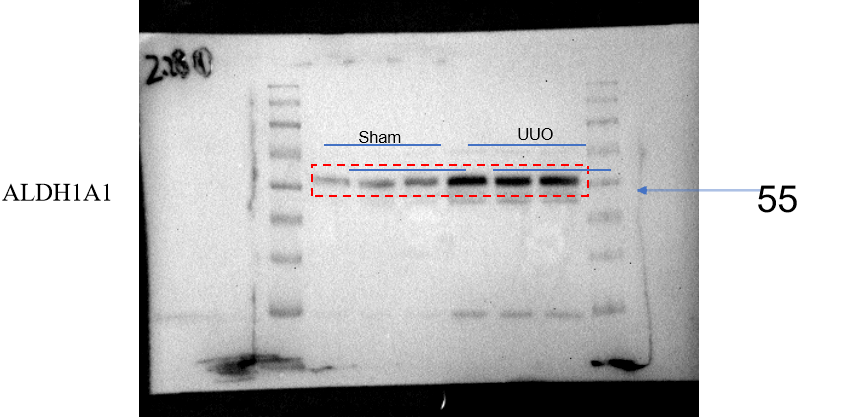

Supplement: Supplementary file 12 — Source data Fig. 7 [file 44321_2024_81_MOESM12_ESM.zip › Figure 7/7A/western ALDH1A1.tif]

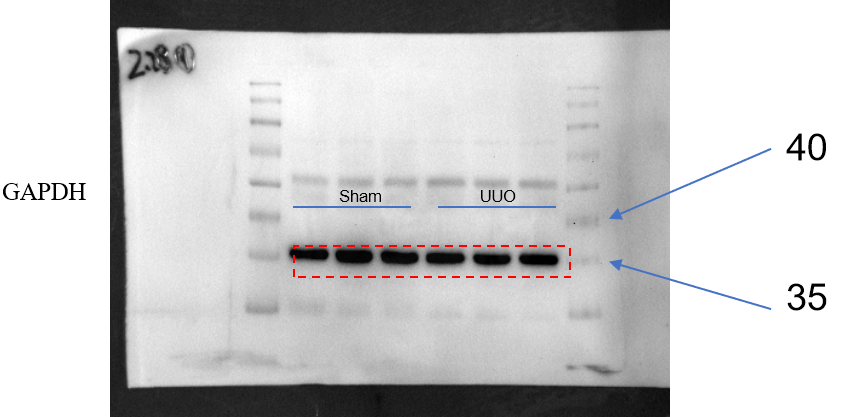

Supplement: Supplementary file 12 — Source data Fig. 7 [file 44321_2024_81_MOESM12_ESM.zip › Figure 7/7A/western GAPDH.tif]

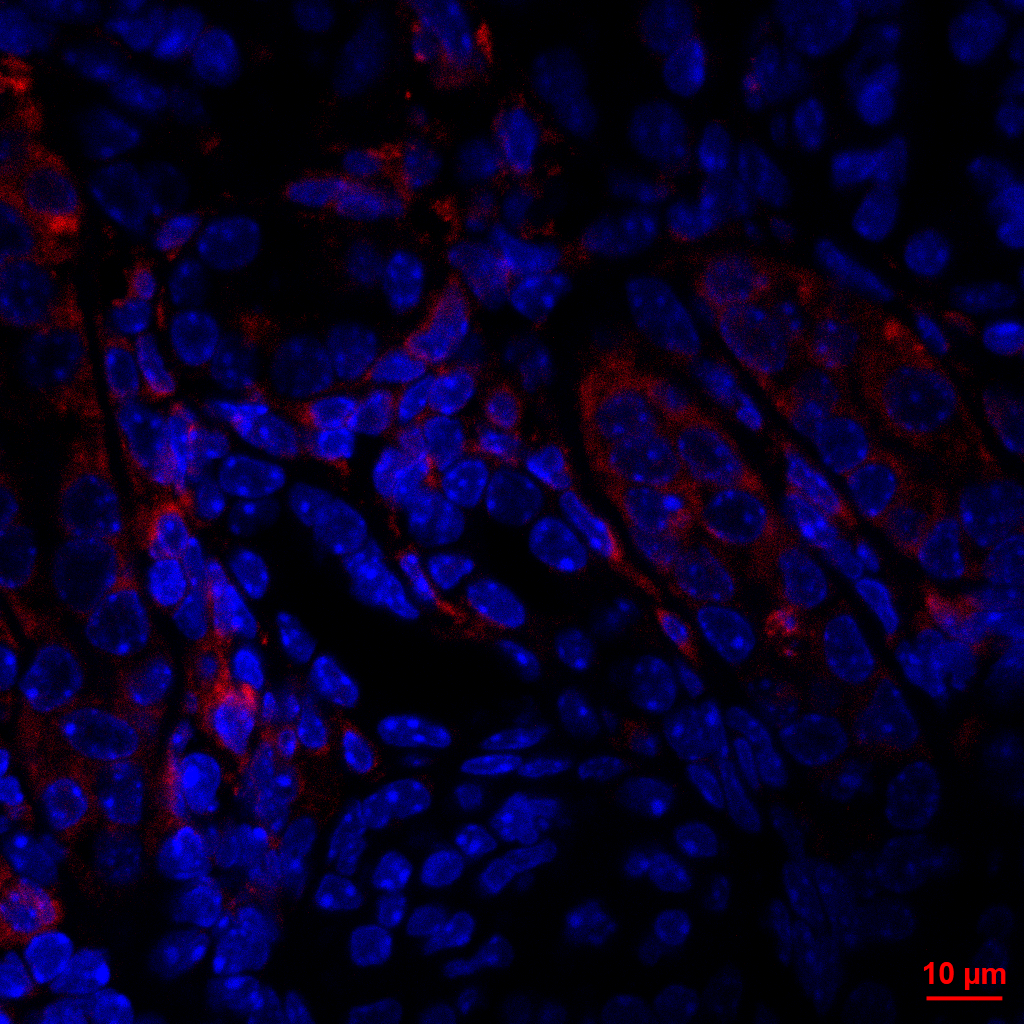

Supplement: Supplementary file 12 — Source data Fig. 7 [file 44321_2024_81_MOESM12_ESM.zip › Figure 7/7H/Aldh1a1-KO UUO.tif]

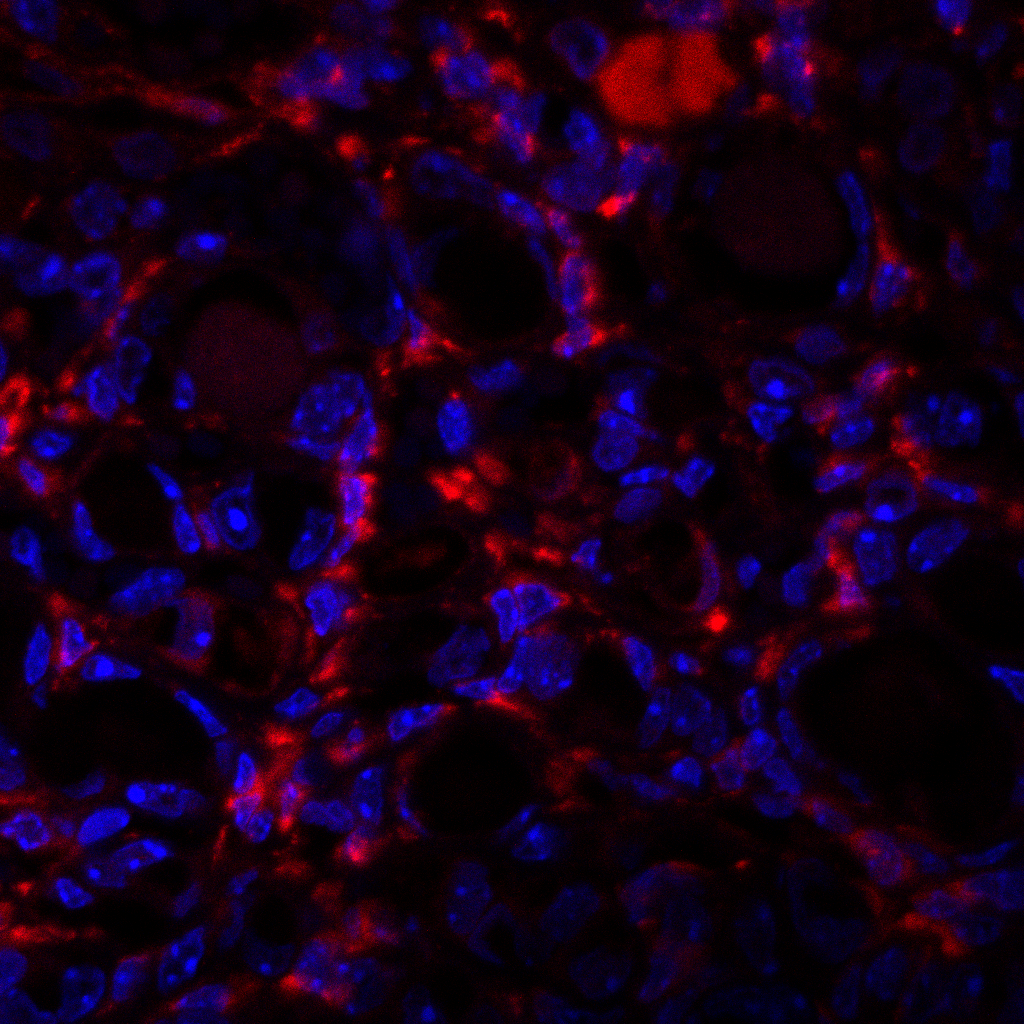

Supplement: Supplementary file 12 — Source data Fig. 7 [file 44321_2024_81_MOESM12_ESM.zip › Figure 7/7H/WT UUO.tif]

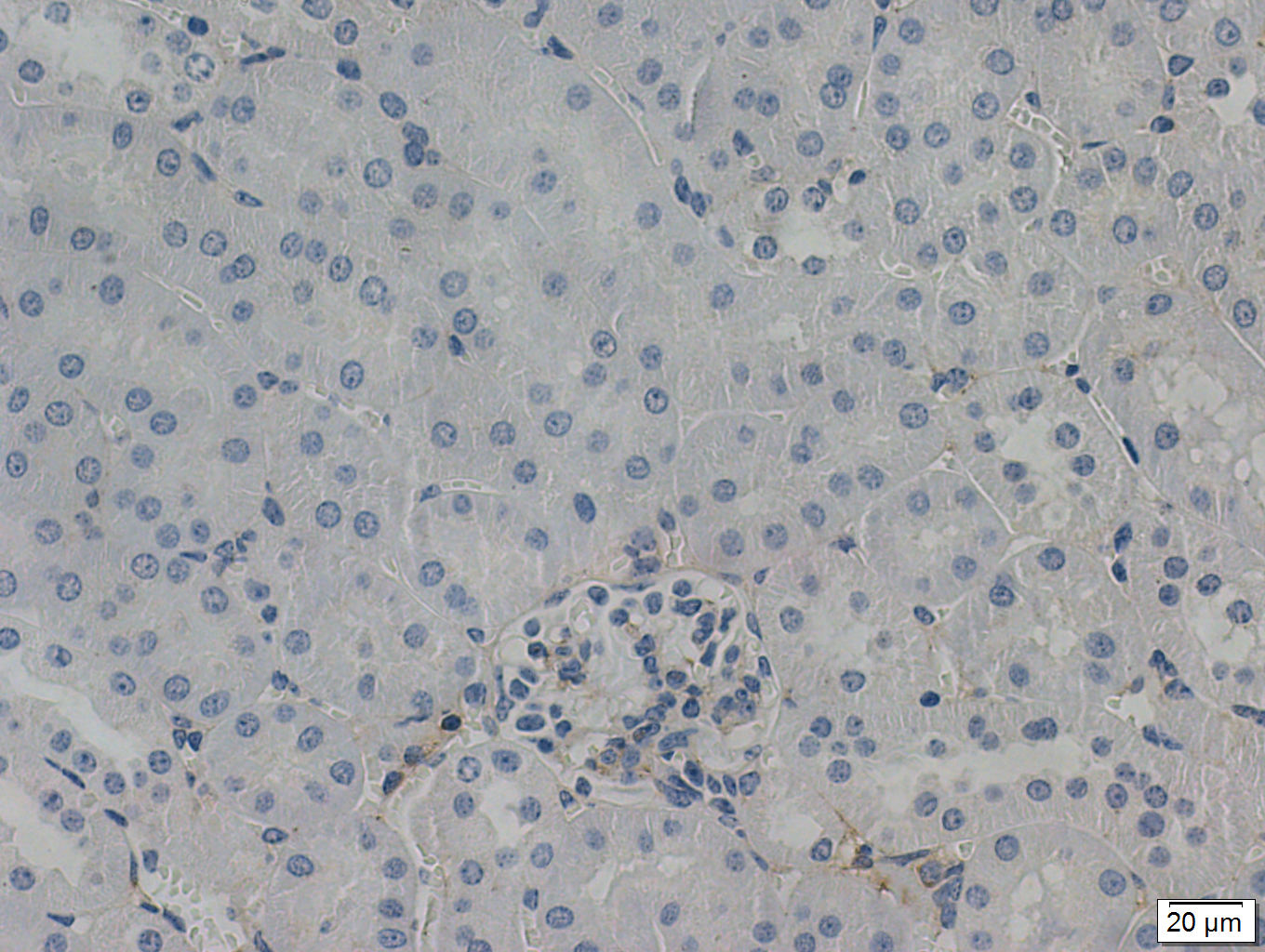

Supplement: Supplementary file 12 — Source data Fig. 7 [file 44321_2024_81_MOESM12_ESM.zip › Figure 7/7C/FN IHC/WT Sham IHC.tif]

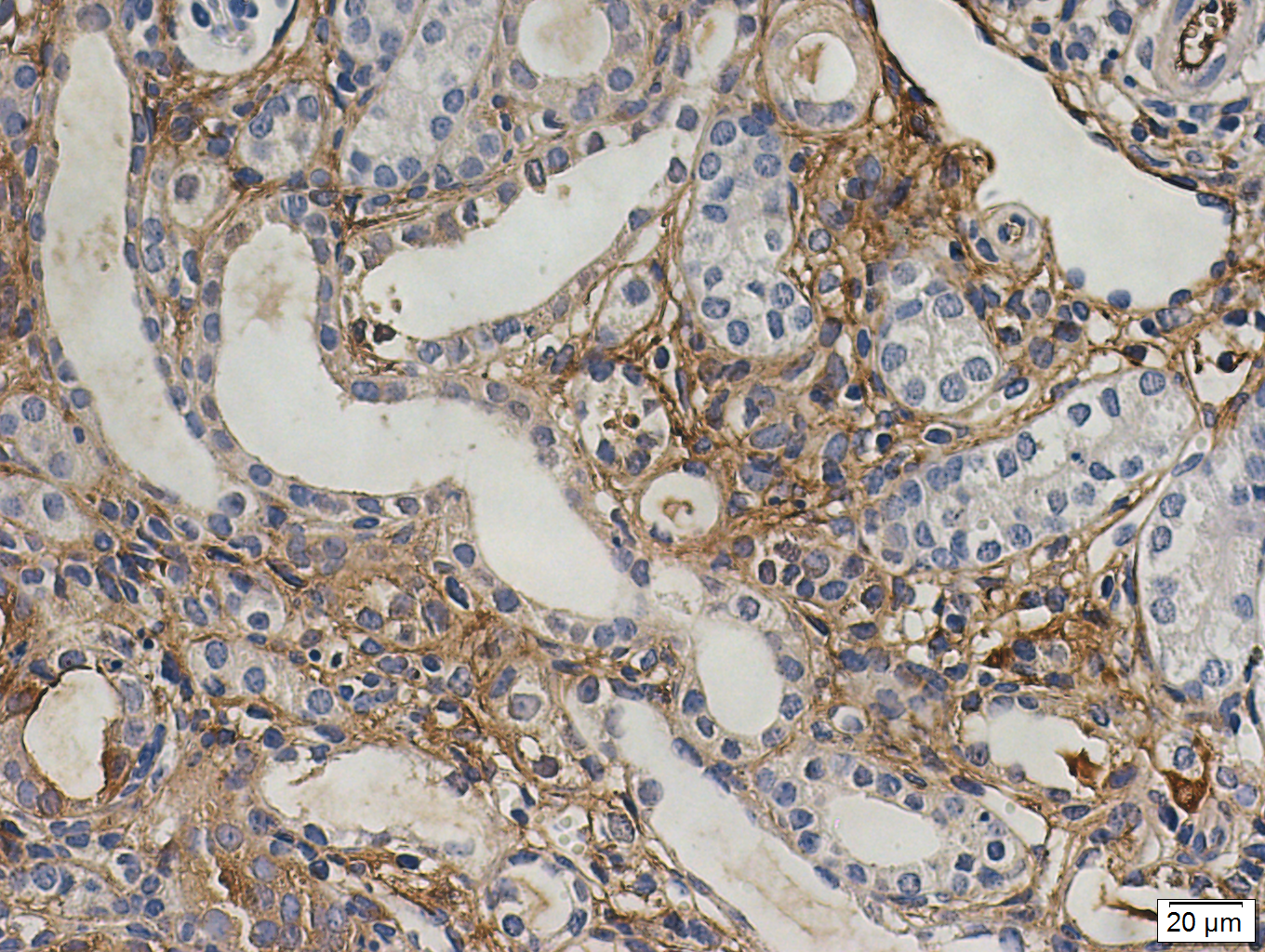

Supplement: Supplementary file 12 — Source data Fig. 7 [file 44321_2024_81_MOESM12_ESM.zip › Figure 7/7C/FN IHC/WT UUO IHC.tif]

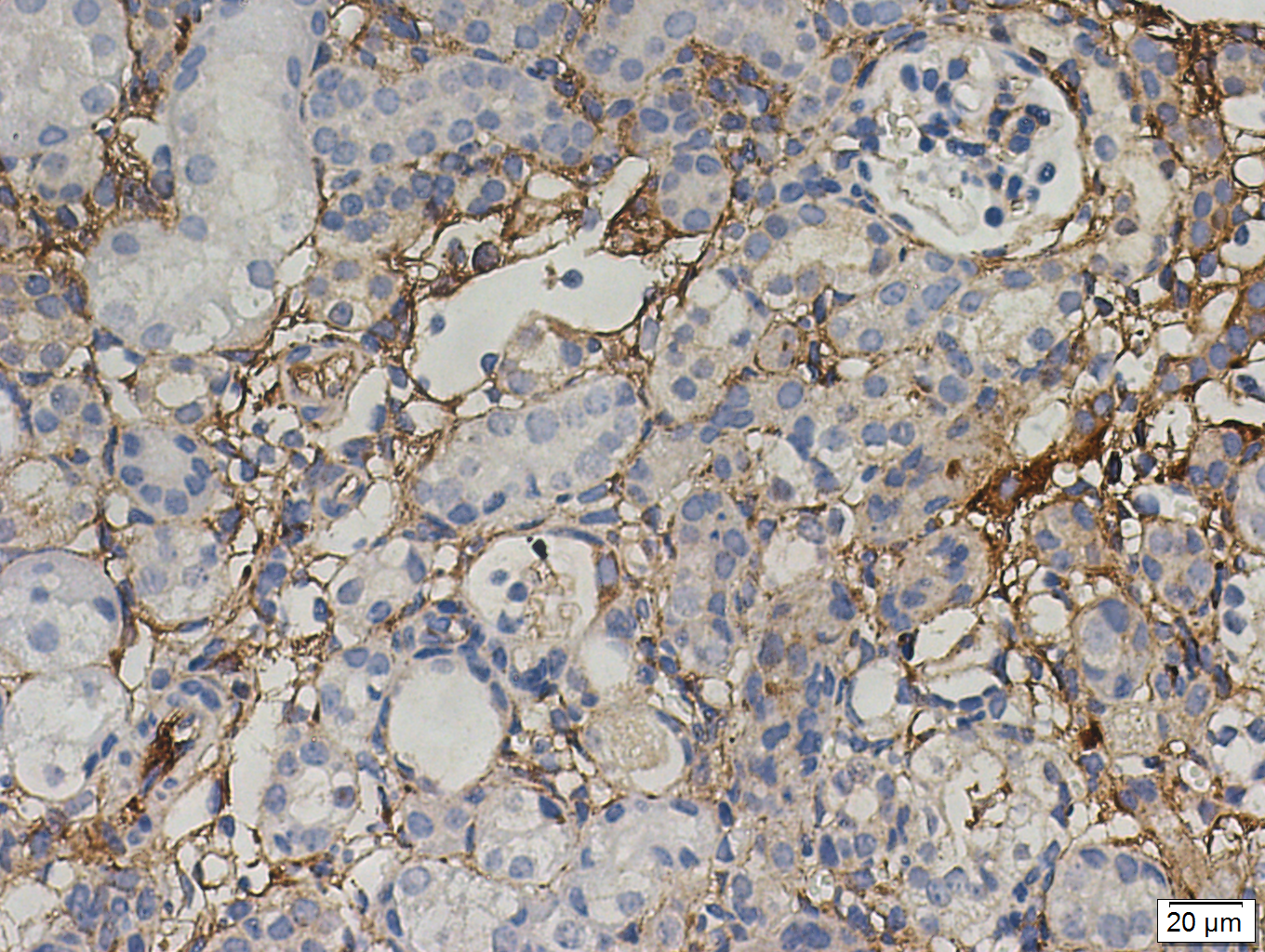

Supplement: Supplementary file 12 — Source data Fig. 7 [file 44321_2024_81_MOESM12_ESM.zip › Figure 7/7C/FN IHC/Aldh1a1-KO UUO IHC.tif]

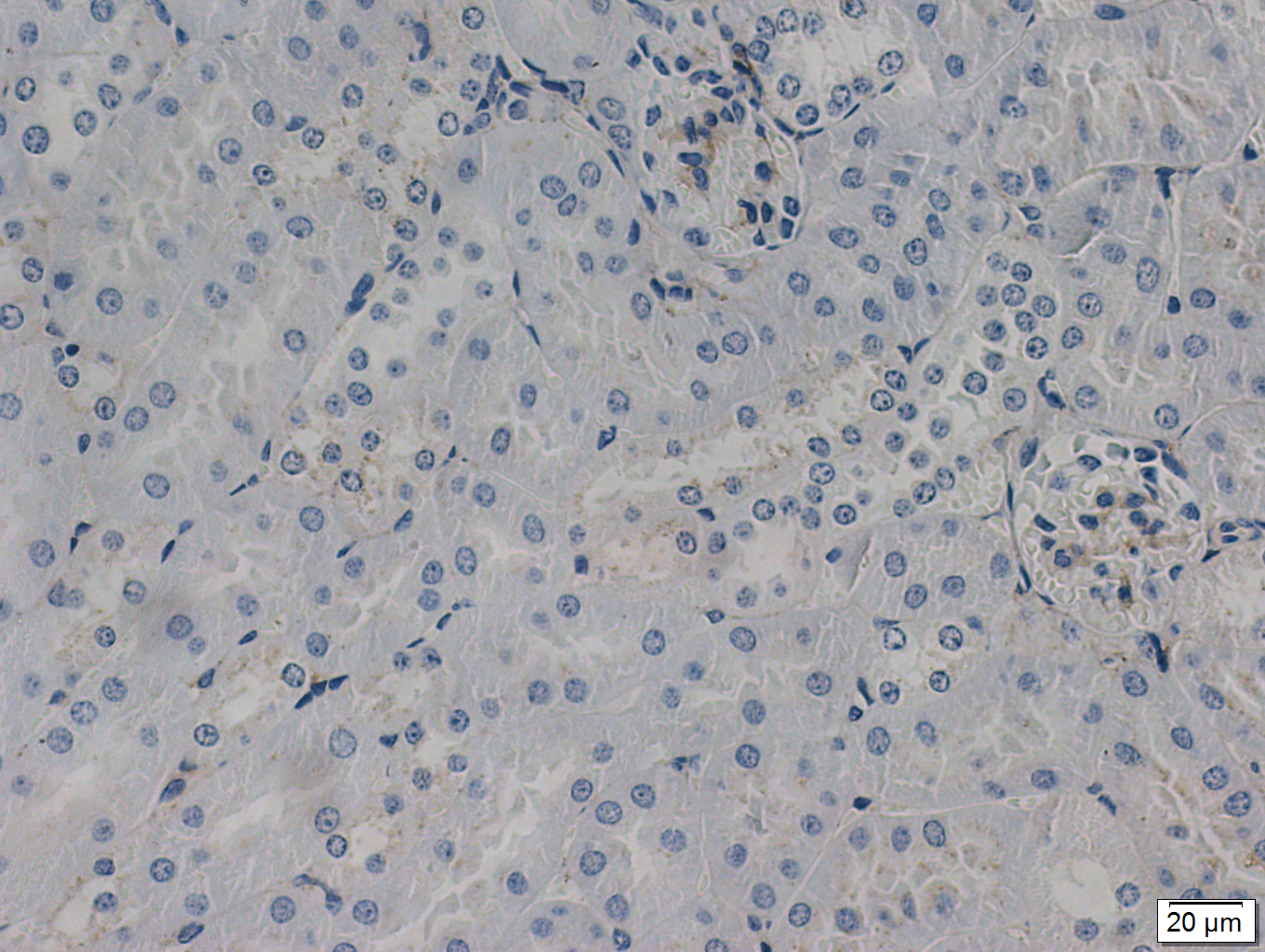

Supplement: Supplementary file 12 — Source data Fig. 7 [file 44321_2024_81_MOESM12_ESM.zip › Figure 7/7C/FN IHC/Aldh1a1-KO Sham IHC.tif]

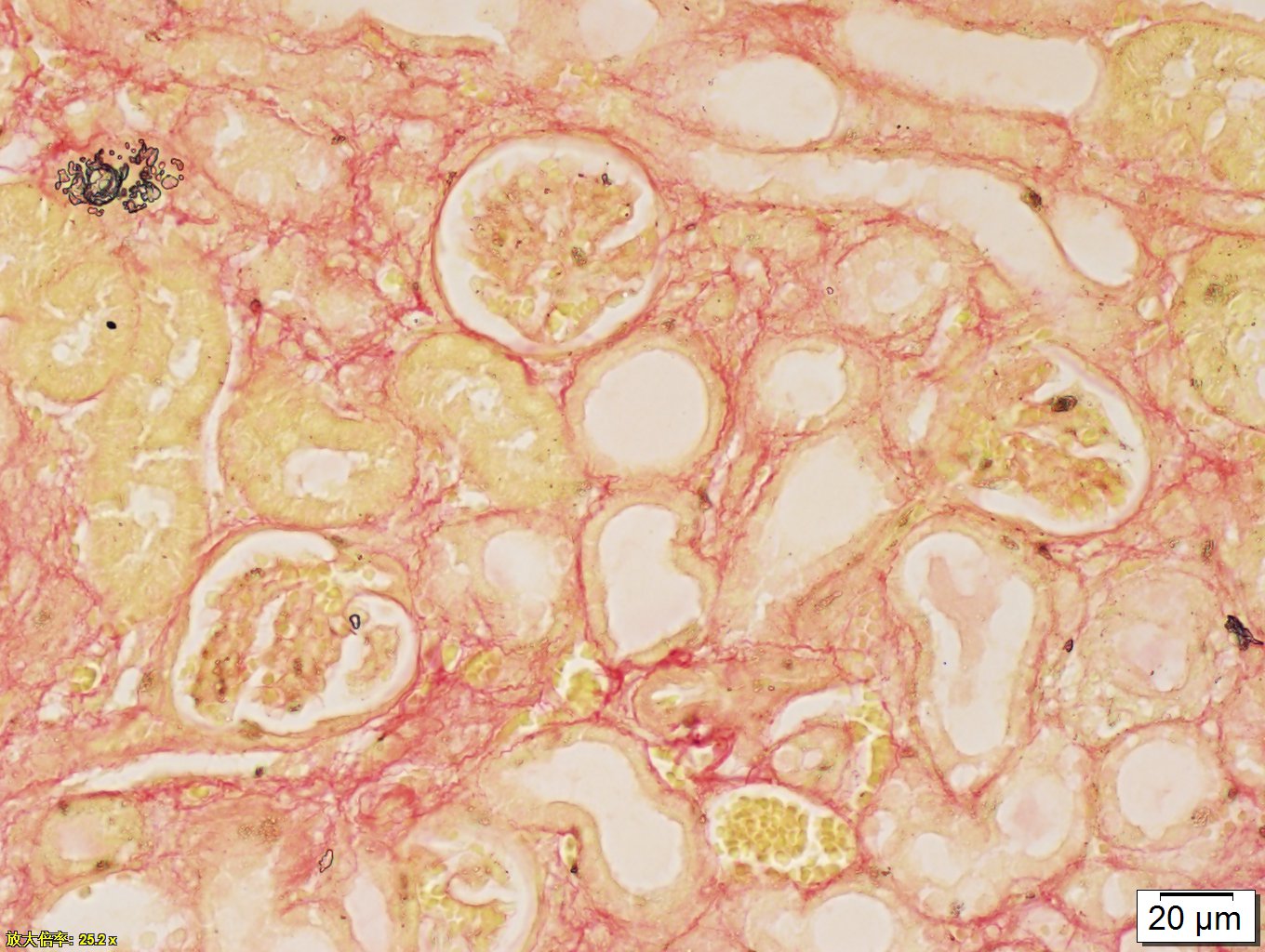

Supplement: Supplementary file 12 — Source data Fig. 7 [file 44321_2024_81_MOESM12_ESM.zip › Figure 7/7C/Sirius red/Aldh1a1-KO UUO.jpg]

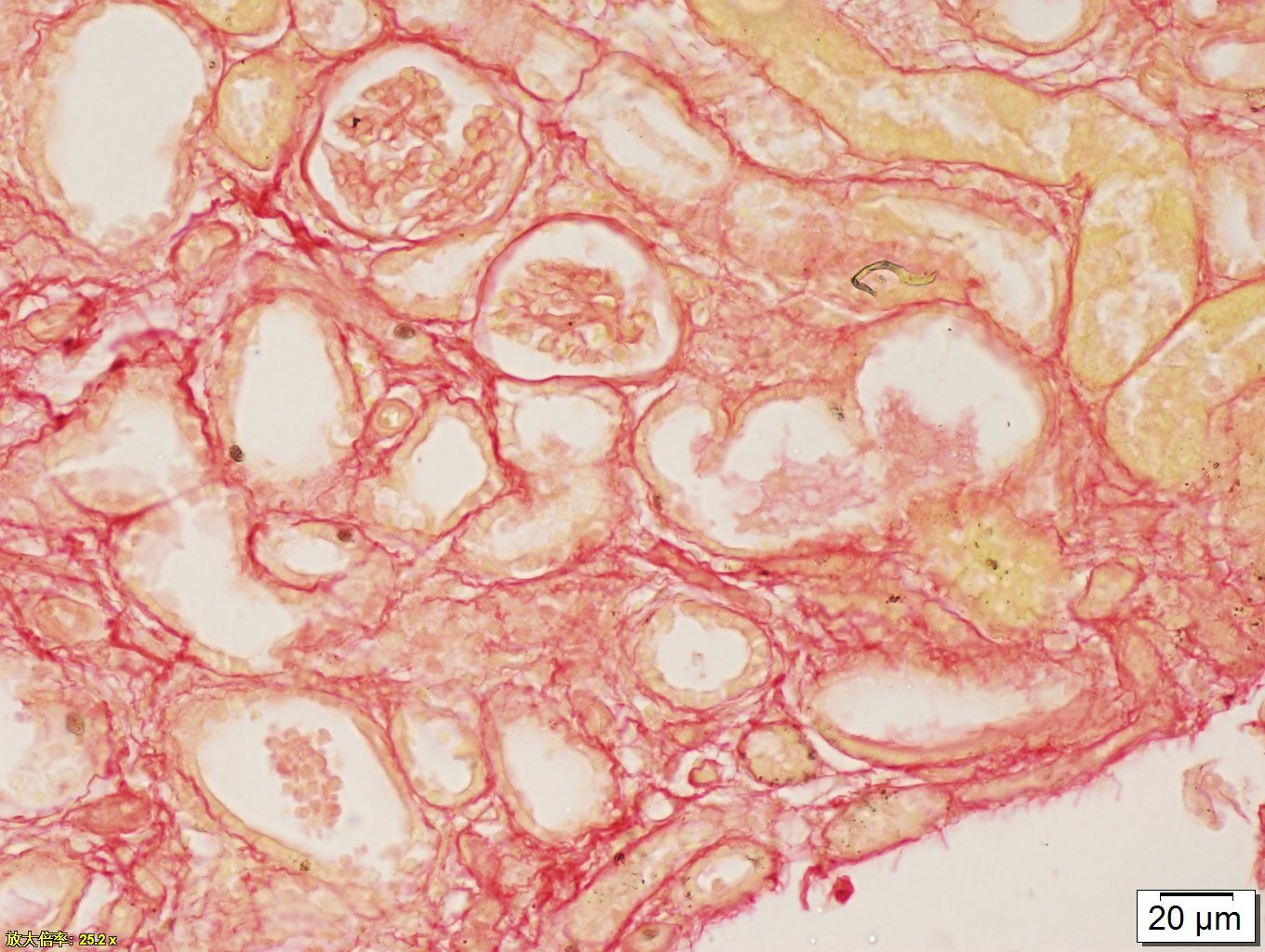

Supplement: Supplementary file 12 — Source data Fig. 7 [file 44321_2024_81_MOESM12_ESM.zip › Figure 7/7C/Sirius red/WT UUO.jpg]

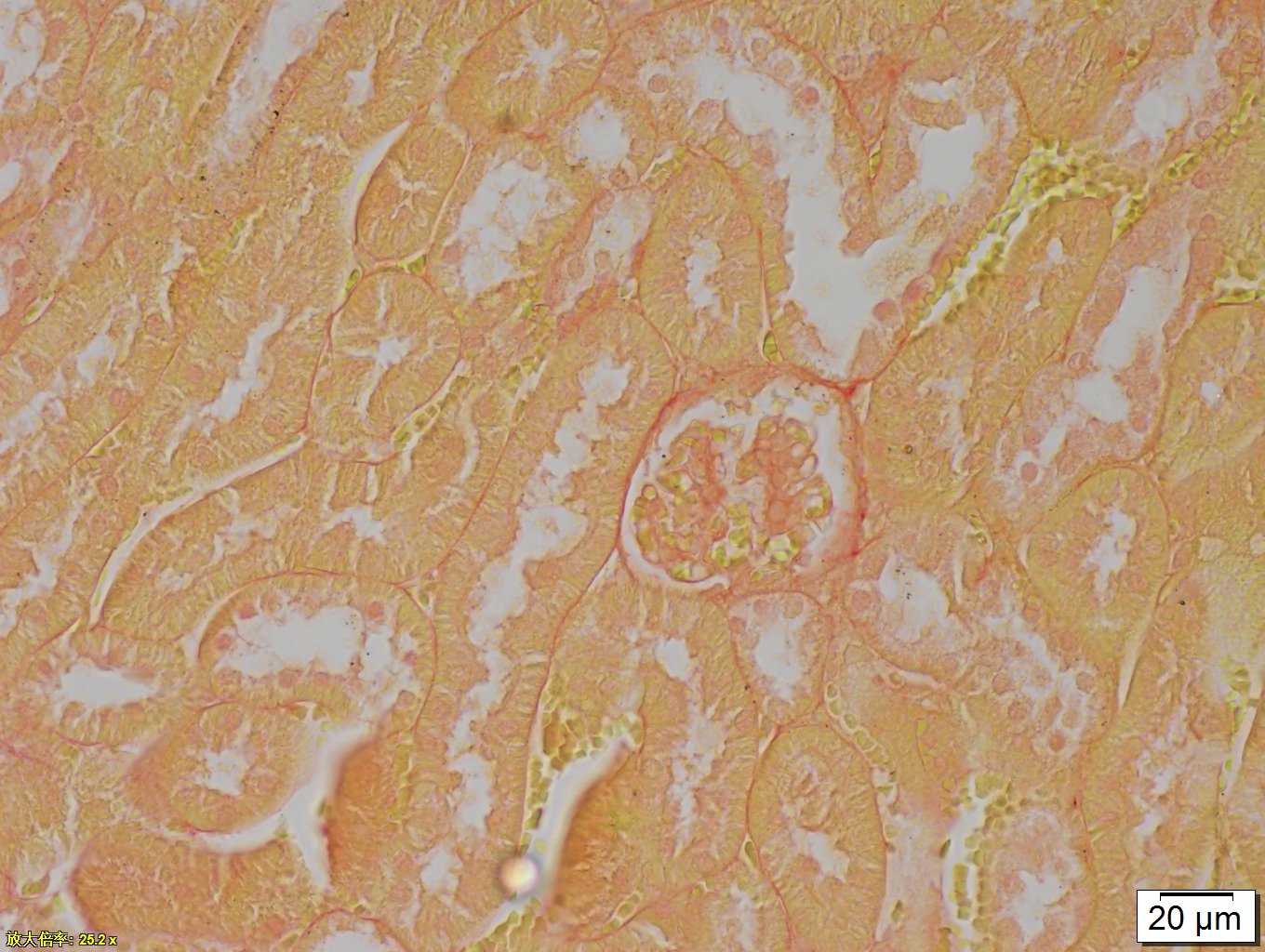

Supplement: Supplementary file 12 — Source data Fig. 7 [file 44321_2024_81_MOESM12_ESM.zip › Figure 7/7C/Sirius red/Aldh1a1-KO Sham.jpg]

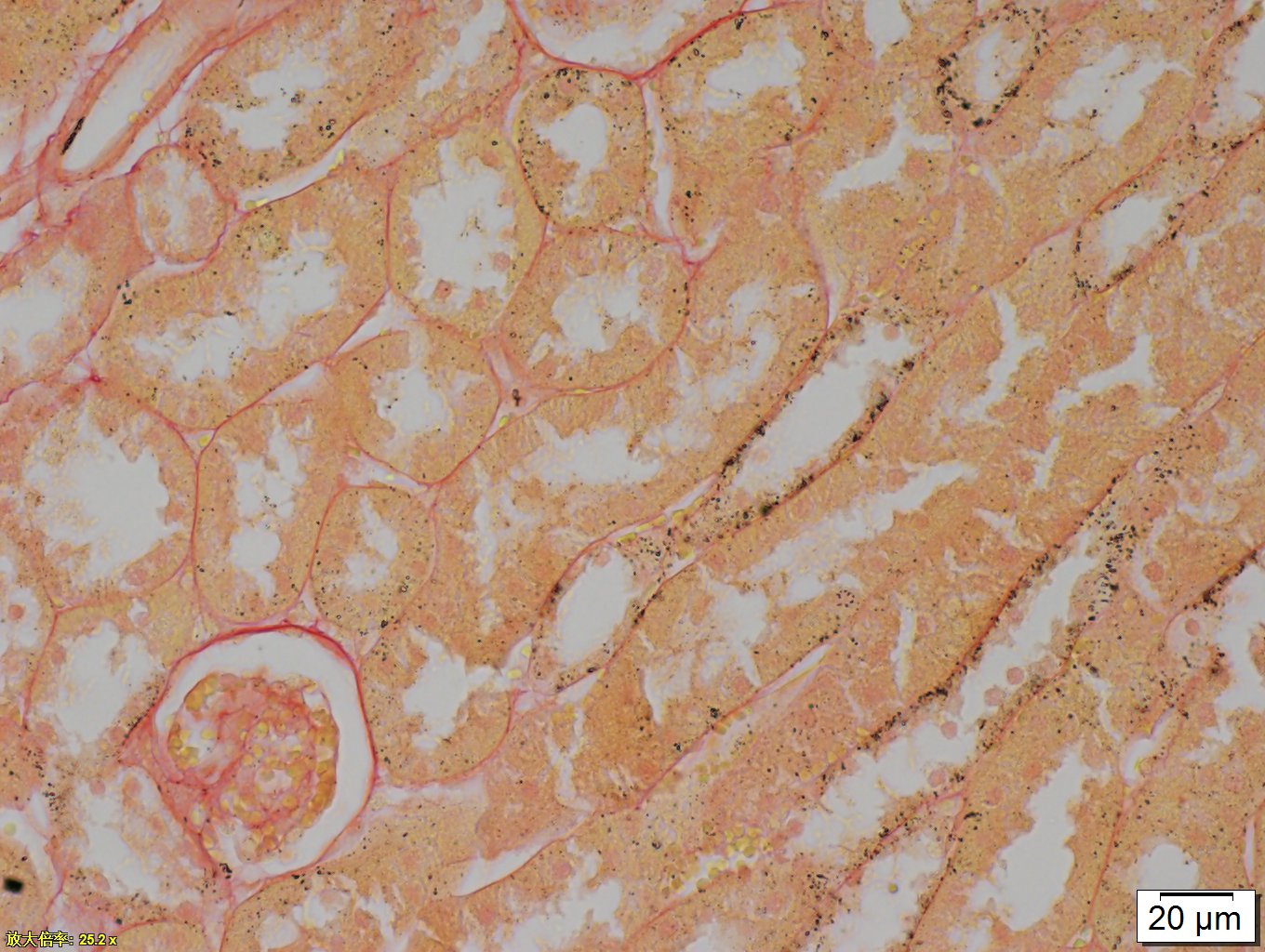

Supplement: Supplementary file 12 — Source data Fig. 7 [file 44321_2024_81_MOESM12_ESM.zip › Figure 7/7C/Sirius red/WT Sham.jpg]

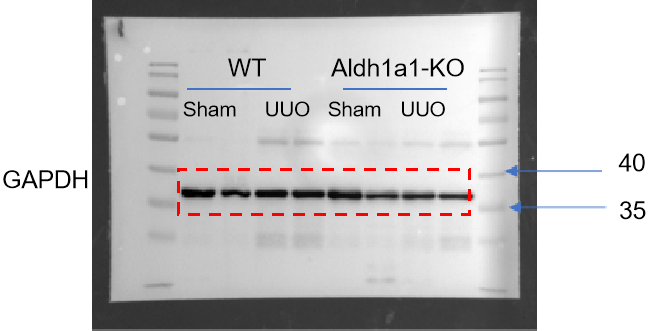

Supplement: Supplementary file 12 — Source data Fig. 7 [file 44321_2024_81_MOESM12_ESM.zip › Figure 7/7E/repeat/western GAPDH repeat.tif]

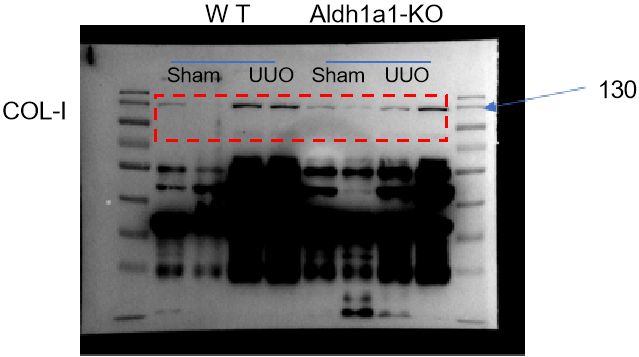

Supplement: Supplementary file 12 — Source data Fig. 7 [file 44321_2024_81_MOESM12_ESM.zip › Figure 7/7E/repeat/western COL-I repeat.tif]

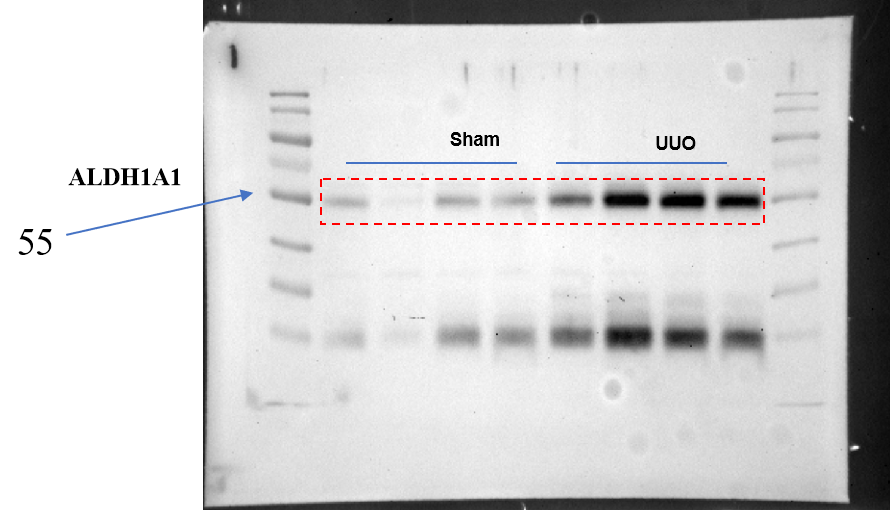

Supplement: Supplementary file 12 — Source data Fig. 7 [file 44321_2024_81_MOESM12_ESM.zip › Figure 7/7A/repeat/western ALDH1A1 repeat.tif]

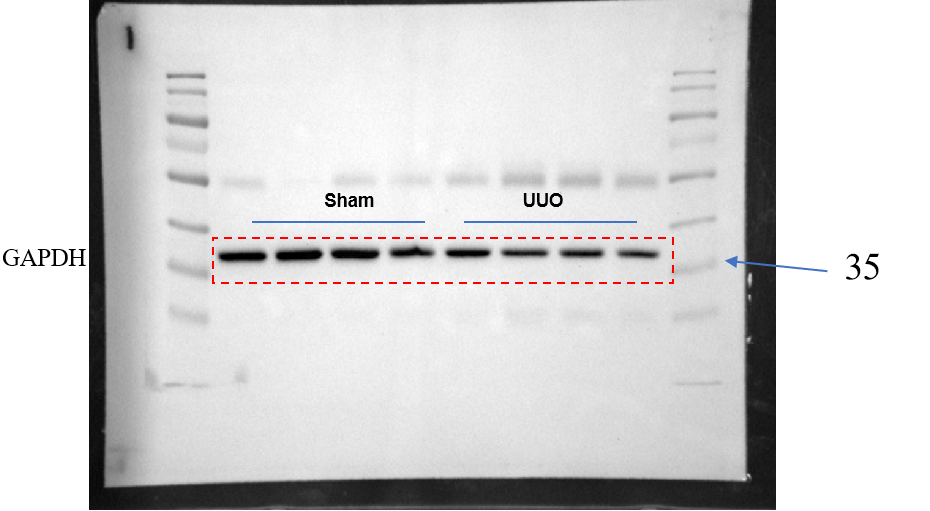

Supplement: Supplementary file 12 — Source data Fig. 7 [file 44321_2024_81_MOESM12_ESM.zip › Figure 7/7A/repeat/western GAPDH repeat.tif]

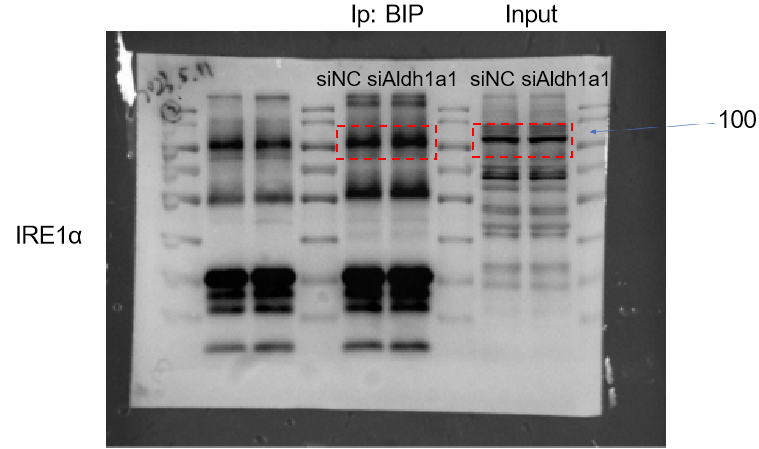

Supplement: Supplementary file 13 — Source data Fig. 8 [file 44321_2024_81_MOESM13_ESM.zip › Figure 8/8I/western IRE1╬▒.tif]

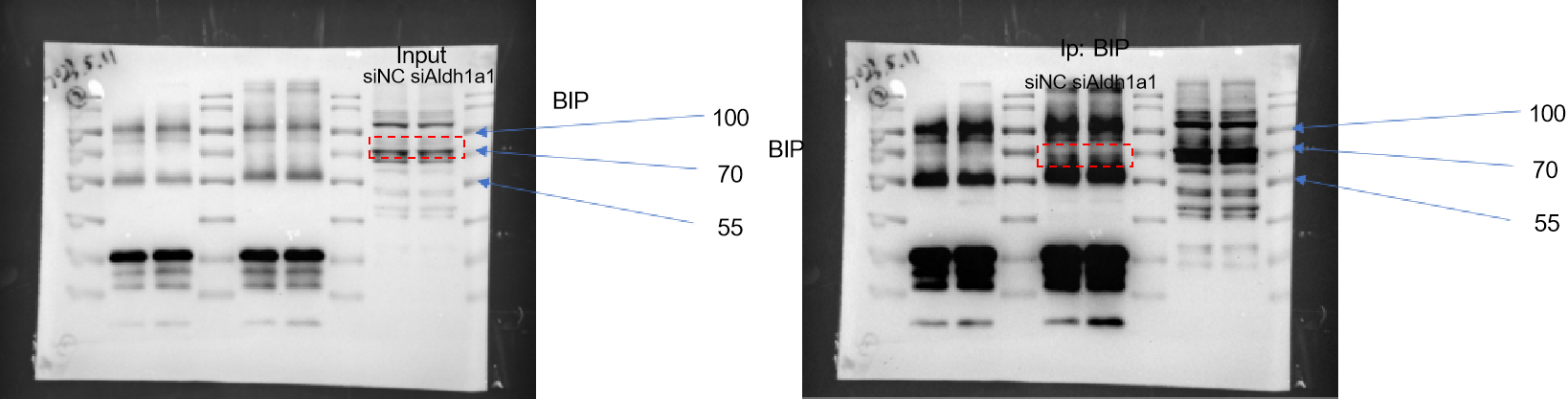

Supplement: Supplementary file 13 — Source data Fig. 8 [file 44321_2024_81_MOESM13_ESM.zip › Figure 8/8I/western BIP.tif]

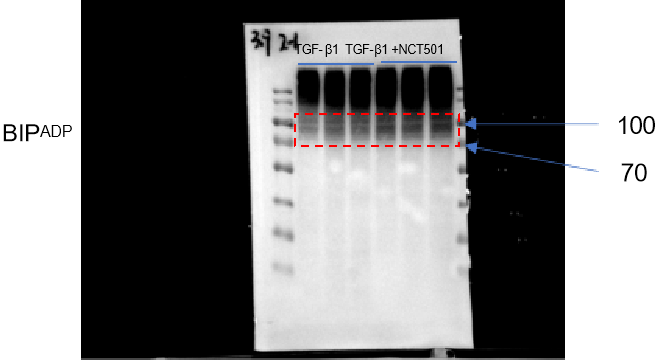

Supplement: Supplementary file 13 — Source data Fig. 8 [file 44321_2024_81_MOESM13_ESM.zip › Figure 8/8G/western BIPADP.tif]

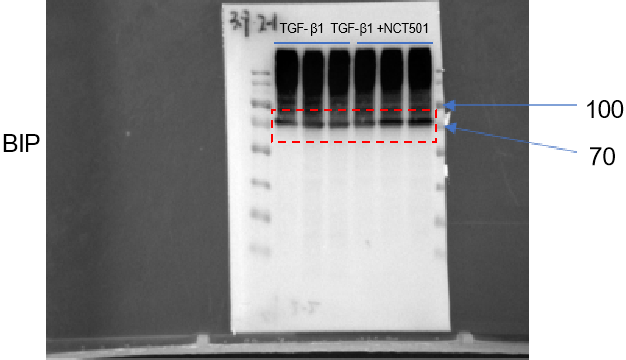

Supplement: Supplementary file 13 — Source data Fig. 8 [file 44321_2024_81_MOESM13_ESM.zip › Figure 8/8G/western BIP.tif]

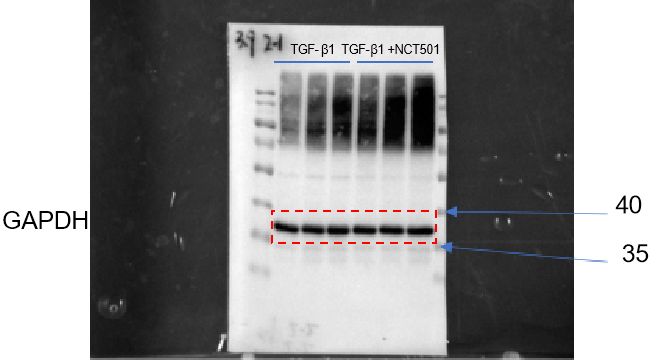

Supplement: Supplementary file 13 — Source data Fig. 8 [file 44321_2024_81_MOESM13_ESM.zip › Figure 8/8G/western GAPDH.tif]

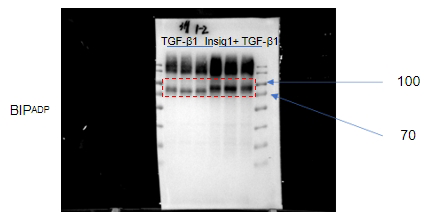

Supplement: Supplementary file 13 — Source data Fig. 8 [file 44321_2024_81_MOESM13_ESM.zip › Figure 8/8F/western BIPADP.tif]

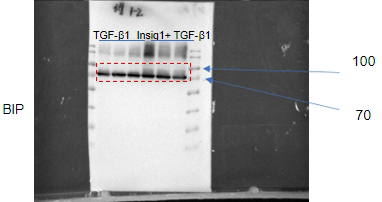

Supplement: Supplementary file 13 — Source data Fig. 8 [file 44321_2024_81_MOESM13_ESM.zip › Figure 8/8F/western BIP.tif]

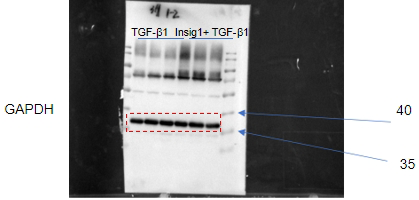

Supplement: Supplementary file 13 — Source data Fig. 8 [file 44321_2024_81_MOESM13_ESM.zip › Figure 8/8F/western GAPDH.tif]

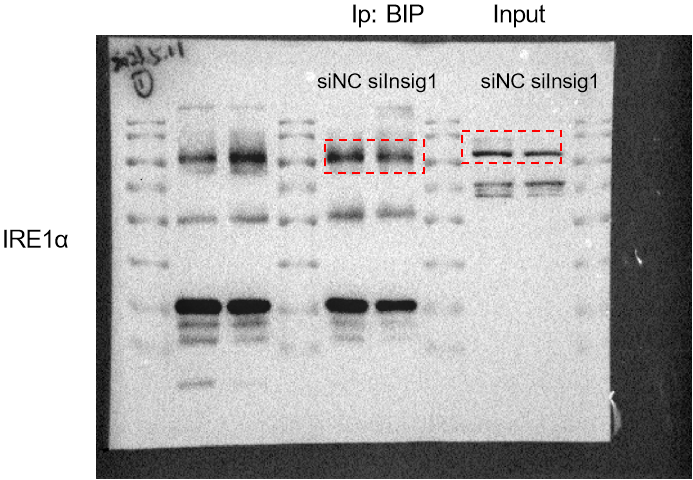

Supplement: Supplementary file 13 — Source data Fig. 8 [file 44321_2024_81_MOESM13_ESM.zip › Figure 8/8H/western IRE1╬▒.tif]

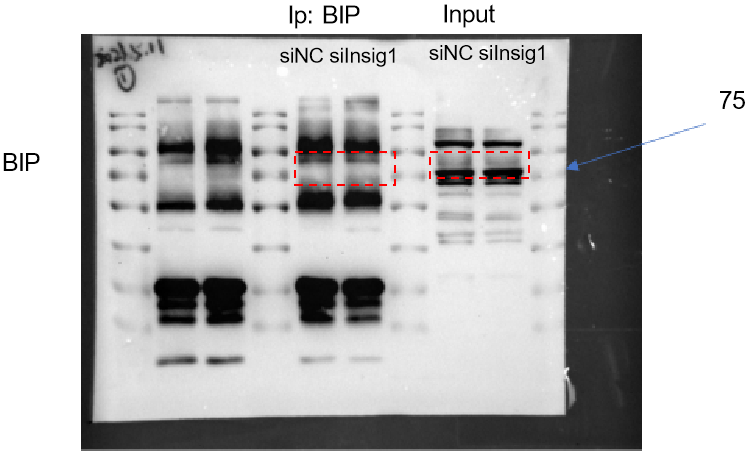

Supplement: Supplementary file 13 — Source data Fig. 8 [file 44321_2024_81_MOESM13_ESM.zip › Figure 8/8H/western BIP.tif]

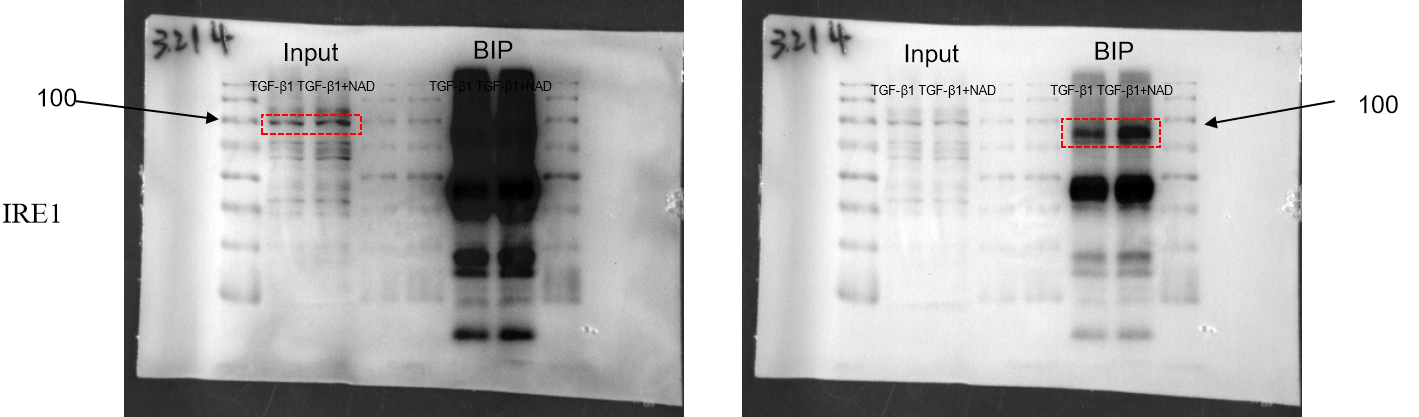

Supplement: Supplementary file 13 — Source data Fig. 8 [file 44321_2024_81_MOESM13_ESM.zip › Figure 8/8J/western IRE1╬▒.tif]

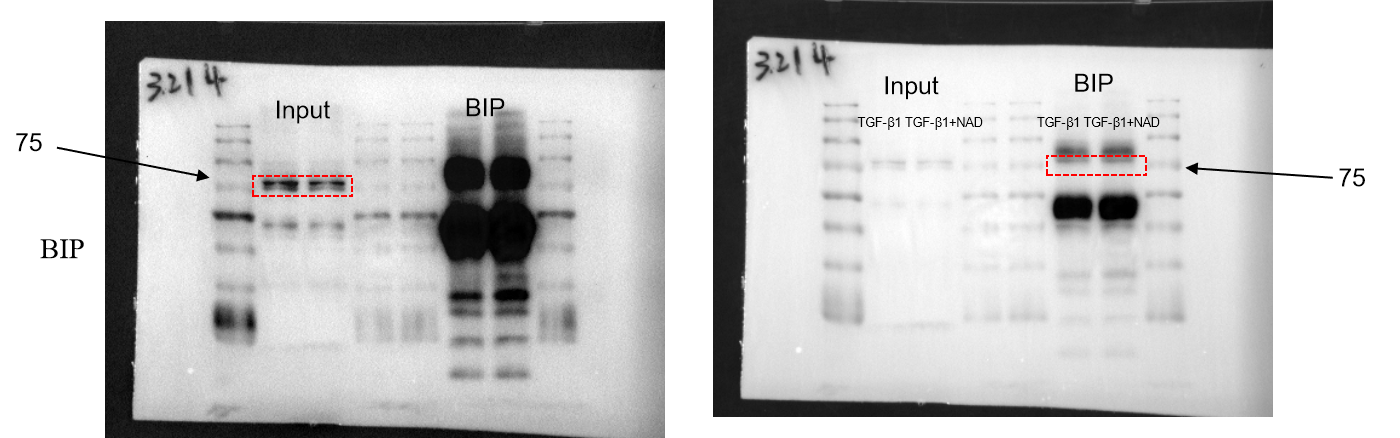

Supplement: Supplementary file 13 — Source data Fig. 8 [file 44321_2024_81_MOESM13_ESM.zip › Figure 8/8J/western BIP.tif]

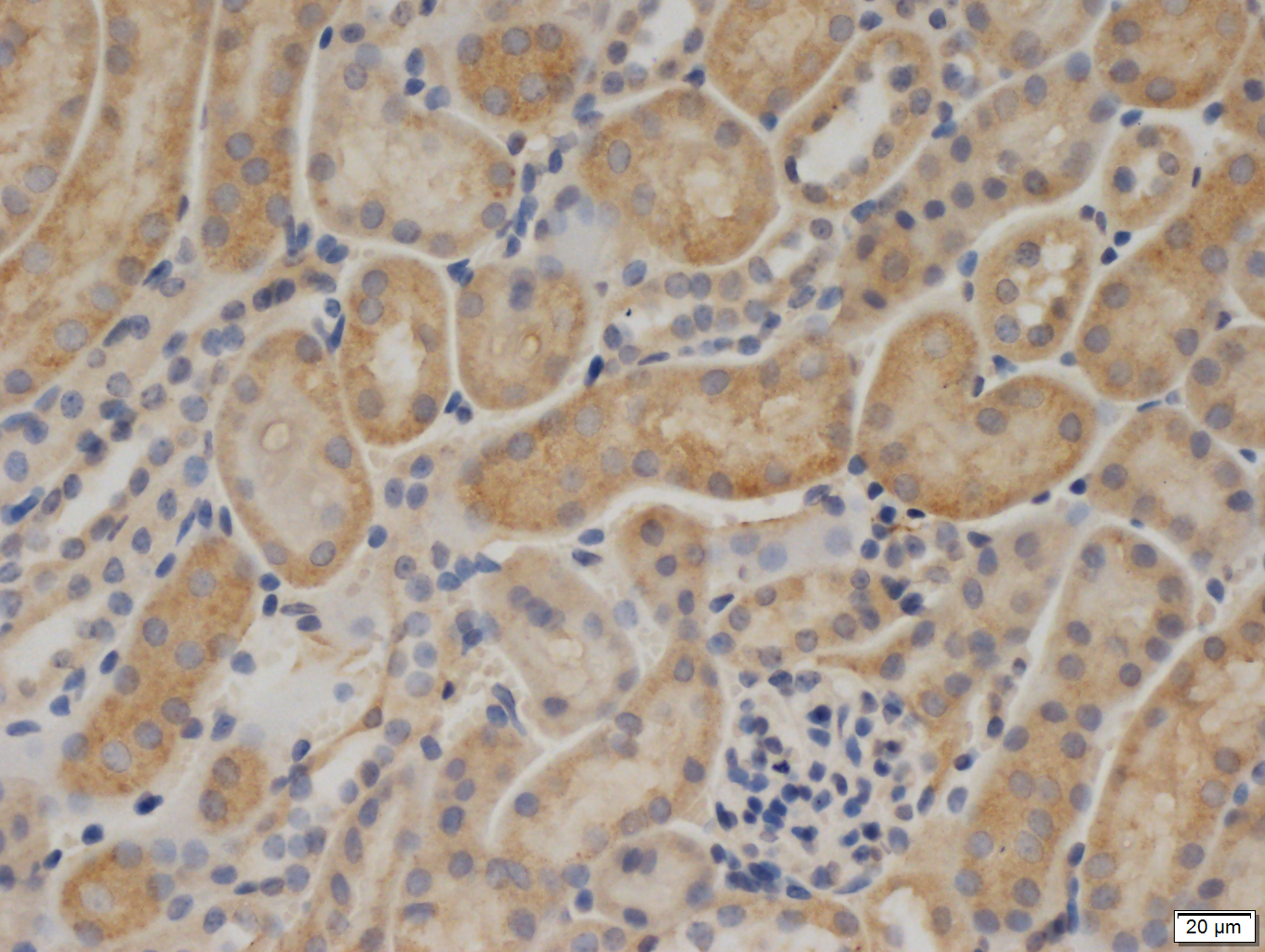

Supplement: Supplementary file 14 — Source data Fig. 9 [file 44321_2024_81_MOESM14_ESM.zip › Figure 9/9E/Sham.tif]

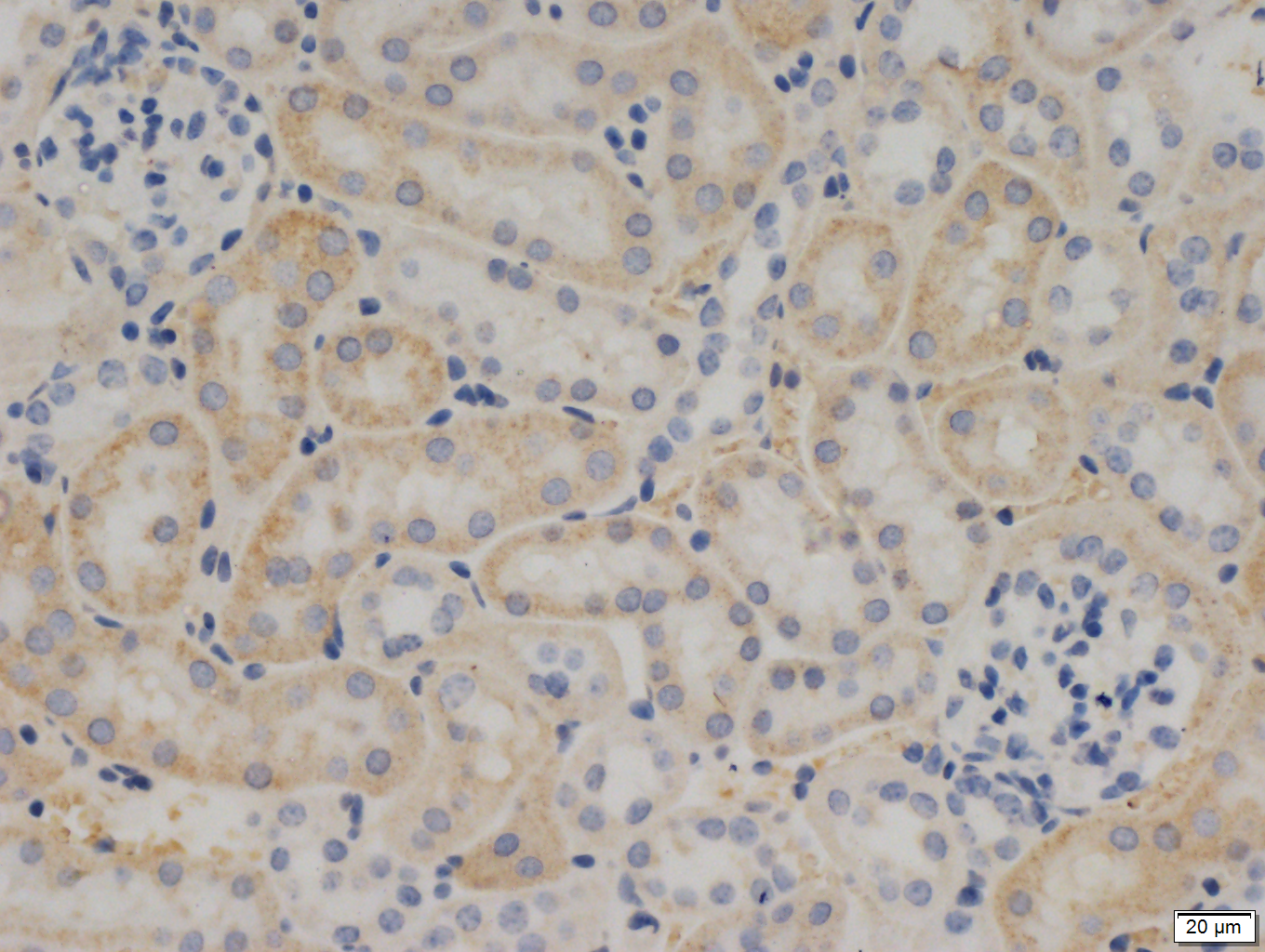

Supplement: Supplementary file 14 — Source data Fig. 9 [file 44321_2024_81_MOESM14_ESM.zip › Figure 9/9E/Nicardipine.tif]

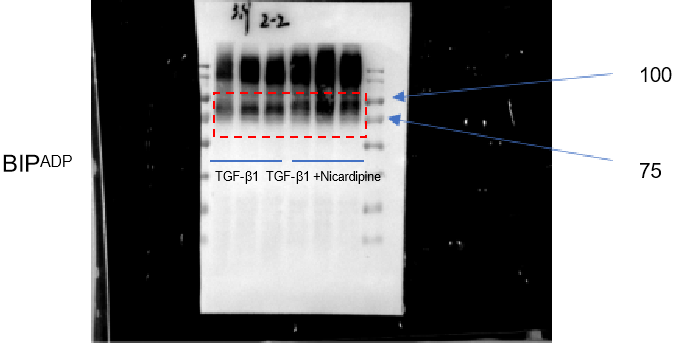

Supplement: Supplementary file 14 — Source data Fig. 9 [file 44321_2024_81_MOESM14_ESM.zip › Figure 9/9M/western BIPADP.tif]

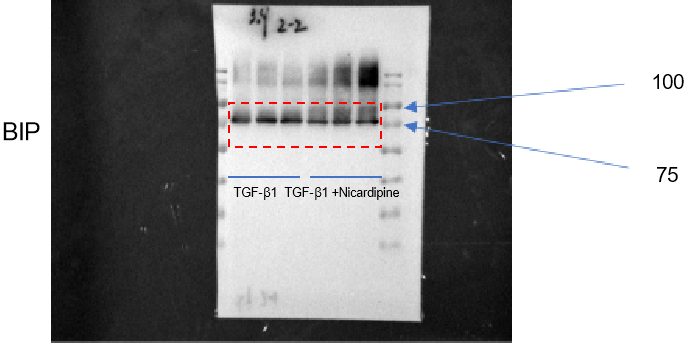

Supplement: Supplementary file 14 — Source data Fig. 9 [file 44321_2024_81_MOESM14_ESM.zip › Figure 9/9M/western BIP.tif]

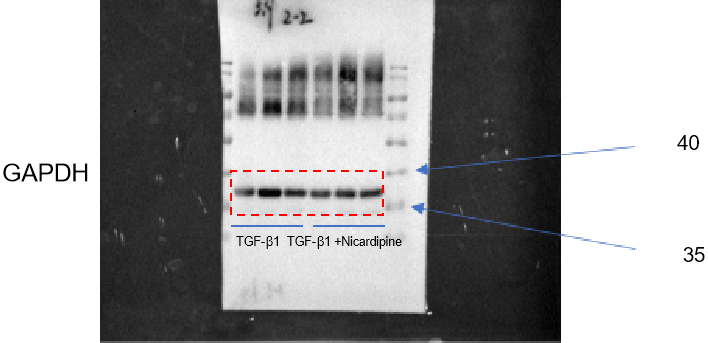

Supplement: Supplementary file 14 — Source data Fig. 9 [file 44321_2024_81_MOESM14_ESM.zip › Figure 9/9M/western GAPDH.tif]

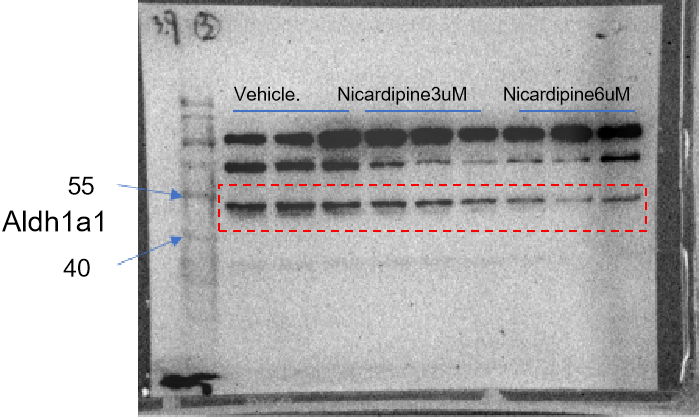

Supplement: Supplementary file 14 — Source data Fig. 9 [file 44321_2024_81_MOESM14_ESM.zip › Figure 9/9F/western ALDH1A1.tif]

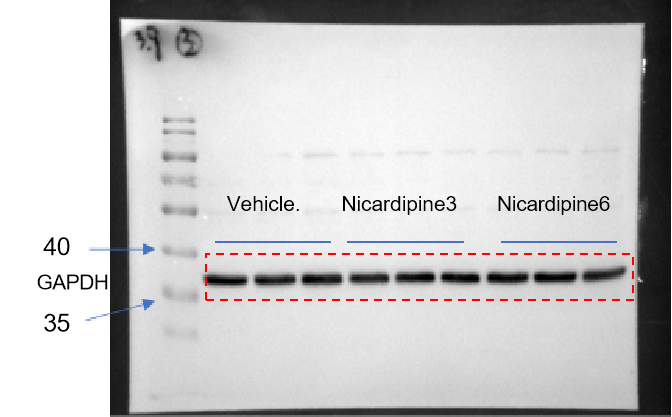

Supplement: Supplementary file 14 — Source data Fig. 9 [file 44321_2024_81_MOESM14_ESM.zip › Figure 9/9F/western GAPDH.tif]

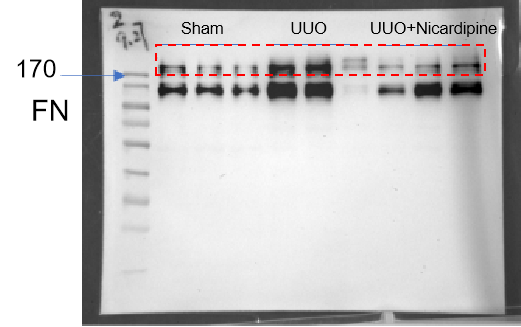

Supplement: Supplementary file 14 — Source data Fig. 9 [file 44321_2024_81_MOESM14_ESM.zip › Figure 9/9H/western FN.tif]

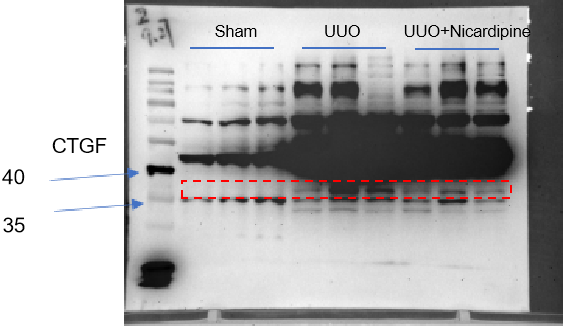

Supplement: Supplementary file 14 — Source data Fig. 9 [file 44321_2024_81_MOESM14_ESM.zip › Figure 9/9H/western CTGF.tif]

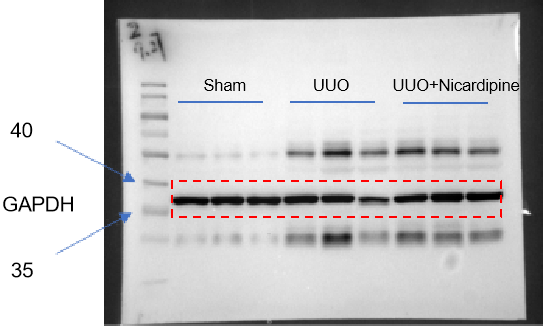

Supplement: Supplementary file 14 — Source data Fig. 9 [file 44321_2024_81_MOESM14_ESM.zip › Figure 9/9H/western GAPDH.tif]

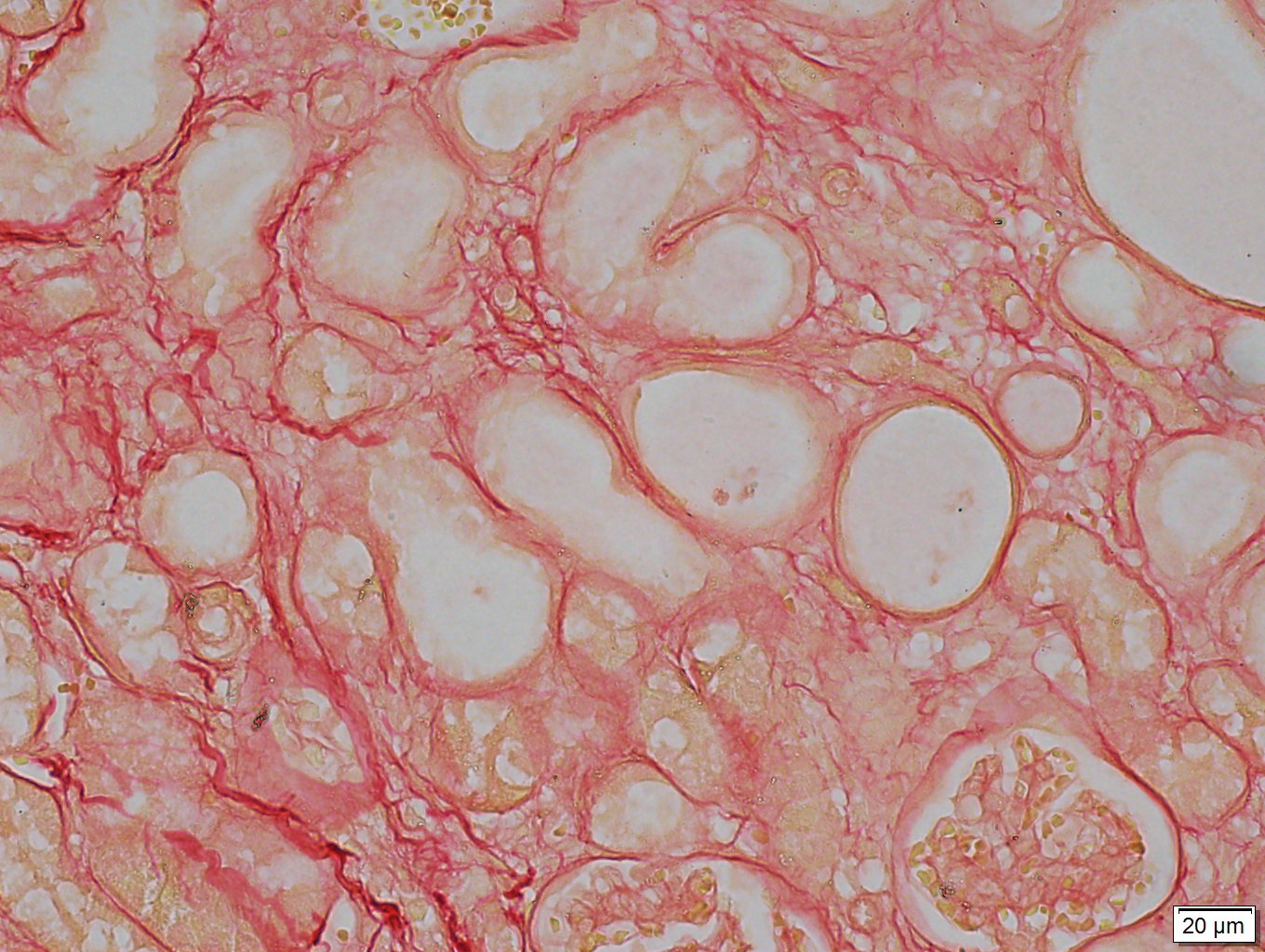

Supplement: Supplementary file 14 — Source data Fig. 9 [file 44321_2024_81_MOESM14_ESM.zip › Figure 9/9O/UUO+Nicardipine.tif]

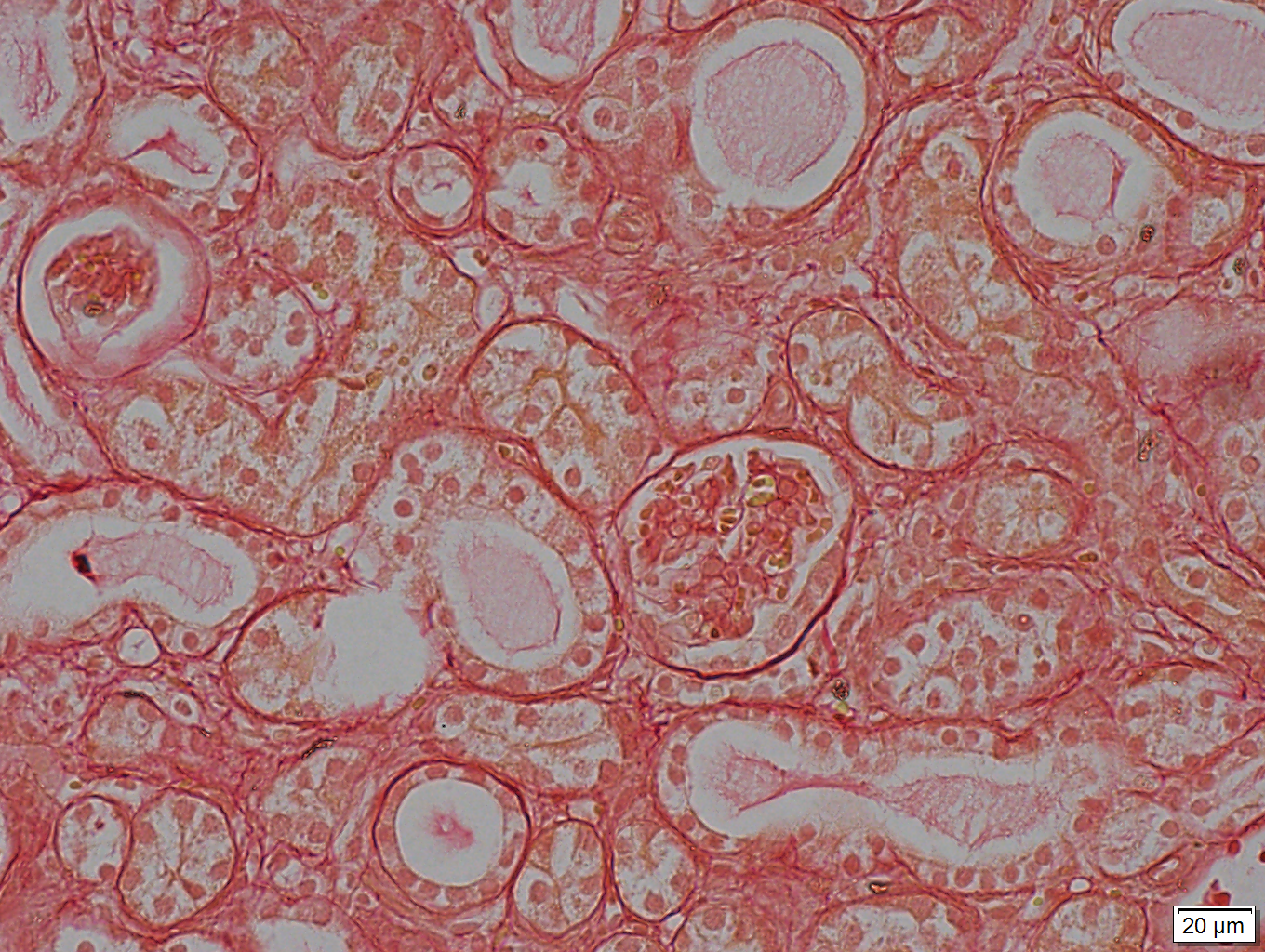

Supplement: Supplementary file 14 — Source data Fig. 9 [file 44321_2024_81_MOESM14_ESM.zip › Figure 9/9O/UUO.tif]

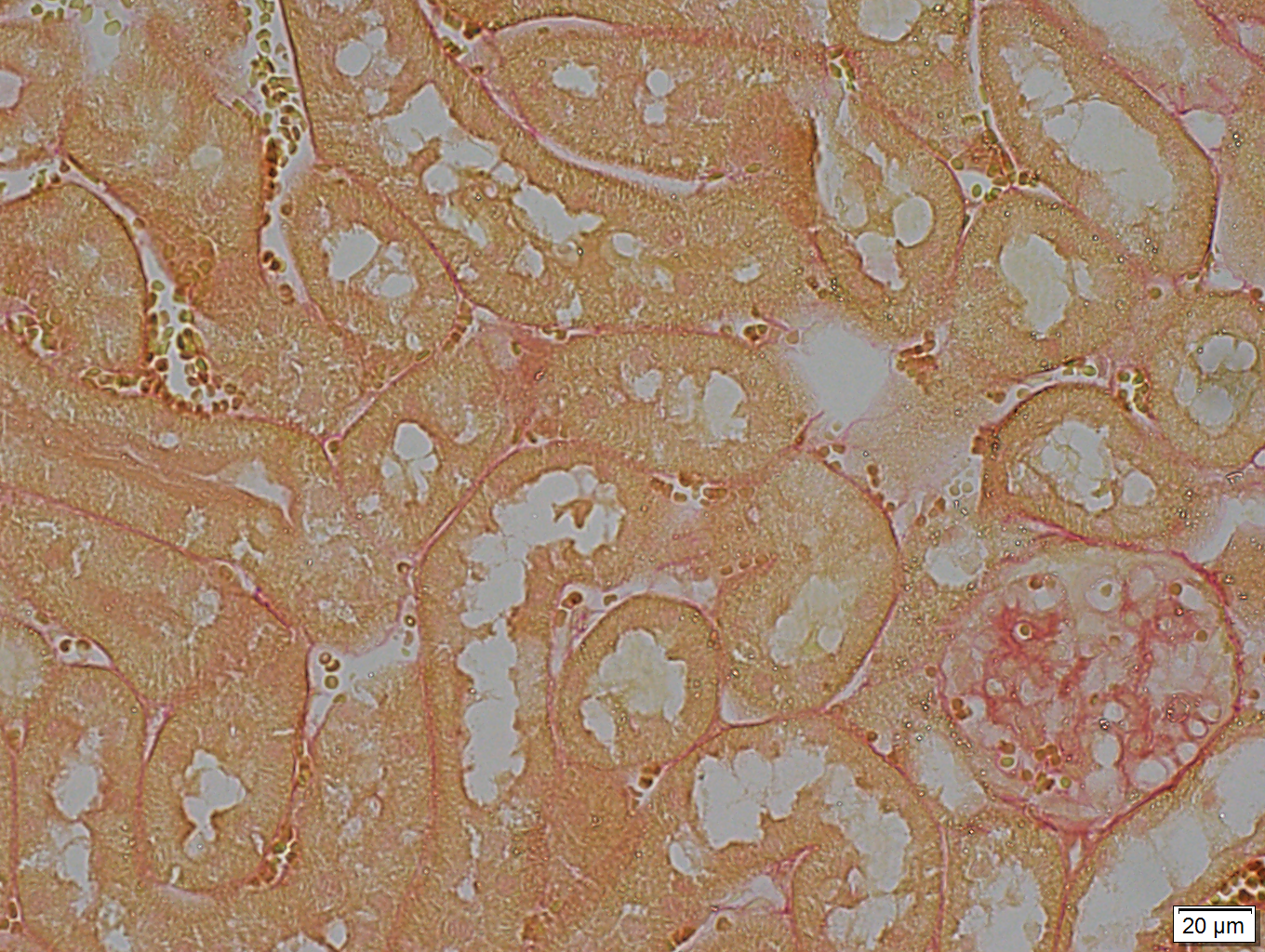

Supplement: Supplementary file 14 — Source data Fig. 9 [file 44321_2024_81_MOESM14_ESM.zip › Figure 9/9O/Sham .tif]

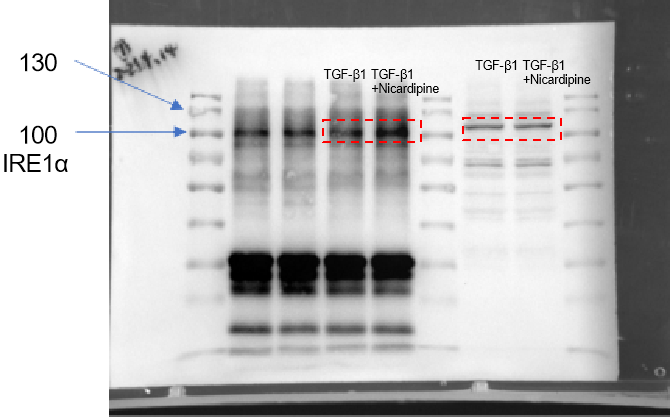

Supplement: Supplementary file 14 — Source data Fig. 9 [file 44321_2024_81_MOESM14_ESM.zip › Figure 9/9N/western IRE1╬▒.tif]

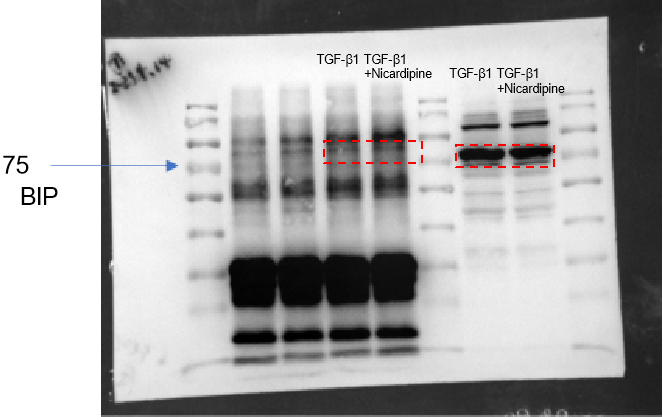

Supplement: Supplementary file 14 — Source data Fig. 9 [file 44321_2024_81_MOESM14_ESM.zip › Figure 9/9N/western BIP.tif]

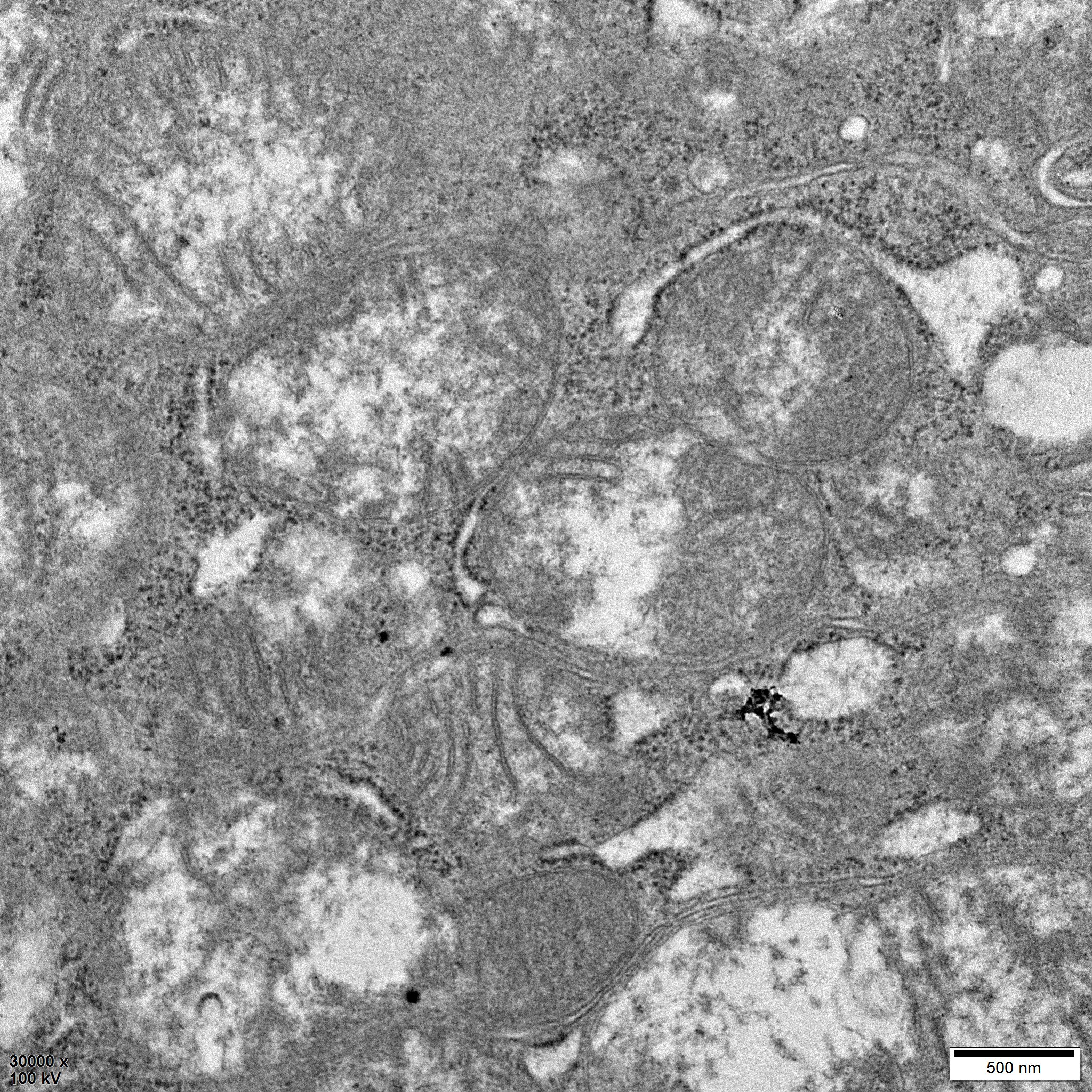

Supplement: Supplementary file 14 — Source data Fig. 9 [file 44321_2024_81_MOESM14_ESM.zip › Figure 9/9J/TEM/UUO+Nicardipine.jpg]

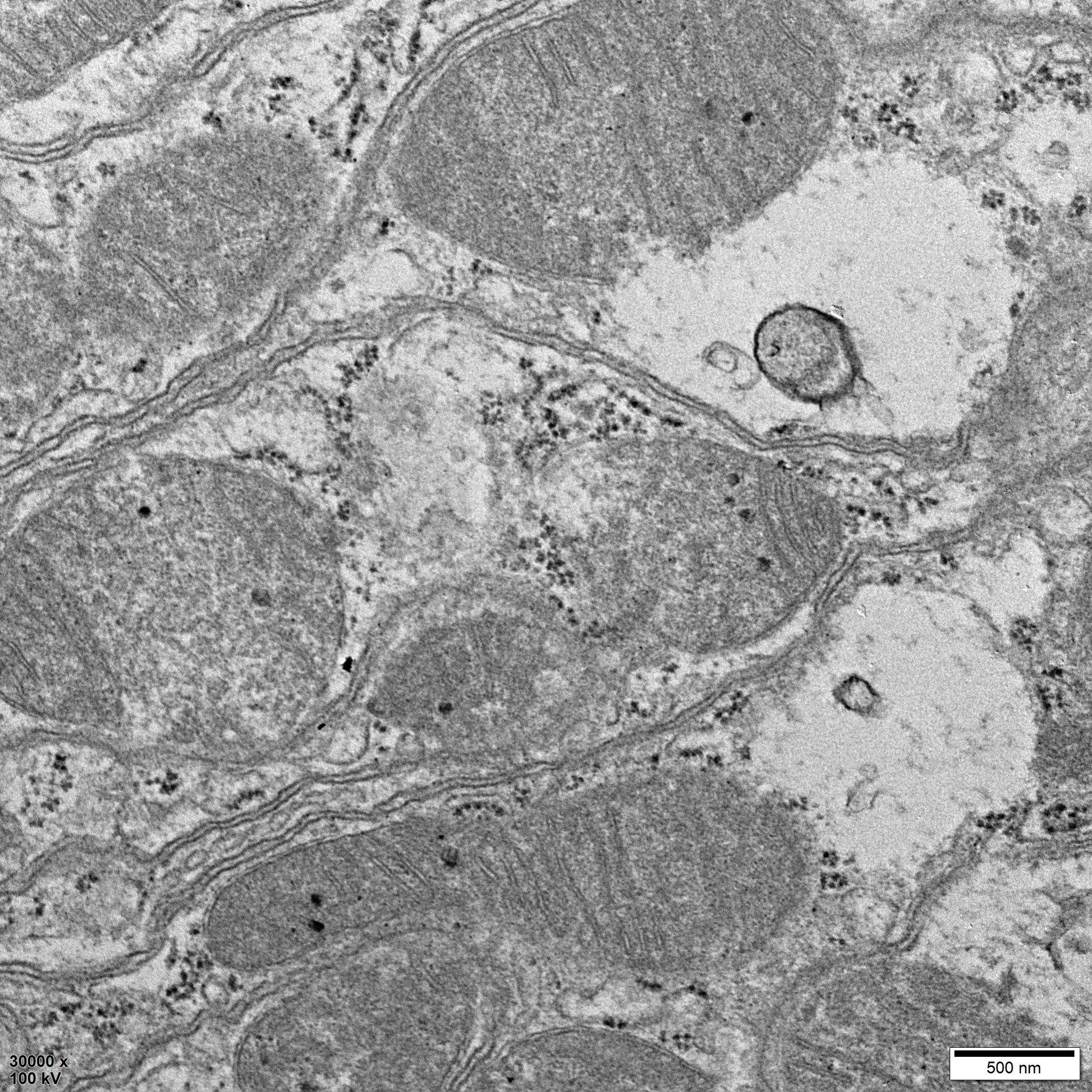

Supplement: Supplementary file 14 — Source data Fig. 9 [file 44321_2024_81_MOESM14_ESM.zip › Figure 9/9J/TEM/Sham.jpg]

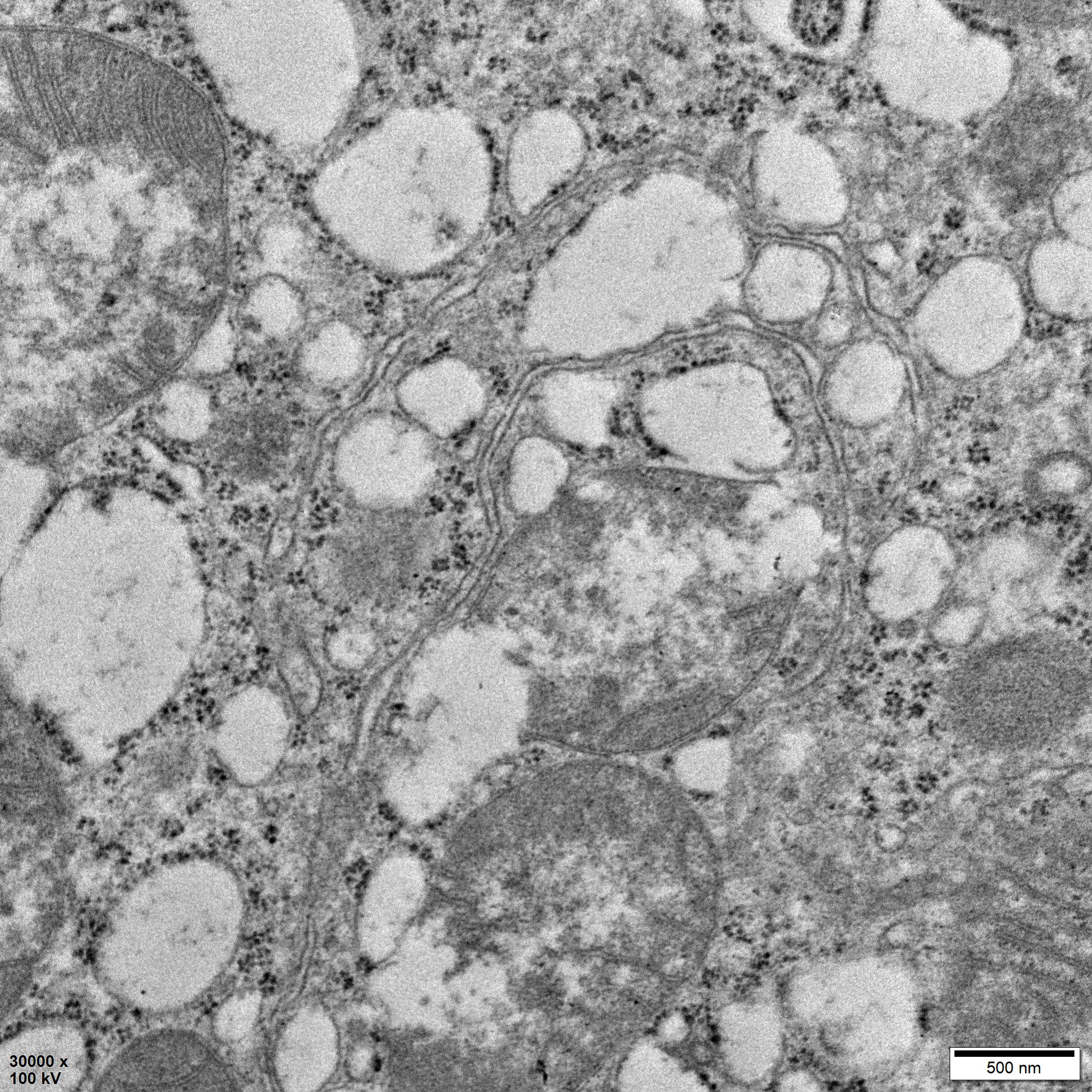

Supplement: Supplementary file 14 — Source data Fig. 9 [file 44321_2024_81_MOESM14_ESM.zip › Figure 9/9J/TEM/UUO.jpg]

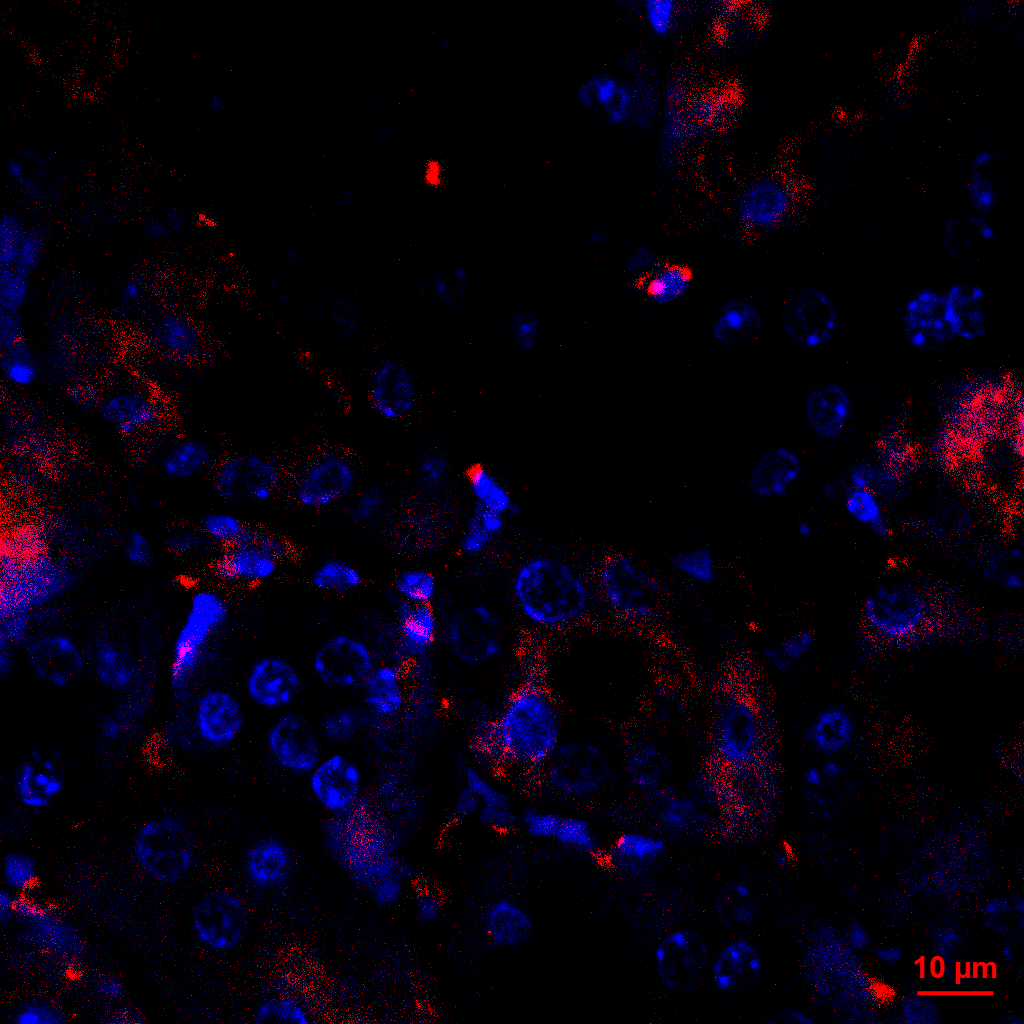

Supplement: Supplementary file 14 — Source data Fig. 9 [file 44321_2024_81_MOESM14_ESM.zip › Figure 9/9J/IF/Sham Calreticulin.tif]

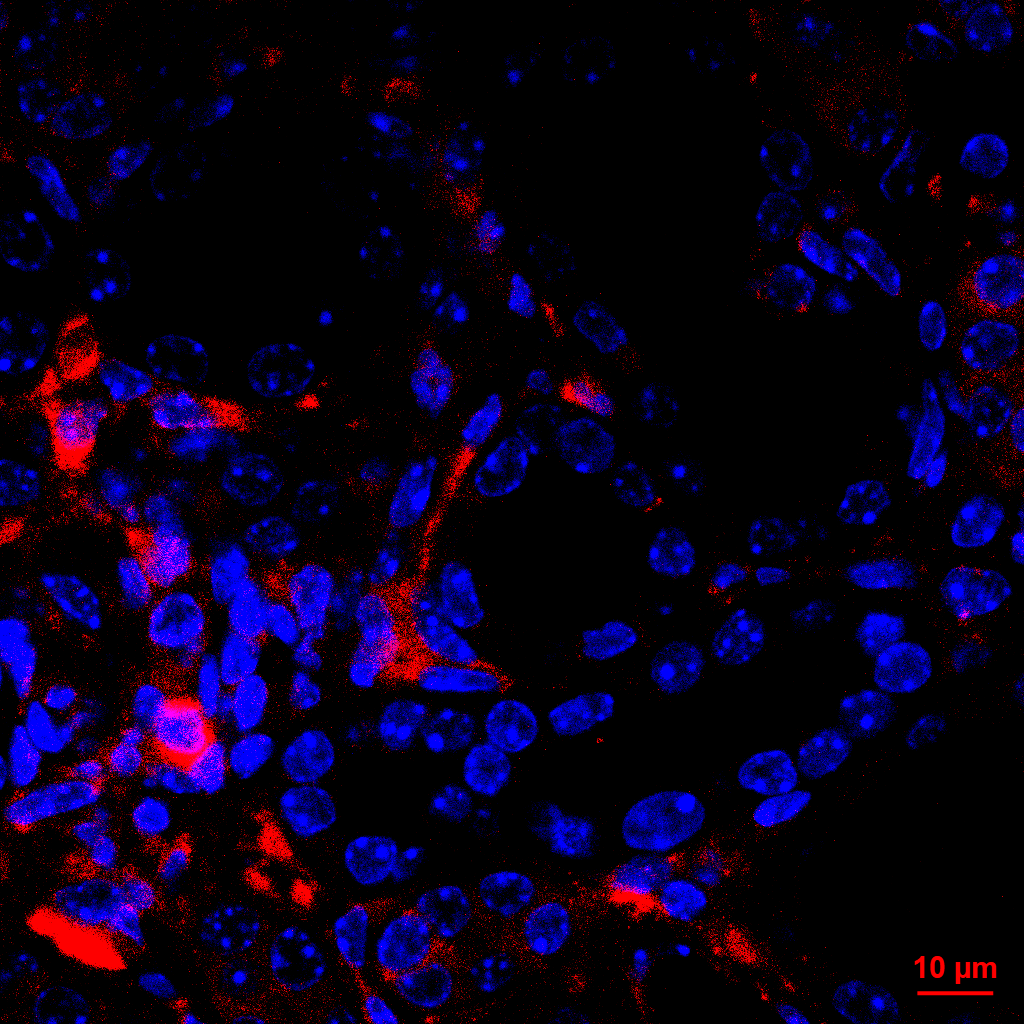

Supplement: Supplementary file 14 — Source data Fig. 9 [file 44321_2024_81_MOESM14_ESM.zip › Figure 9/9J/IF/UUO+Nicardipine Calreticulin.tif]

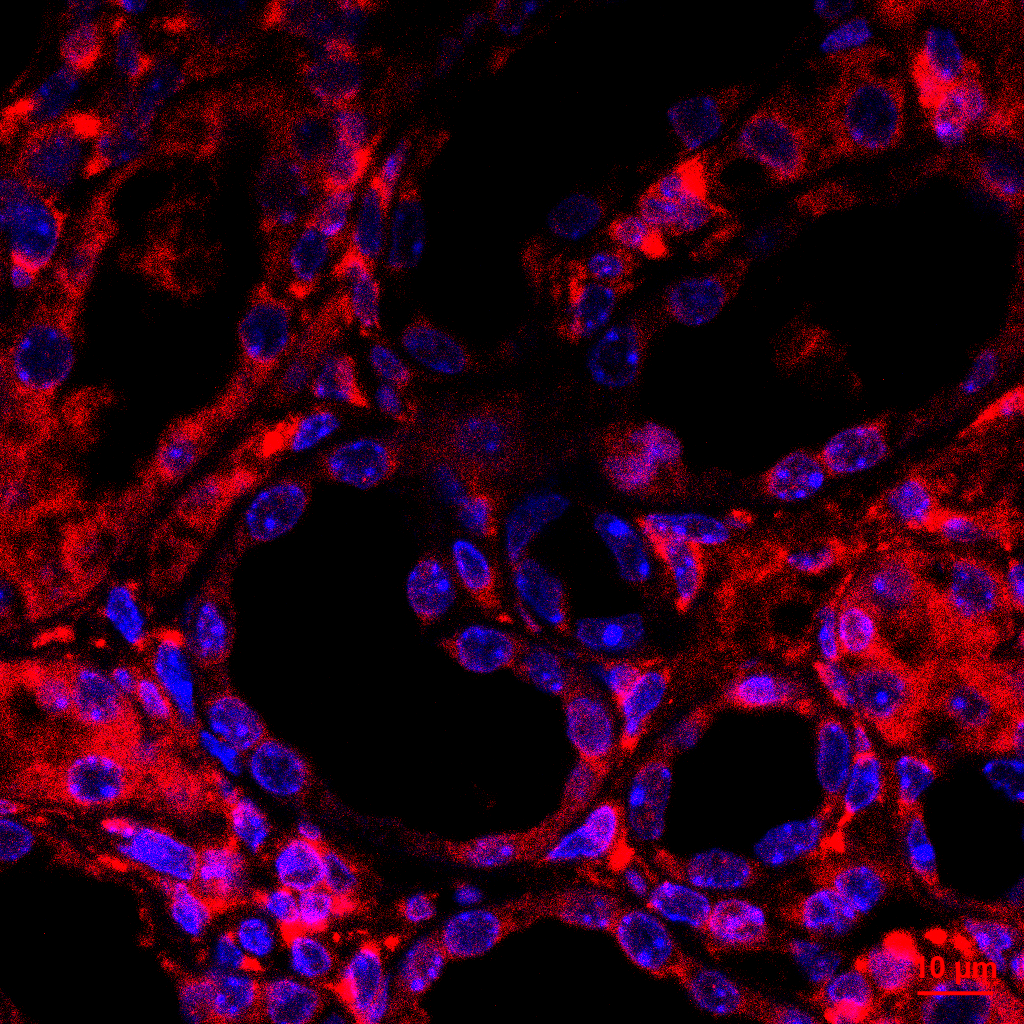

Supplement: Supplementary file 14 — Source data Fig. 9 [file 44321_2024_81_MOESM14_ESM.zip › Figure 9/9J/IF/UUO Calreticulin.tif]

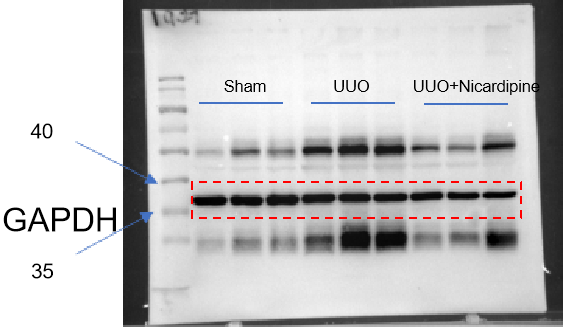

Supplement: Supplementary file 14 — Source data Fig. 9 [file 44321_2024_81_MOESM14_ESM.zip › Figure 9/9H/repeat/western GAPDH repeat.tif]

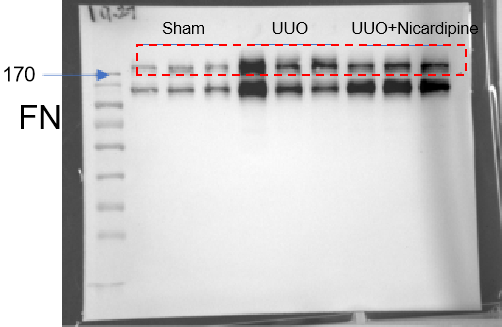

Supplement: Supplementary file 14 — Source data Fig. 9 [file 44321_2024_81_MOESM14_ESM.zip › Figure 9/9H/repeat/western FN repeat.tif]

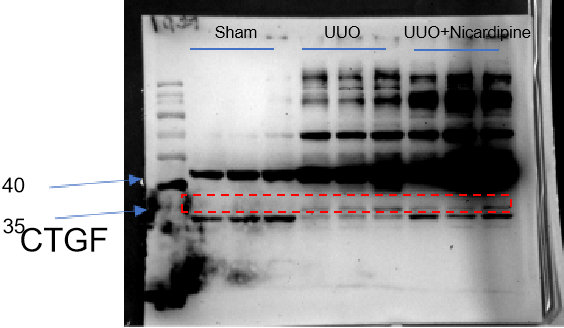

Supplement: Supplementary file 14 — Source data Fig. 9 [file 44321_2024_81_MOESM14_ESM.zip › Figure 9/9H/repeat/western CTGF repeat.tif]

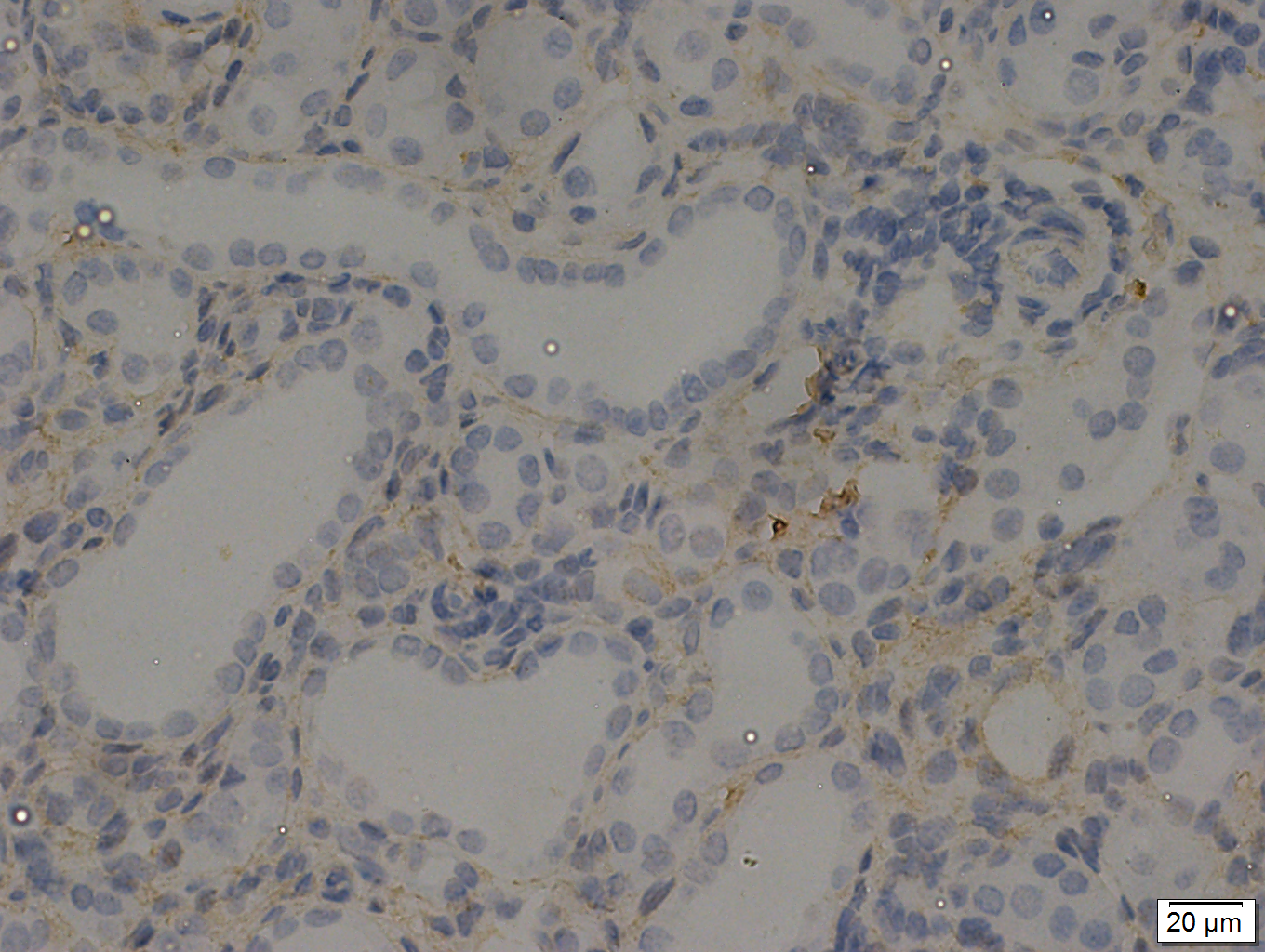

Supplement: Supplementary file 14 — Source data Fig. 9 [file 44321_2024_81_MOESM14_ESM.zip › Figure 9/9G/FN IHC/UUO+Nicardipine IHC.tif]

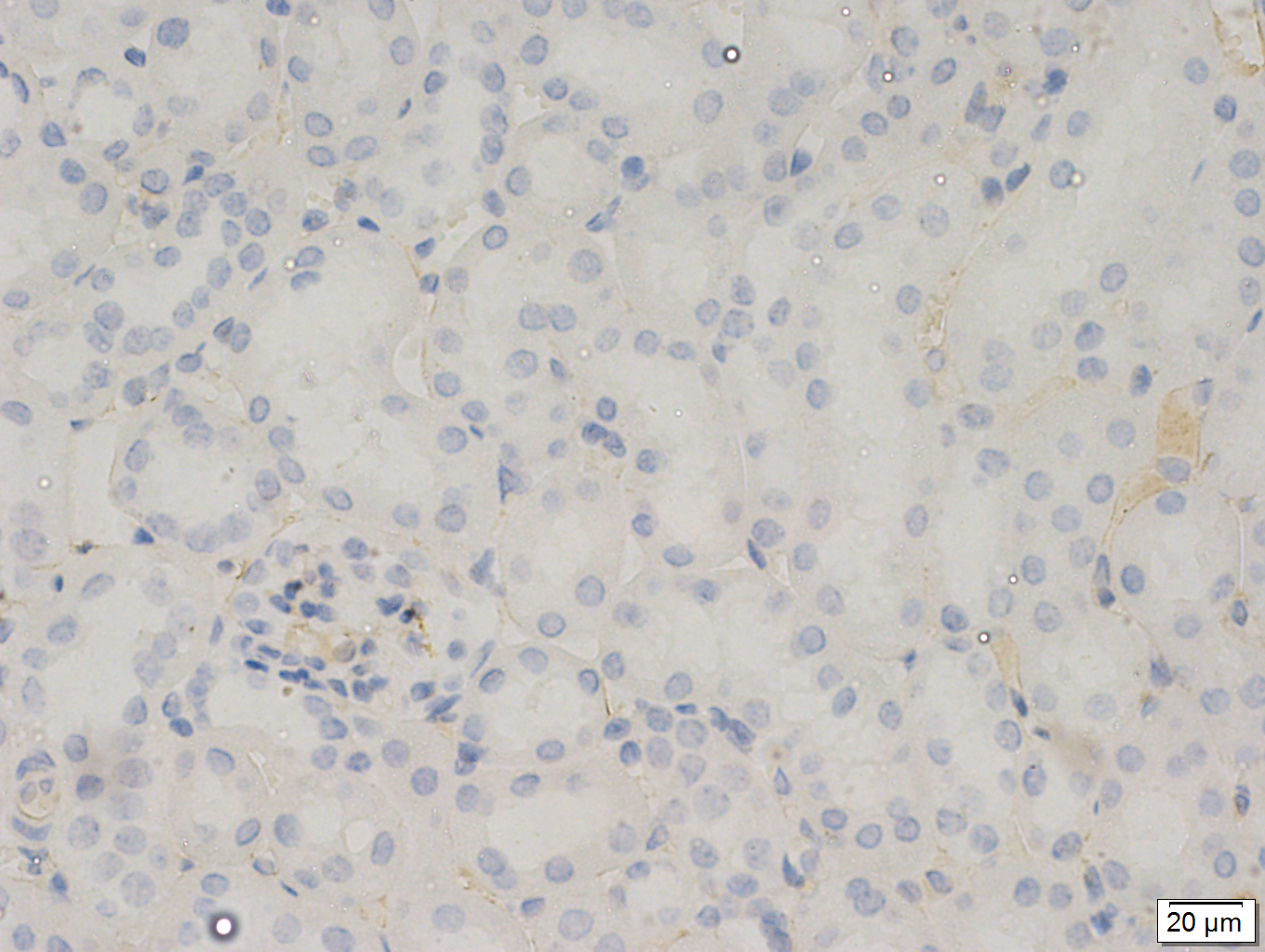

Supplement: Supplementary file 14 — Source data Fig. 9 [file 44321_2024_81_MOESM14_ESM.zip › Figure 9/9G/FN IHC/Sham IHC.tif]

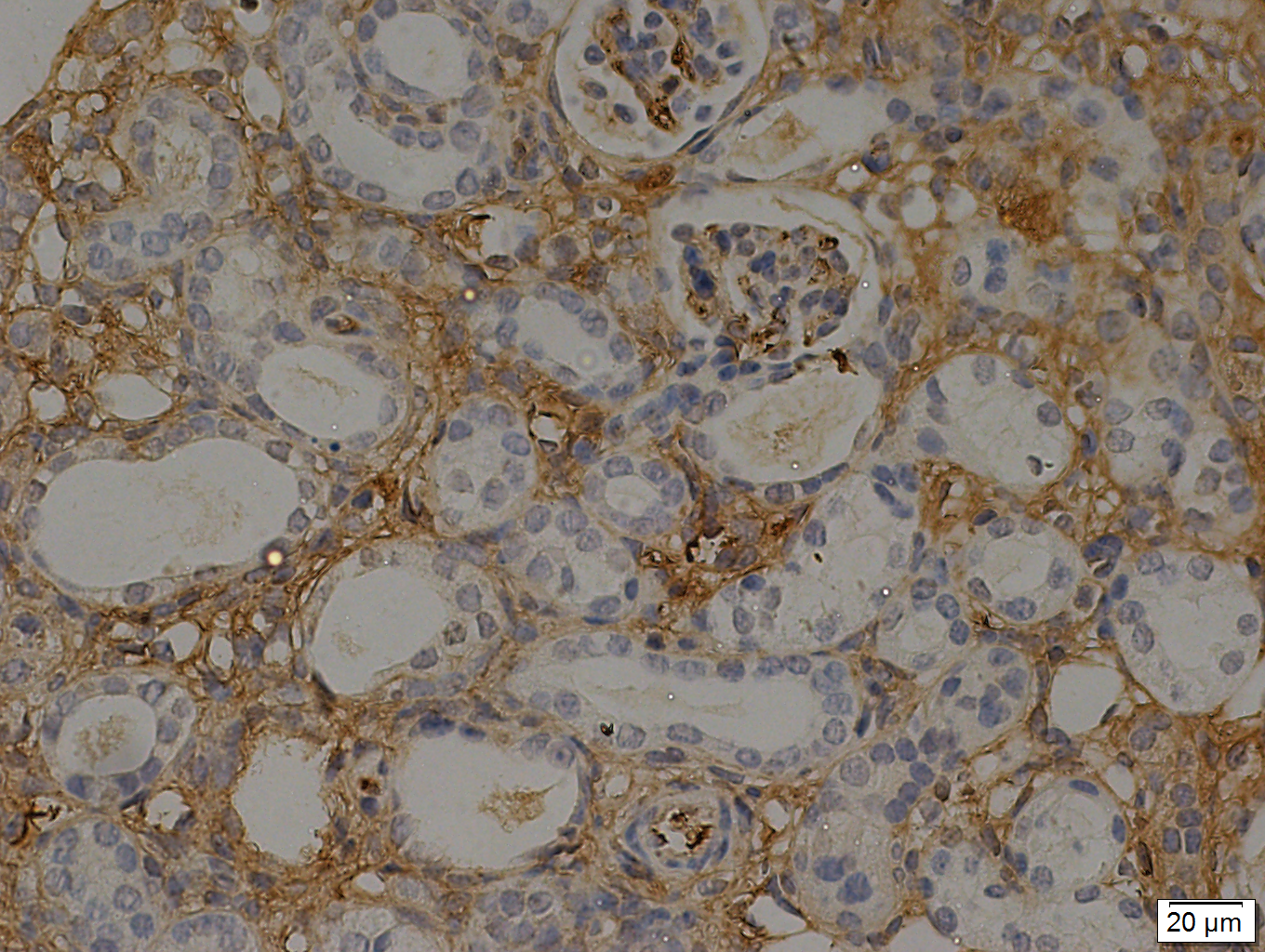

Supplement: Supplementary file 14 — Source data Fig. 9 [file 44321_2024_81_MOESM14_ESM.zip › Figure 9/9G/FN IHC/UUO IHC.tif]

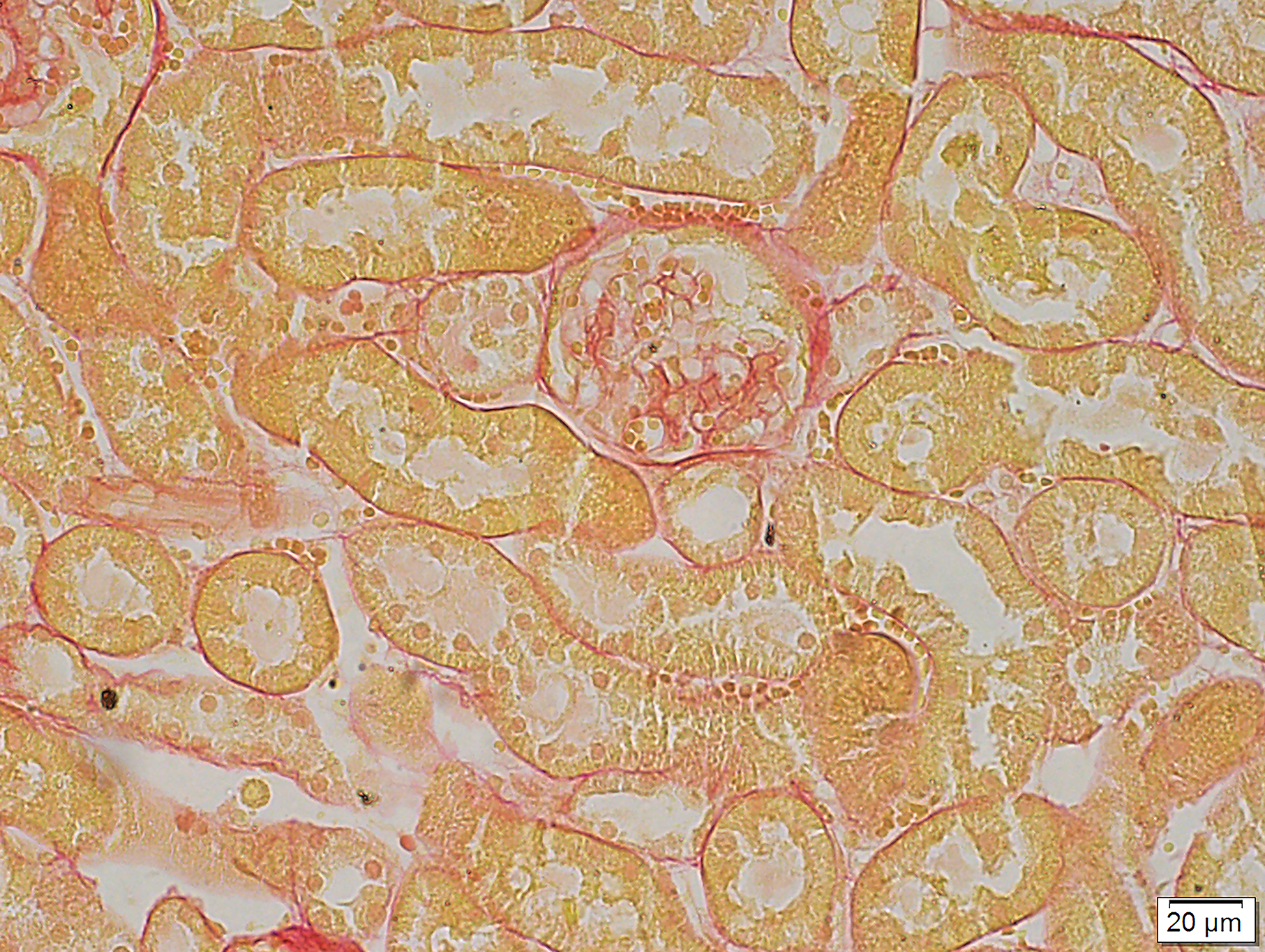

Supplement: Supplementary file 14 — Source data Fig. 9 [file 44321_2024_81_MOESM14_ESM.zip › Figure 9/9G/Sirius red/Sham.tif]

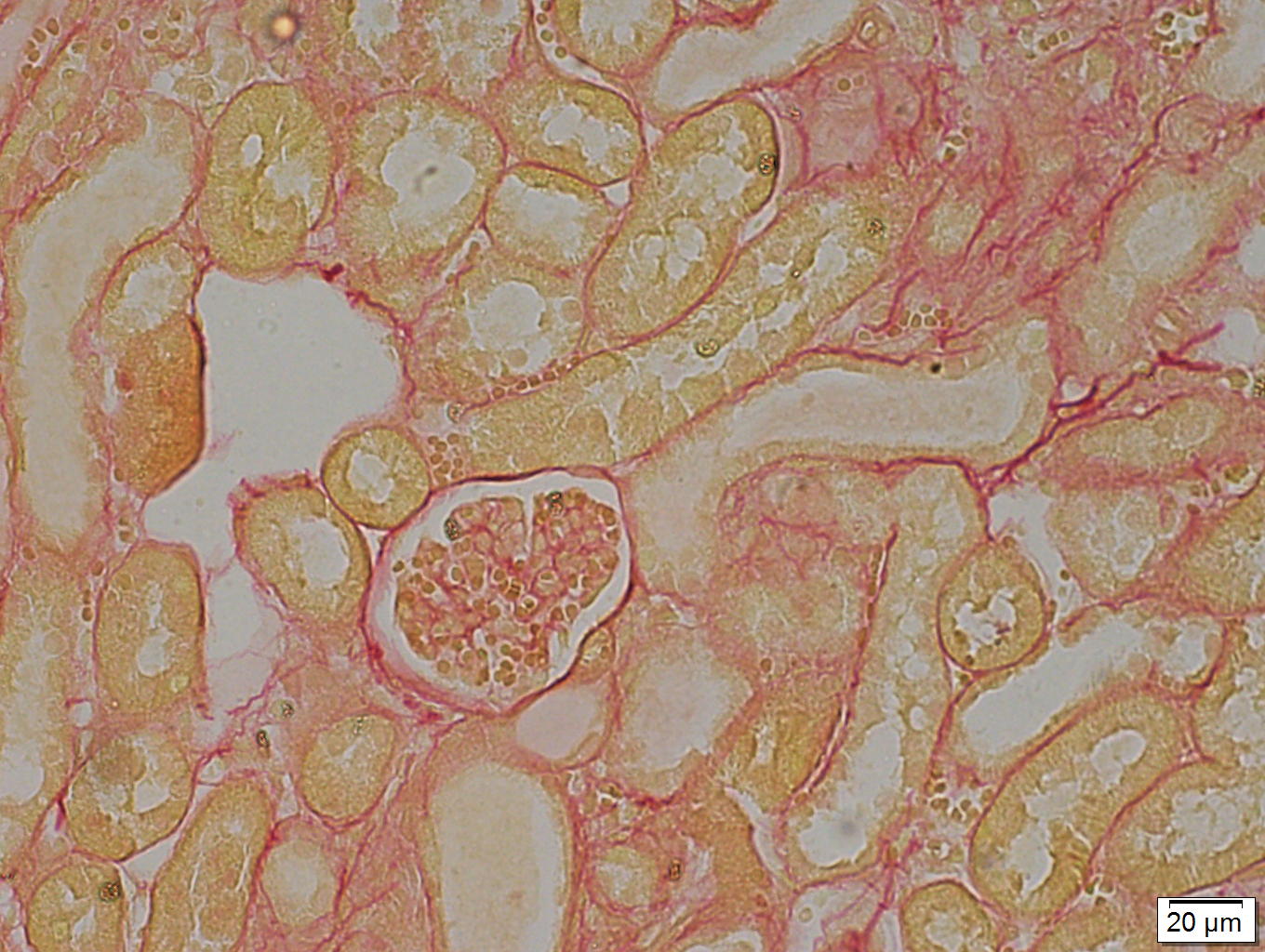

Supplement: Supplementary file 14 — Source data Fig. 9 [file 44321_2024_81_MOESM14_ESM.zip › Figure 9/9G/Sirius red/UUO+Nicardipine.tif]

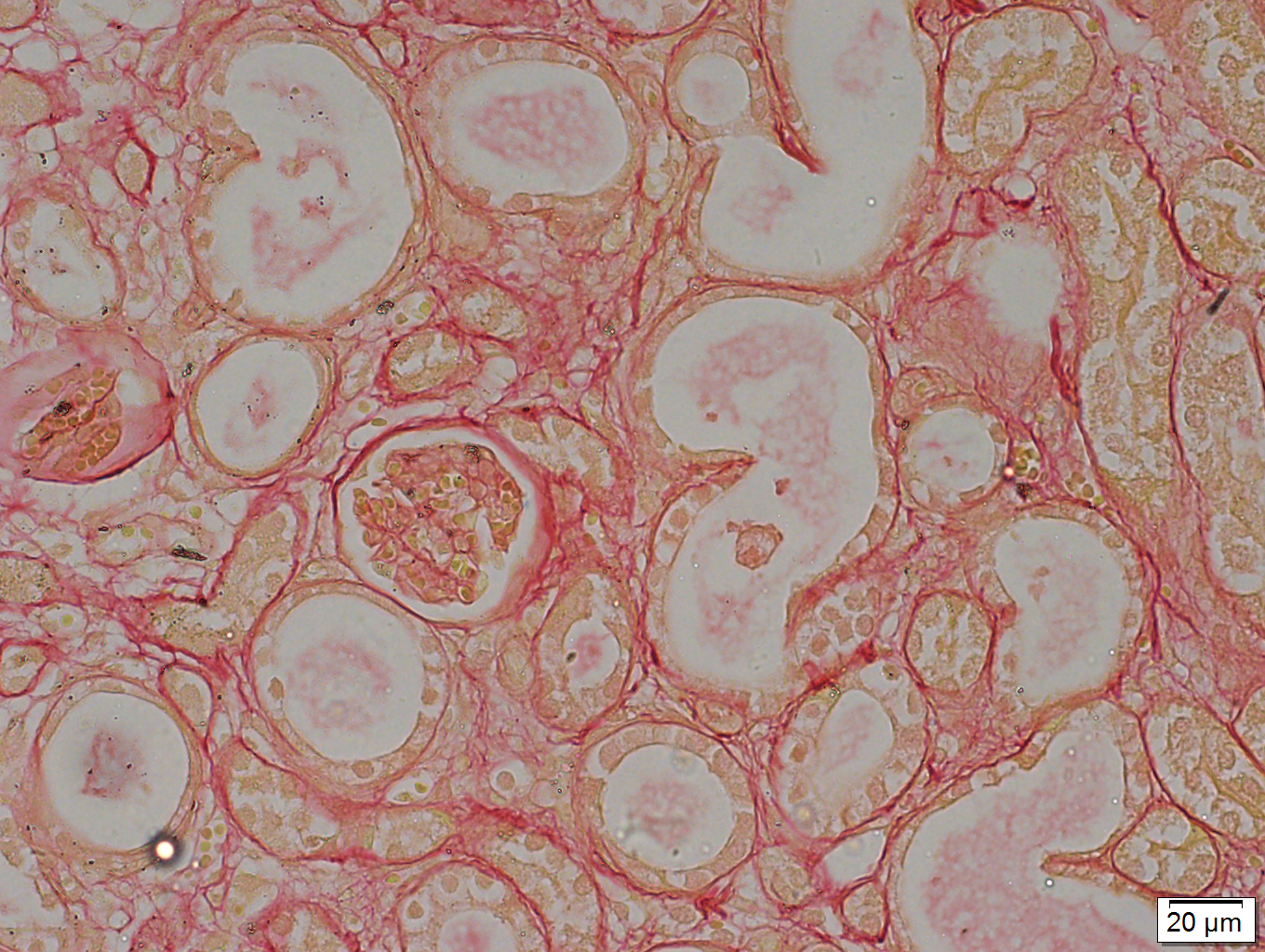

Supplement: Supplementary file 14 — Source data Fig. 9 [file 44321_2024_81_MOESM14_ESM.zip › Figure 9/9G/Sirius red/UUO.tif]
