# Supplementary material for: Tubular insulin-induced gene 1 deficiency promotes NAD+ consumption and exacerbates kidney fibrosis
Source: EMBO Mol Med. 2024 May 28;16(7):11. doi: 10.1038/s44321-024-00081-7 (PMC11251182; doi:10.1038/s44321-024-00081-7)
Supplement: Supplementary file 16 — Expanded View Figures [file 44321_2024_81_MOESM16_ESM.pdf]

Expanded View Figures

Figure EV1. Re-analysis of scRNA-seq dataset GSE182256 and the expression of ER stress indexes and Insig1 in the CKD model.

(**A**) UMAP dimension Reduction showing 28 distinct cell types in Control and UUO kidneys identified by unsupervised clustering. GEC, glomerular endothelial cell; Endo, endothelial; Podo, podocyte; ALOH, ascending loop of Henle; DCT, distal convoluted tubule; CNT, connecting tubule; CD PC, collecting duct principal cell; A-IC, α intercalated cell; B-IC, β intercalated cell; Trans-IC, transitional intercalated cell; Neutro, neutrophil; Mono, monocyte; Macro, macrophage; Baso, basophil; B Lymph, B lymphocyte; Prolif Ly, proliferating lymphocyte; Prolif PT, proliferating PT. (**B**) Bubble plots showing the expression of cell cluster marker genes in UUO kidney. (**C**) Bubble plots showing the expression of PTCs cluster marker genes in UUO kidney. (**D**) qPCR analysis of *Perk*, *Trib3*, and *Atf6* in the kidneys of UUO model ($n = 5$, in each group, biological replicates). (**E**) qPCR analysis of *Atf4* in the kidneys of 5/6 Nx model ($n = 6$ in Sham group; $n = 5$ in 5/6 Nx group, biological replicates). (**F**) The violin plot showed the Insig1 expression levels in each type of normal kidney cells ($n = 6$ in Control group; $n = 2$ in UUO group). (**G**) The Insig1 expression changes in each PT subsets in UUO and Control groups. (**H**, **I**) Representative images and quantification of IHC (INSIG1) in kidneys of UUO model ($n = 5$ in Sham group; $n = 7$ in UUO group, biological replicates; scale bars, 20 μm). (**J**) qPCR analysis of *Insig1* in the kidneys of UUO model ($n = 6$ in Sham group; $n = 7$ in UUO group, biological replicates). (**K**) Representative immunoblot bands and quantification of INSIG1 in the kidneys of UUO model ($n = 6$, in each group, biological replicates). Data information: In (**D**, **I**), Data are represented as mean ± SD. Student's *t* test. (**E**, **J**, **K**) Data are represented as mean ± SD. Mann–Whitney test. Source data are available online for this figure.

© The Author(s)

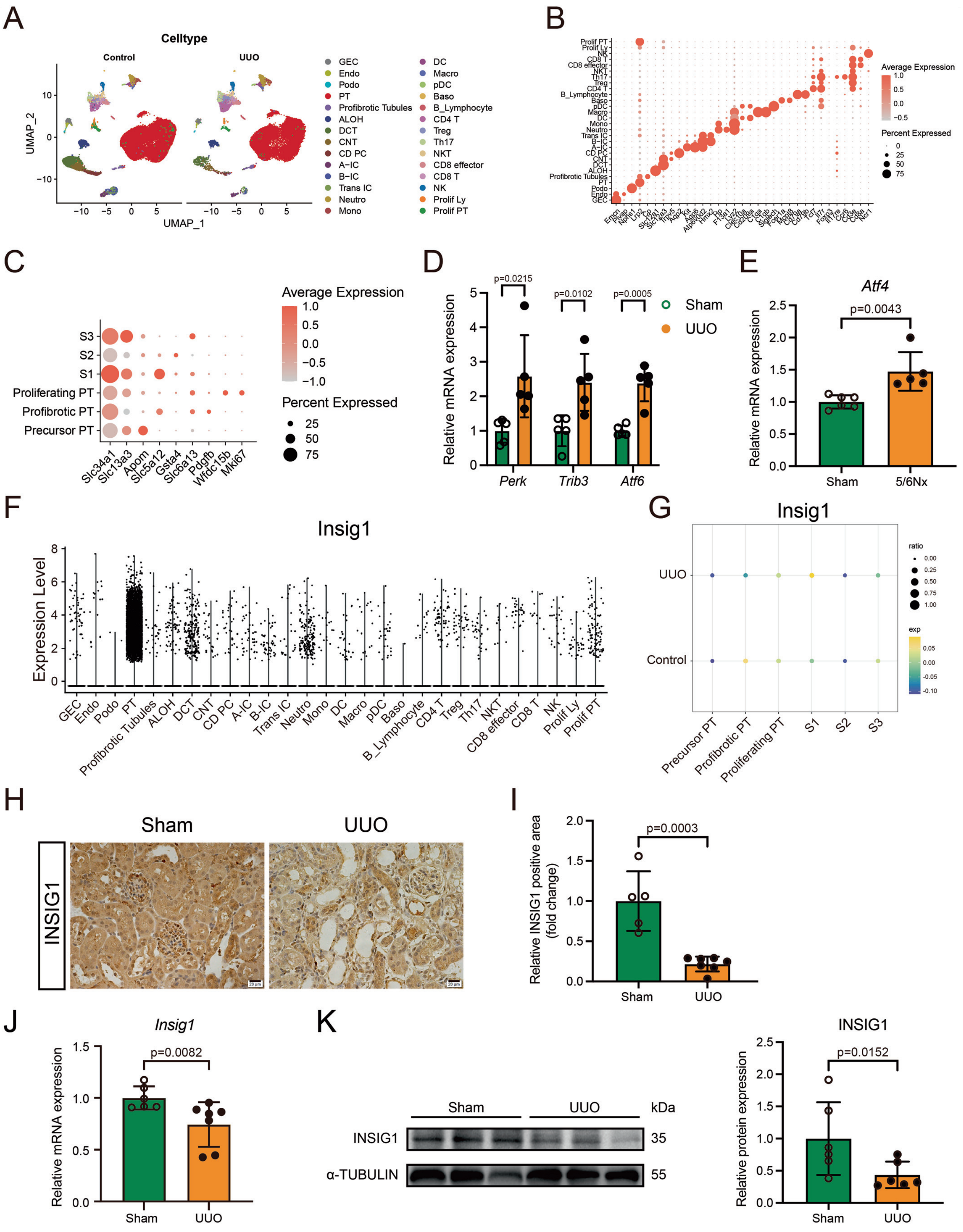
A
Celltype
Control
UUO
UMAP_2
UMAP_1
GEC
Endo
Podo
PT
Profibrotic Tubules
ALOH
DCT
CNT
CD PC
A−IC
B−IC
Trans IC
Neutro
Mono
DC
Macro
pDC
Baso
B_Lymphocyte
CD4 T
Treg
Th17
NKT
CD8 effector
CD8 T
NK
Prolif Ly
Prolif PT
B
Average Expression
Percent Expressed
C
Average Expression
Percent Expressed
S3
S2
S1
Proliferating PT
Profibrotic PT
Precursor PT
Slc34a1
Slc13a3
Apom
Slc5a12
Gsta4
Slc6a13
Pdgfb
Wfdc15b
Mki67
D
Relative mRNA expression
p=0.0215
p=0.0102
p=0.0005
Sham
UUO
Perk
Trib3
Atf6
E
Atf4
p=0.0043
Relative mRNA expression
Sham
5/6Nx
F
Insig1
Expression Level
G
Insig1
UUO
Control
ratio
exp
Precursor PT
Profibrotic PT
Proliferating PT
S1
S2
S3
H
Sham
UUO
INSIG1
I
Relative INSIG1 positive area (fold change)
p=0.0003
Sham
UUO
J
Insig1
p=0.0082
Relative mRNA expression
Sham
UUO
K
Sham
UUO
kDa
INSIG1
35
α-TUBULIN
55
INSIG1
p=0.0152
Relative protein expression
Sham
UUO

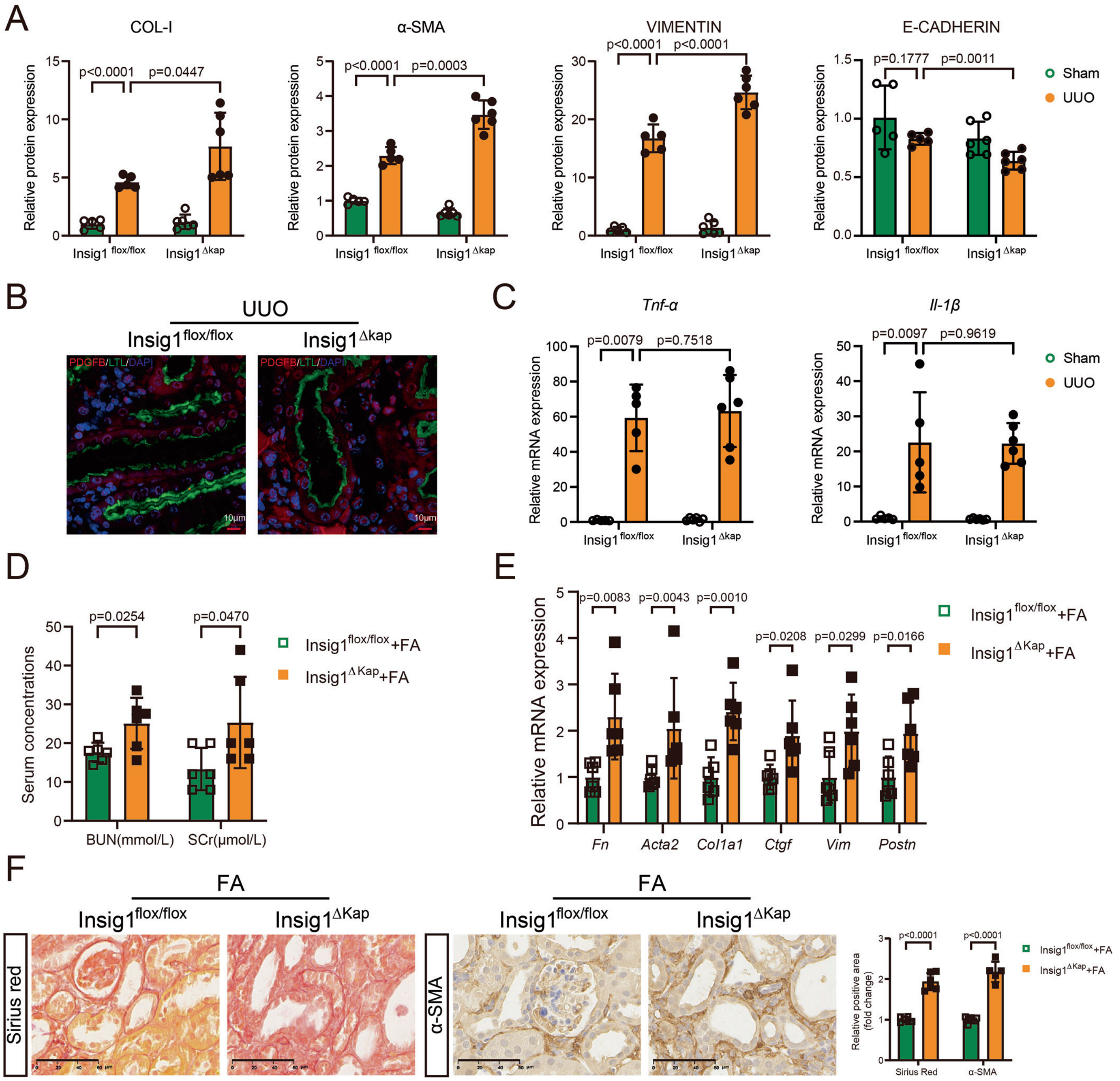

Figure EV2. Insig1 loss in PTCs exacerbated UUO-induced and FA-induced kidney fibrosis.

(**A**) Quantification of COL-I, α-SMA, VIMENTIN, and E-CADHERIN in Fig. 2F ($n = 5$ in Insig1$^{flox/flox}$ group and Insig1$^{flox/flox}$ + UUO group; $n = 6$ in Insig1$^{\Delta Kap}$ group and Insig1$^{\Delta Kap}$ + UUO group, biological replicates). (**B**) Representative images of PDGFB staining in Insig1$^{flox/flox}$ + UUO and Insig1$^{\Delta Kap}$ + UUO groups ($n = 3$ in each group, biological replicates; scale bars, 10 μm; red: PDGFB; green: LTL; blue: DAPI). (**C**) qPCR analysis of *Tnf*-α and *Il*-1β in the kidneys of Insig1$^{flox/flox}$ and Insig1$^{\Delta Kap}$ mice after UUO ($n = 5$ in Insig1$^{flox/flox}$ group and Insig1$^{flox/flox}$ + UUO group; $n = 6$ in Insig1$^{\Delta Kap}$ group and Insig1$^{\Delta Kap}$ + UUO group, biological replicates). (**D**) SCr and BUN concentrations were measured in FA-treated Insig1$^{flox/flox}$ and Insig1$^{\Delta Kap}$ mice ($n = 6$ in each group, biological replicates). (**E**) qPCR analysis of *Fn*, *Acta2*, *Col1a1*, *Ctgf*, *Vim*, and *Postn* in the kidneys of FA-treated Insig1$^{flox/flox}$ and Insig1$^{\Delta Kap}$ mice ($n = 6$ in each group, biological replicates). (**F**) Representative images and quantification of Sirius red staining and IHC (α-SMA) staining in mouse kidneys (scale bars, 60 μm; $n = 5$–6 in each group, biological replicates). Data information: In (**A**, **D**, **F**), Data are represented as mean ± SD. Student's *t* test. (**C**, **E**) Data are represented as mean ± SD. Student's *t* test or Mann–Whitney test. Source data are available online for this figure.

 © The Author(s)

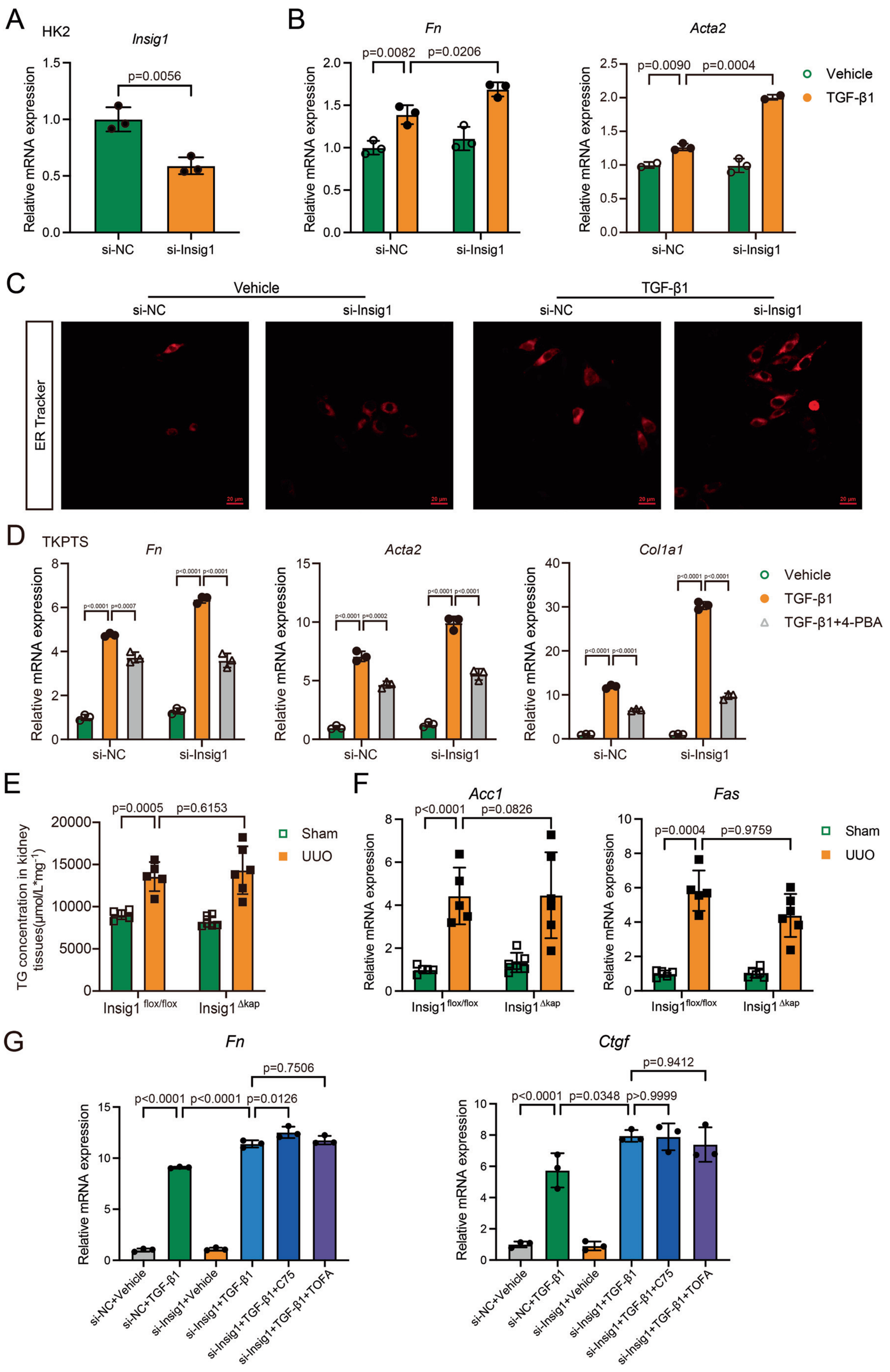
A
HK2
Insig1
p=0.0056
Relative mRNA expression
si-NC
si-Insig1
B
Fn
p=0.0082
p=0.0206
Acta2
p=0.0090
p=0.0004
Vehicle
TGF-β1
C
Vehicle
TGF-β1
si-NC
si-Insig1
ER Tracker
20 μm
D
TKPTS
Fn
Acta2
Col1a1
p<0.0001
p=0.0007
p=0.0002
TGF-β1+4-PBA
E
p=0.0005
p=0.6153
TG concentration in kidney tissues(μmol/L*mg-1)
Sham
UUO
Insig1 flox/flox
Insig1 Δkap
F
Acc1
p<0.0001
p=0.0826
Fas
p=0.0004
p=0.9759
G
Fn
p=0.7506
p<0.0001
p=0.0126
Ctgf
p=0.9412
p=0.0348
p>0.9999
si-NC+Vehicle
si-NC+TGF-β1
si-Insig1+Vehicle
si-Insig1+TGF-β1
si-Insig1+TGF-β1+C75
si-Insig1+TGF-β1+TOFA

Figure EV3. The profibrotic effect of Insig1 deletion in PTCs was not be correlated with lipogenic activity.

(**A**) qPCR analysis of *Insig1* in Insig1 silencing HK2 cells ($n = 3$ in each group, biological replicates). (**B**) qPCR analysis of *Fn* and *Acta2* in Insig1 silencing HK2 cells stimulated by TGF-β1 ($n = 2$–3 in each group, biological replicates). (**C**) Representative images of ER tracker staining in Insig1 silencing HK2 cells stimulated by TGF-β1 (scale bars, 20 μm; $n = 3$ in each group, biological replicates). (**D**) qPCR analysis of *Fn*, *Acta2* and *Col1a1* in Insig1 silencing TKPTS cells stimulated by TGF-β1 combined with 4-PBA ($n = 3$ in each group, biological replicates). (**E**) TG concentration was measured in the kidneys of $Insig1^{flox/flox}$ and $Insig1^{\Delta Kap}$ mice after UUO ($n = 5$ in $Insig1^{flox/flox}$ group and $Insig1^{flox/flox}$ + UUO group; $n = 6$ in $Insig1^{\Delta Kap}$ group and $Insig1^{\Delta Kap}$ + UUO group, biological replicates). (**F**) qPCR analysis of *Acc1* and *Fas* in the kidneys of $Insig1^{flox/flox}$ and $Insig1^{\Delta Kap}$ mice subjected to UUO ($n = 5$ in $Insig1^{flox/flox}$ group and $Insig1^{flox/flox}$ + UUO group; $n = 6$ in $Insig1^{\Delta Kap}$ group and $Insig1^{\Delta Kap}$ + UUO group, biological replicates). (**G**) qPCR analysis of *Fn* and *Ctgf* in Insig1 silencing TKPTS cells stimulated by TGF-β1 combined with C75 or TOFA ($n = 3$ in each group, biological replicates). Data information: In (**A**, **B**, **E**, **F**), Data are represented as mean ± SD. Student's *t* test. (**D**, **G**) Data are represented as mean ± SD. One-way ANOVA. Source data are available online for this figure.

 © The Author(s)

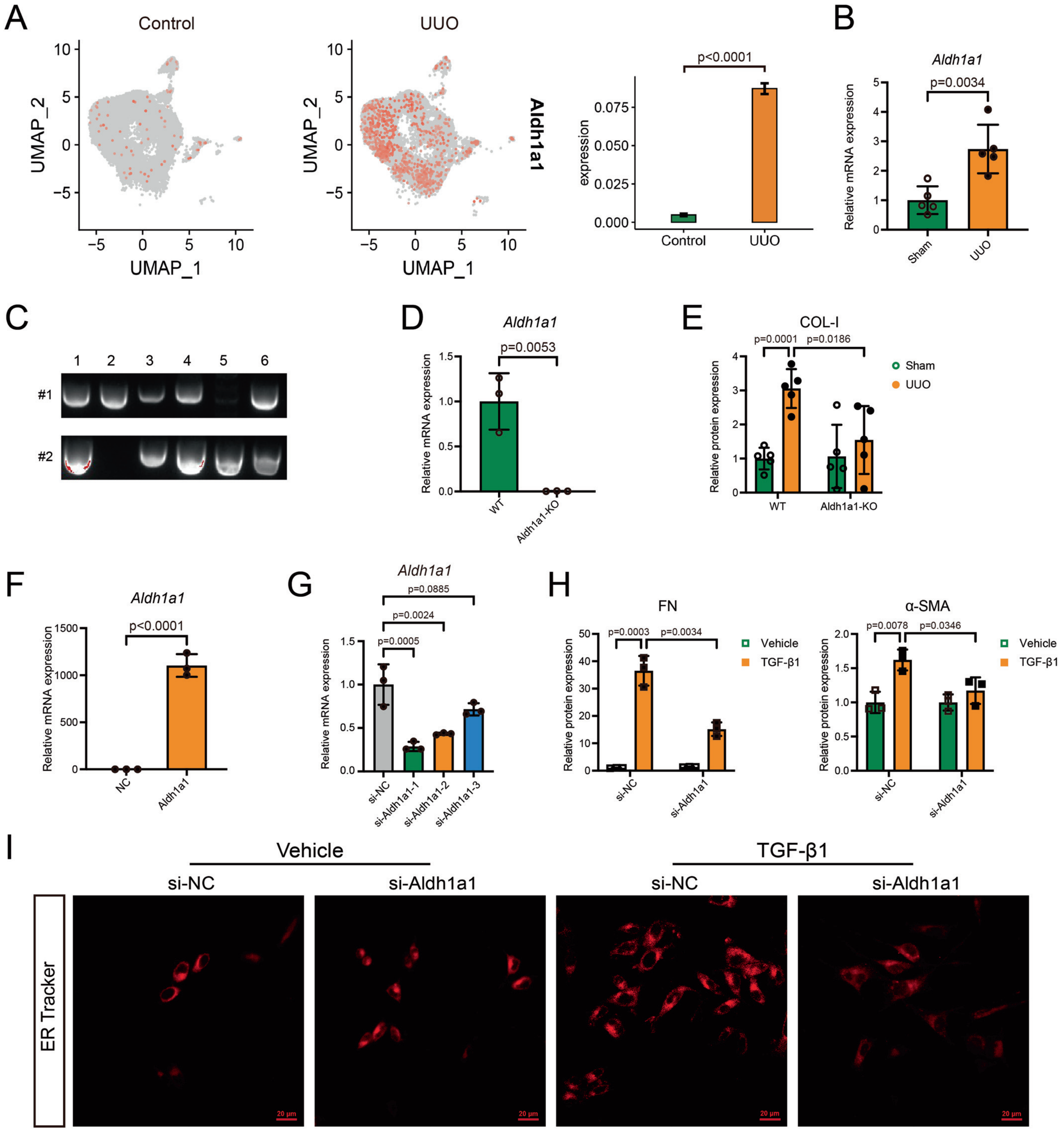

Figure EV4. The expression of Aldh1a1 in UUO model and Aldh1a1 silencing ameliorated TGF-β1-induced fibrotic responses and ER stress in vitro.

(**A**) UMAP dimension Reduction showing Aldh1a1 expression in Control and UUO kidneys ($n = 6$ in Control group; $n = 2$ in UUO group). (**B**) qPCR analysis of *Aldh1a1* in the kidneys of UUO model ($n = 5$ in each group, biological replicates). (**C**) PCR analysis of *Aldh1a1* for the genetic identification of Aldh1a1-KO mice. (HET: 1, 3, 4, 6; HOM: 2; WT: 5). (**D**) qPCR analysis of *Aldh1a1* in the kidneys of WT and Aldh1a1-KO mice ($n = 3$ in each group, biological replicates). (**E**) Quantification of COL-I in Fig. 7E ($n = 5$ in each group, biological replicates). (**F**) qPCR analysis of *Aldh1a1* in Aldh1a1 overexpressed TKPTS cells ($n = 3$ in each group, biological replicates). (**G**) qPCR analysis of *Aldh1a1* in Aldh1a1 silencing TKPTS cells (si-Aldh1a1-1 was selected for additional experimentation) ($n = 3$ in each group, biological replicates). (**H**) Quantification of FN and α-SMA in Fig. 7L ($n = 3$ in each group, biological replicates). (**I**) Representative images of ER tracker staining in Aldh1a1 silencing TKPTS cells stimulated by TGF-β1 (scale bars, 20 μm; $n = 3$ in each group, biological replicates). Data information: In (**B**, **D**, **E**, **F**, **H**), Data are represented as mean ± SD. Student's *t* test. (**G**) Data are represented as mean ± SD. One-way ANOVA. Source data are available online for this figure.

© The Author(s)

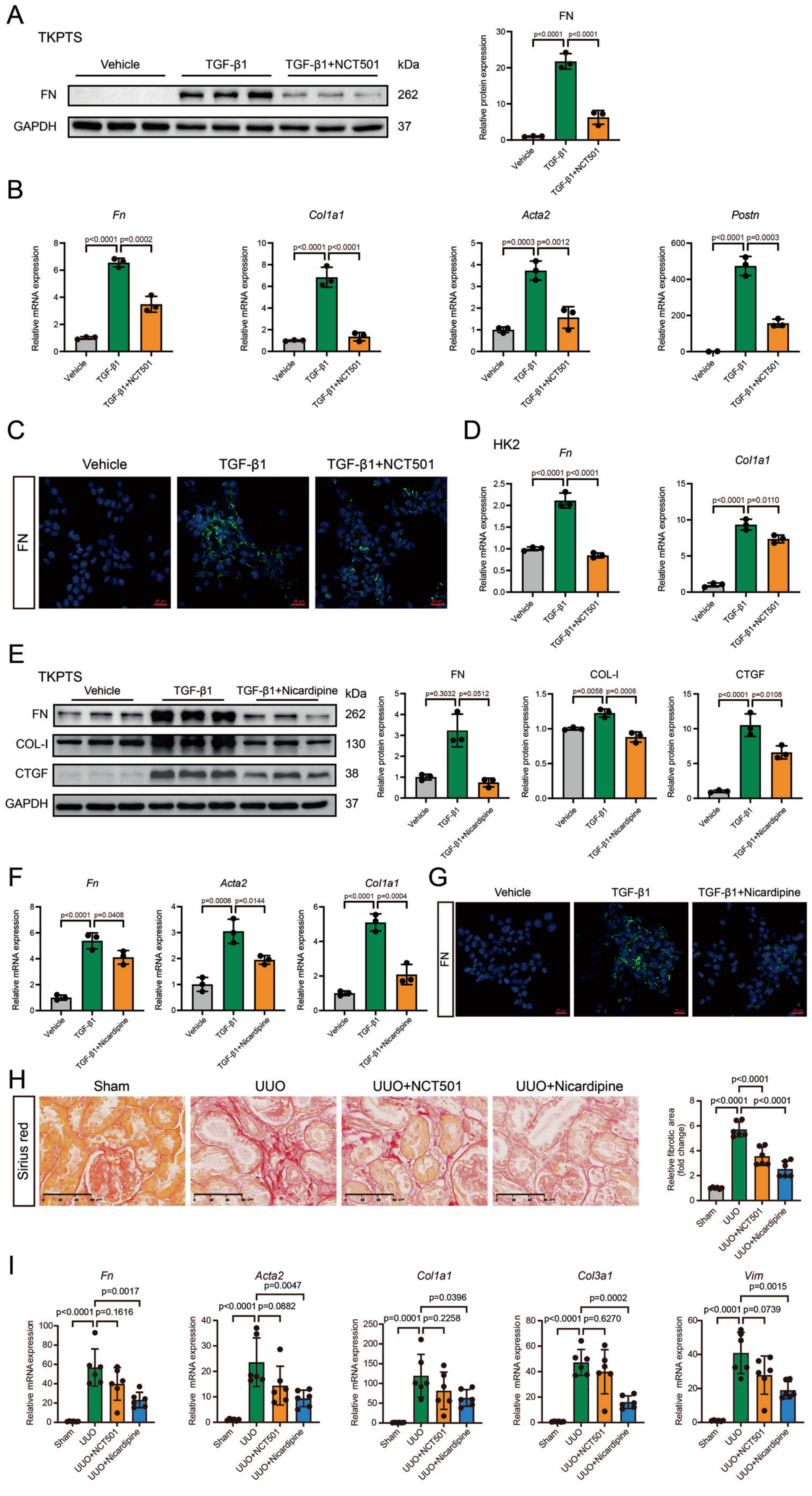
A
TKPTS
Vehicle
TGF-β1
TGF-β1+NCT501
kDa
FN
262
GAPDH
37
FN
Relative protein expression
p<0.0001 p<0.0001
B
Fn
Col1a1
Acta2
Postn
Relative mRNA expression
p<0.0001 p=0.0002
p<0.0001 p<0.0001
p=0.0003 p=0.0012
p<0.0001 p=0.0003
C
Vehicle
TGF-β1
TGF-β1+NCT501
FN
D
HK2
Fn
Col1a1
p<0.0001 p<0.0001
p<0.0001 p=0.0110
E
TKPTS
Vehicle
TGF-β1
TGF-β1+Nicardipine
kDa
FN 262
COL-I 130
CTGF 38
GAPDH 37
FN
COL-I
CTGF
p=0.3032 p=0.0512
p=0.0058 p=0.0006
p<0.0001 p=0.0108
F
Fn
Acta2
Col1a1
p<0.0001 p=0.0408
p=0.0006 p=0.0144
p<0.0001 p=0.0004
G
Vehicle
TGF-β1
TGF-β1+Nicardipine
FN
H
Sham
UUO
UUO+NCT501
UUO+Nicardipine
Sirius red
Relative fibrotic area (fold change)
p<0.0001
p<0.0001 p<0.0001
I
Fn
p=0.0017
p<0.0001 p=0.1616
Acta2
p=0.0047
p<0.0001 p=0.0882
Col1a1
p=0.0396
p=0.0001 p=0.2258
Col3a1
p=0.0002
p<0.0001 p=0.6270
Vim
p=0.0015
p<0.0001 p=0.0739
Relative mRNA expression
Sham
UUO
UUO+NCT501
UUO+Nicardipine

 © The Author(s)

Figure EV5. NCT501 and nicardipine improved renal fibrosis in vivo and in vitro.

(**A**) Representative immunoblot bands and quantification of FN in TGF-β1-treated TKPTS cells stimulated with NCT501 ($n = 3$ in each group, biological replicates). (**B**) qPCR analysis of *Fn*, *Acta2*, *Col1a1* and *Postn* in TGF-β1-treated TKPTS cells stimulated with NCT501 ($n = 2$–3 in each group, biological replicates). (**C**) Representative images of FN staining in TGF-β1-treated TKPTS cells stimulated with NCT501 (scale bars, 20 μm; $n = 3$ in each group, biological replicates). (**D**) qPCR analysis of *Fn* and *Col1a1* in TGF-β1-treated HK2 cells stimulated with NCT501 ($n = 3$ in each group, biological replicates). (**E**) Representative immunoblot bands and quantification of FN, COL-I, and CTGF in TGF-β1-treated TKPTS cells with or without nicardipine treatment ($n = 3$ in each group, biological replicates). (**F**) qPCR analysis for *Fn*, *Acta2*, and *Col1a1* in TGF-β1-treated TKPTS cells with or without nicardipine treatment ($n = 3$ in each group, biological replicates). (**G**) Representative images of FN staining in TGF-β1-treated TKPTS cells with or without nicardipine treatment (scale bars, 20 μm; $n = 3$ in each group, biological replicates). (**H**) Representative images and quantification of Sirius red staining in mouse kidneys (scale bars, 60 μm; $n = 6$ in each group, biological replicates). (**I**) qPCR analysis for *Fn*, *Acta2*, *Col1a1, Col3a1* and *Vim* in mouse kidneys ($n = 6$ in each group, biological replicates). Data information: Data are represented as mean ± SD. One-way ANOVA. Source data are available online for this figure.

© The Author(s)
